# Alternative splicing-derived intersectin1-L and intersectin1-S exert opposite function in glioma progression

**DOI:** 10.1038/s41419-019-1668-0

**Published:** 2019-06-03

**Authors:** Ying Shao, Wei Chong, Xiaoli Liu, Yun Xu, Huikun Zhang, Qiao Xu, Zhifang Guo, Yawen Zhao, Ming Zhang, Yongjie Ma, Feng Gu

**Affiliations:** 1 Department of Breast Cancer Pathology and Research Laboratory, Tianjin Medical University Cancer Institute and Hospital, Tianjin, China; 2 Department of Tumor Cell Biology, Tianjin Medical University Cancer Institute and Hospital, National Clinical Research Center for Cancer, Tianjin, China; 3 Tianjin’s Clinical Research Center for Cancer, Tianjin Medical University Cancer Institute and Hospital, Tianjin, China; 4 Key Laboratory of Cancer Prevention and Therapy, Tianjin, China; 5 Key Laboratory of Breast Cancer Prevention and Therapy, Tianjin Medical University, Ministry of Education, Tianjin, China; 6 Characteristic Medical Center of Chinese People’s Armed Police Force, Tianjin, China; 7 Department of Epidemiology and Biostatistics, Institute of Bioinformatics, University of Georgia, Athens, GA USA

**Keywords:** Cancer, Medical research

## Abstract

Intersectin1 (ITSN1) contains two isoforms: ITSN1-S and ITSN1-L, which is highly regulated by alternative splicing. However, the alteration of alternative splicing and its importance in cancer is still unknown. In this study, our transcriptome analysis by using a large glioma cohort indicated the two isoforms exerted opposite function in glioma progression. Our previous results had shown ITSN1-S could promote glioma development; however, the function of ITSN1-L remained unknown. In this study, we first confirmed that ITSN1-L exerted an inhibitory role in glioma progression both in vivo and in vitro, which was contrary to the function of ITSN1-S. In additional, we also elucidated the mechanisms of ITSN1-L in inhibiting tumor progression. First, we revealed ITSN1-L could interact with α-tubulin to promote HDAC6-dependent deacetylation of ac-tubulin leading to decreased cell motility. Second, ITSN1-L could attenuate cell–substrate adhesion through FAK/integrin β3 pathway. Third, ITSN1-L was able to strengthen cell–cell adhesion by upregulating N-cadherin expression and its re-localization to membrane by ANXA2 and TUBB3/TUBB4. In conclusion, we found for the first time that two isoforms produced by alternative splicing exerted opposite functions in glioma development. Therefore, upregulation of ITSN1-L expression as well as downregulation of ITSN1-S expression probably was a better strategy in glioma treatment. Our present study laid a foundation for the importance of alternative splicing in glioma progression and raised the possibility of controlling glioma development completely at an alternative splicing level to be a more effective strategy.

## Introduction

Alternative splicing plays an important role in many important biological processes, such as tissue differentiation^1,2^. The disorder of alternative splicing can lead to a variety of diseases, including tumors^3,4^. Recent studies found tumor development and metastasis due to alternative splicing in tumor transcriptome; however, alternative splicing and its importance in cancer is still less studied^5,6^. Several reports indicated that alternative splicing was associated with tumor progression, metastasis, drug resistance, and other carcinogenic processes^7–11^. Therefore, understanding the potential function of alternative splicing in tumor progression will probably help us to discover new carcinogenic mechanisms and therapeutic strategies.

Intersectin1 (ITSN1) is a highly conserved scaffold protein during evolution with multiple domains^12^. *ITSN1* gene frequently encodes two major isoforms referred to as long isoform (ITSN1-L) and short isoform (ITSN1-S), which is highly regulated by alternative splicing. The long ITSN1 mRNA is produced by skipping the last exon of the short transcript and utilizing the next available exon, which continues the open reading frame^13^. As a consequence, ITSN1-S contains two EH domains, a coiled-coil domain, and five SH3 domains and is ubiquitously expressed, and ITSN1-L has three additional domains in its C-terminal part: a DH (Dbl homology) domain, a PH (pleckstrin homology) domain, and a C2 domain and is specifically expressed in neurons^14,15^. In addition, the expression of the two isoforms was altered in different cell types. According to our previous results, the two isoforms, ITSN1-L and ITSN1-S, had their own specific cellular distribution in the central nervous system (CNS): ITSN1-L was highly enriched in neurons, whereas ITSN1-S was detected mainly in astrocytes and microglia^16^. These results suggested that the expression of ITSN1-L and ITSN1-S was strictly regulated in different cell types, and their unique cellular distributions should correspond to their function. In this study, according to our transcriptome analysis by a large glioma cohort, we found that the expression of ITSN1-L was negatively correlated with malignancy of glioma, which was different from ITSN1-S. These results predicted that the function of two isoforms may be different in glioma progression. ITSN1-S has been widely studied in glioma progression; however, the function of ITSN1-L in glioma remains unknown^17–20^.

In this study, we found for the first time that two isoforms produced by alternative splicing exerted opposite function in glioma development. We found that ITSN1-L could decrease the aggressiveness phenotype of glioma cells while ITSN1-S could promote glioma progression. Therefore, upregulation of ITSN1-L expression as well as downregulation of ITSN1-S expression probably was a better strategy in glioma treatment. Our present study laid a foundation for the importance of alternative splicing in tumor progression and raised the possibility of controlling tumor development completely at an alternative splicing level to be a more effective strategy.

## Results

### Enrichment analysis of ITSN1-L in The Cancer Genome Atlas (TCGA) glioma dataset

**Fig. 1:**
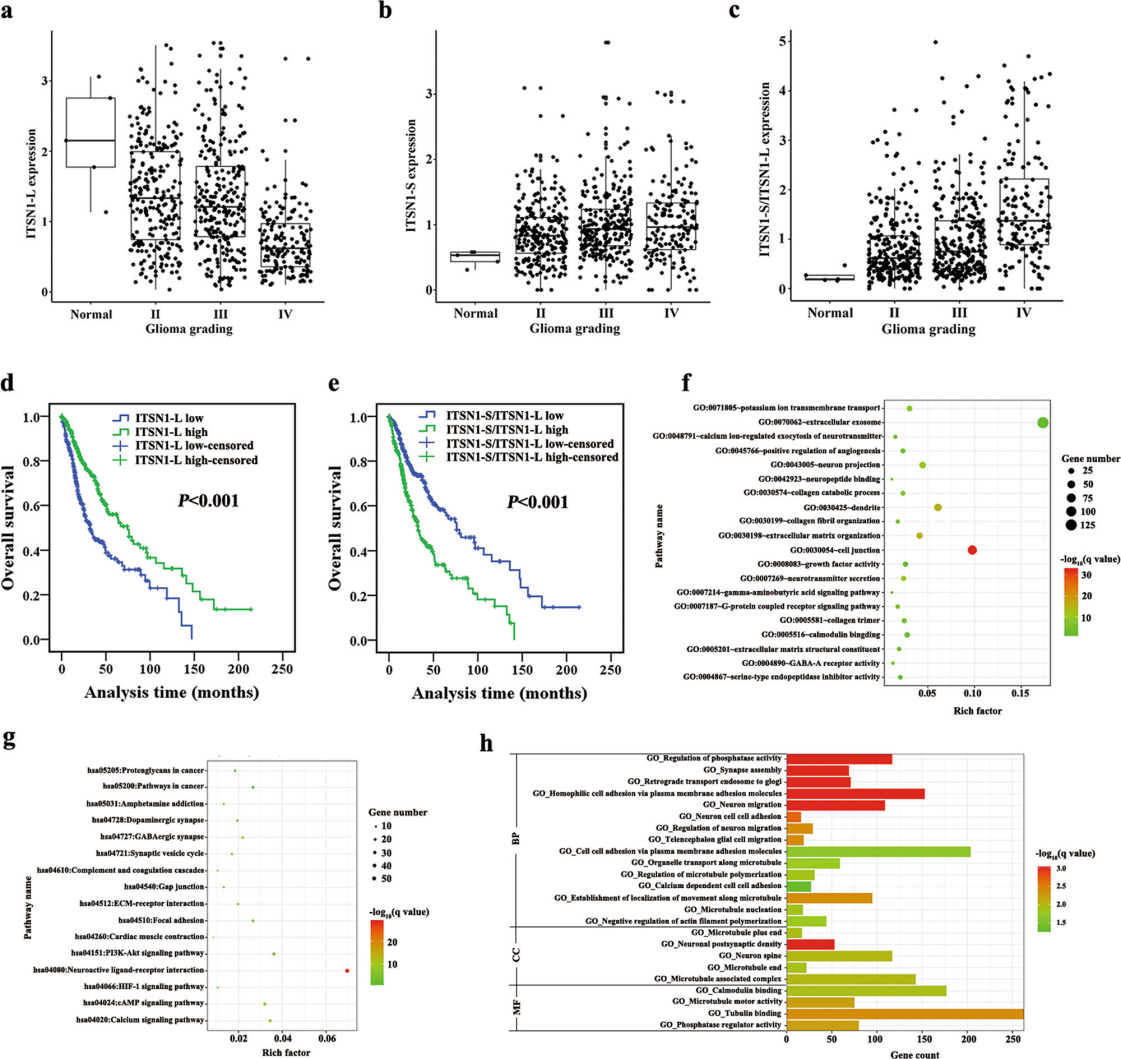
Enrichment analysis of intersectin1 (ITSN1)-L in The Cancer Genome Atlas glioma dataset. **a** The mRNA level of ITSN1-L in normal and glioma tissues. Grade IV vs normal: *P* < 0.001, Grade IV vs Grade II: *P* < 0.001, Grade IV vs Grade III: *P* < 0.001. The *y* axis showed absolute transcript expression levels measured by log_2_ (fragments per kilobase of exon per million fragments mapped (FPKM)). **b** The mRNA level of ITSN1-S in normal and glioma tissues. Grade II vs normal: *P* < 0.001, Grade (IV and III) vs normal: *P* < 0.001, Grade (IV and III) vs Grade II: *P* = 0.002. The *y* axis showed absolute transcript expression levels measured log_2_ (FPKM). **c** The ratio of mRNA of ITSN1-S to ITSN1-L expression in normal and glioma tissues. Grade IV vs normal: *P* < 0.001, Grade IV vs Grade II: *P* < 0.001, Grade IV vs Grade III: *P* = 0.009. (*P* value was calculated using one-way analysis of variance in **a**–**c**, Grade II *N* = 249, Grade III *N* = 265, Grade IV *N* = 159, Normal *N* = 5). **d** Patients with high mRNA ITSN1-L expression exhibited a longer overall survival compared with patients of low mRNA ITSN1-L expression. **e** Kaplan–Meier analysis of survival of glioma patients with the ratio of mRNA of ITSN1-S to ITSN1-L expression. **f**, **g** Gene Ontology (GO) (**f**) and Kyoto Encyclopedia of Genes and Genomes (**g**) pathway analysis of the differential expression genes by DAVID. **h** GO functional annotation genes in the module obtained by gene set enrichment analysis. The *y* axis showed the GO terms and *x* axis showed the −log_10_ (*q* value) of each term. BP biological progress, CC cellular component, MF molecular function

Analysis of TCGA database identified the mRNA expression of two isoforms of ITSN1 in glioma. Figure 1a showed that ITSN1-L mRNA level in glioma was lower than normal tissues and its expression in Grade IV was also lower than Grades II and III. In contrast, the ITSN1-S mRNA level in glioma was higher than in normal tissues (Fig. 1b). In addition, the ratio of mRNA ITSN1-S to ITSN1-L expression increased with glioma histological grade (Fig. 1c). In the following, survival analysis indicated that the patients with higher expression of ITSN1-L had a better prognosis (Fig. 1d) while the patients with higher ratio of mRNA ITSN1-S to ITSN1-L expression exerted a shorter overall survival (Fig. 1e). These findings above suggested that higher ITSN1-L level indicated a better prognosis. Then 1229 differential expression genes (DEGs), which were detected between high and low ITSN1-L expression patients, were enriched by using DAVID database for Gene Ontology functional and Kyoto Encyclopedia of Genes and Genomes pathway enrichment analysis (Fig. 1f, g). We detected the genes mainly enriched in “Focal adhesion,” “Cell junction,” “Collagen catabolic process,” and “Extracellular matrix-receptor interaction.” Furthermore, gene set enrichment analysis (GSEA) was applied and biological processes such as migration and adhesion were found to be enriched in patients with high ITSN1-L expression (Fig. 1h). Therefore, it can be speculated that the function of ITSN1-L in glioma progression may be closely related to these processes.

### ITSN1-L did not affect proliferation in glioma cells

**Fig. 2:**
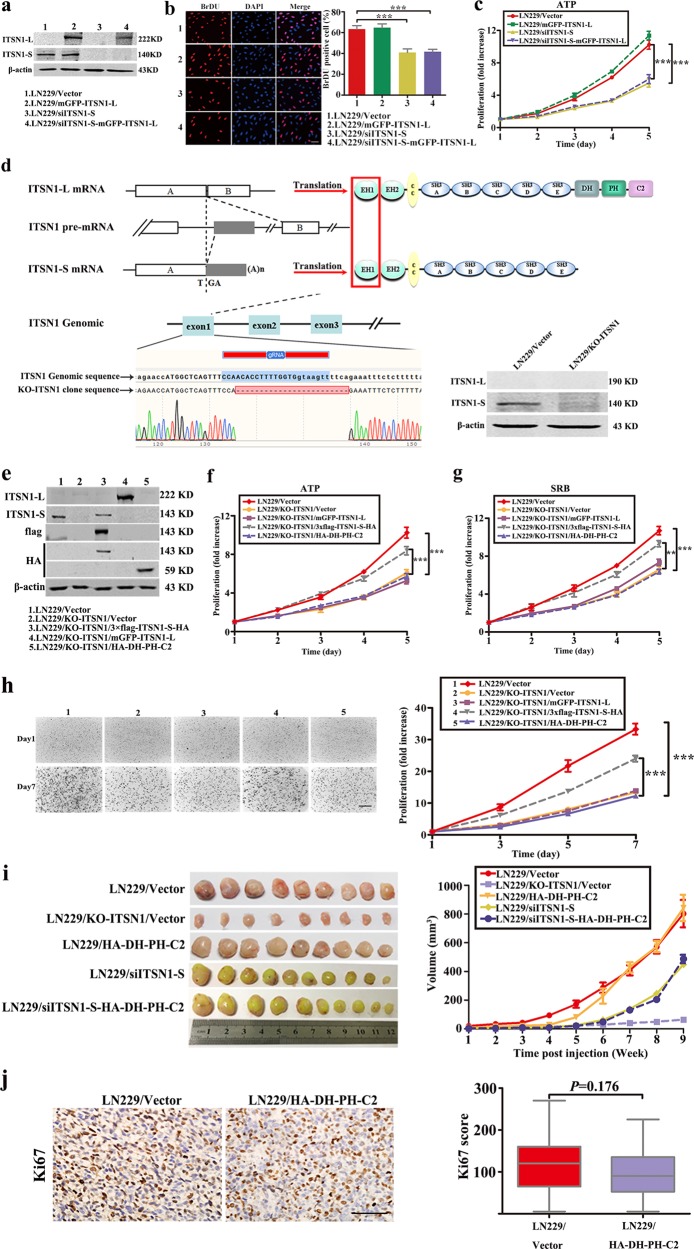
Intersectin1 (ITSN1)-L did not affect proliferation in glioma cells. **a** LN229 cells stably infected with lentivirus containing ITSN1-S shRNA sequences or full-length ITSN1-L were lysed and analyzed by western blot. β-Actin was used as loading control. **b** 5-Bromo-2-deoxyuridine assay was used to detect cell proliferation ability. **c** Proliferation ability was examined by ATP/viability assay. Scale bars, 50 μm. **d** Generation of two isoforms of human *ITSN1* gene knockout LN229 cells. It showed ITSN1 two isoforms generated by alternative splicing of last exon of ITSN1-S. To knockout ITSN1 two isoforms, we designed gRNA in exon1, which is the common region of two isoforms (line 4). Both sequencing and western blot confirmed the knockout of ITSN1 expression. **e** ITSN1 knockout cells stably infected with lentiviruses of vector, 3×flag-ITSN1-S-HA, mGFP-ITSN1-L, and HA-DH-PH-C2, respectively, were lysed and analyzed by western blot to assess protein levels. **f**, **g** Proliferation ability was examined by ATP/viability assay (**f**) and Sulforhodamine B assay (**g**). **h** Three-dimensional proliferation assay was performed in the indicated cells. Colonies were stained with neutral red after different time points of growth and then photographed (×4) and analyzed using the Scion image analysis system. Scale bars, 500 μm. **i** Subcutaneous mouse xenograft models were performed in vivo. Sixty Nu/Nu mice were used and divided into five groups. The size of xenograft tumors was measured each week. The representative images of tumor size of each group were captured after sacrificed. Quantitative results of in vivo assay were analyzed in the right panel. **j** The expression pattern of Ki67 was detected in paraffin section by immunohistochemical analysis. Scale bars, 100 μm. Values were expressed as mean ± SD from three independent experiments (Student’s *t* test, **P < 0.01, ***P < 0.001)

In order to discover how ITSN1-L contributed to glioma development, plasmid with full length of ITSN1-L and RNA interference of ITSN1-S were transfected into LN229 human glioblastoma cells, respectively. Expression of ITSN1-S and ITSN1-L were examined by western blot (Fig. 2a). 5-Bromo-2-deoxyuridine (BrDU) assay and ATP/viability assay showed that reduction of ITSN1-S inhibited cell growth, which further confirmed our previous findings^18^ and ITSN1-L overexpression had little effect on cell growth (Fig. 2b, c). Sulforhodamine B (SRB) assay also indicated similar results (Supplementary Fig. 1a). Then cell clone overexpressing HA tagged DH-PH-C2 domains of ITSN1-L were set up. ATP/viability, SRB, and three-dimensional (3D) collagen cell colony-formation assays were performed. The results showed that upregulation of DH-PH-C2 domains of ITSN1-L did not affect cell proliferation (Supplementary Fig. 1b–e). Furthermore, CRISPR/Cas9 system was used to generate *ITSN1* gene knockout cells (LN229/KO-ITSN1) (Fig. 2d). ATP/viability assay, SRB assay, and 3D collagen cell colony-formation assay results suggested that ITSN1-S, not ITSN1-L, could promote glioma cell proliferation (Fig. 2e–h). In the following, the function of ITSN1-S and ITSN1-L were also confirmed by subcutaneous mouse xenograft models in vivo. Sixty Nu/Nu mice were used and divided into five groups. The sizes of xenograft tumors were measured each week. Xenograft tumors were dissected after 9 weeks and are shown in Fig. 2i. LN229/HA-DH-PH-C2 mice group showed similar tumor volume compared to control, while ITSN1 knockout group showed the smallest tumor volume. The expression pattern of Ki67 was similar with the tumor volume in LN229/HA-DH-PH-C2 mice group and control (Fig. 2j). Overall, these findings further confirmed that the two splice variants of ITSN1 exerted different function in cell proliferation.

### ITSN1-S and ITSN1-L displayed opposite roles in cell migration and invasion

**Fig. 3:**
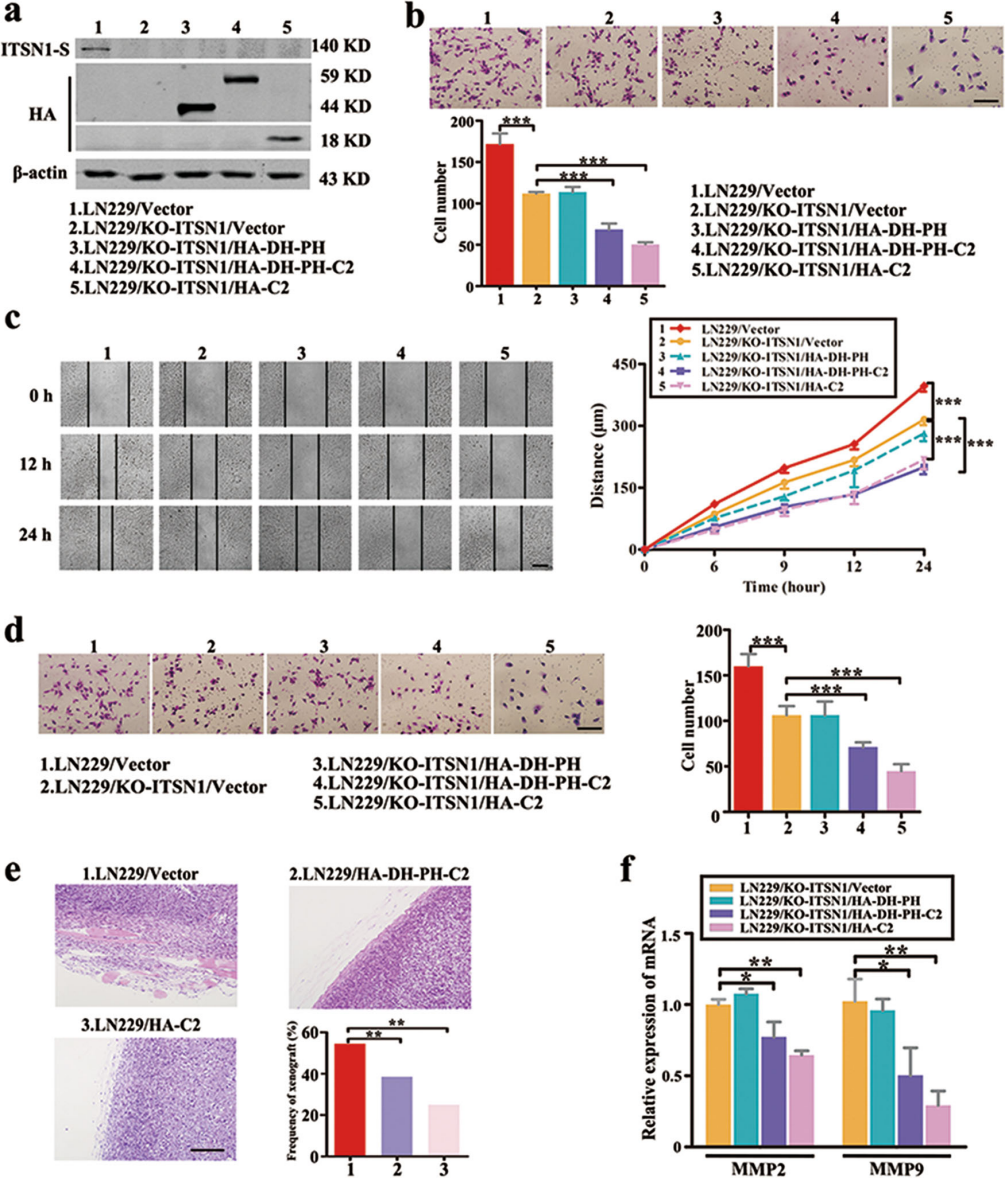
Intersectin1 (ITSN1)-S and ITSN1-L displayed opposite roles in cell migration and invasion. **a** Several exogenous different domain structure fragments of ITSN1-L were transfected into LN229/KO-ITSN1 cells and tested with anti-HA and anti-ITSN1-S antibodies in western blot. **b** Migration assay of the indicated cells. Cells migrating through transwell inserts were stained, photographed (×200), and quantified. **c** Results of scratch assay. The images were photographed at 0, 12, and 24 h (×100). **d** Invasion assay results. Cells invading through extracellular matrix-coated transwell inserts were stained, photographed (×200), and quantified. **e** Representative images of hematoxylin–eosin staining in LN229/Vector, LN229/HA-DH-PH-C2, and LN229/HA-C2 cells (×200). The frequency of xenograft invasion in mice was analyzed quantitatively. **f** Quantitative real-time PCR results of mRNA level of matrix metalloproteinase 2 (MMP2) and MMP9. GAPDH was used as control. Values were expressed as mean ± SD from three independent experiments (Student’s *t* test, *P < 0.05, **P < 0.01, ***P < 0.001). Scale bars, 200 μm

Migration assay and scratch assay showed both ITSN1-S reduction and ITSN1-L overexpression could lead to lower cell migratory capacity than their control groups, respectively, suggesting that ITSN1-L could inhibit cell migration and ITSN1-S exerted opposite function (Supplementary Fig. 2a, b). Invasion assay showed the similar conclusion in Supplementary Fig. 2c. In the following, we designed several different domain structure fragments of ITSN1-L to detect its key functional domains. Results of migration assay, scratch assay, and invasion assay indicated that C2 domain of ITSN1-L, but not DH or PH domain, played the critical role in attenuating migration and invasion (Supplementary Fig. 2d–g). Next, we also applied the above functional experiments in ITSN1 knockout cells and corresponding control cells, which confirmed the similar conclusion (Fig. 3a–d). Based on the above results, we further validated the role of ITSN1-L in cell invasion by in vivo experiments. Hematoxylin–eosin (HE) staining was applied in xenograft paraffin specimens and frequency of xenograft invasion was counted and determined (Fig. 3e and Table 1). In addition, DEGs enrichment analysis showed matrix metalloproteinase 9 (MMP9) or MMP2 expression in ITSN1-L high expression samples was twice lower than that in ITSN1-L low expression samples, and the heatmap is shown in Supplementary Fig. 2h. This intension was also confirmed in Fig. 3f and Supplementary Fig. 2i. Taken together, we concluded that ITSN1-L could inhibit cell migration and invasion, while ITSN1-S exerted the opposite function.

**Table 1:** Frequency of xenograft invasion in mice analyzed quantitatively

Group	Cases	Frequency of xenograft invasion (%)	*χ* ^*2*^	*P* value
LN229/Vector	30	16 (53.3)	10.360	0.006^a^
LN229/HA-DH-PH-C2	28	6 (21.4)		
LN229/HA-C2	20	3 (15.0)		

*P* value was calculated by Chi-square test

*P* (LN229/Vector and LN229/HA-DH-PH-C2) = 0.012^a^; *P* (LN229/Vector and LN229/HA-C2) = 0.015^a^; *P* (LN229/HA-DH-PH-C2 and LN229/HA-C2) = 0.851

^a^Indicates statistical significance

### C2 domain of ITSN1-L interacted with α-tubulin and disturbed stable cytoskeletal microtubules

**Fig. 4:**
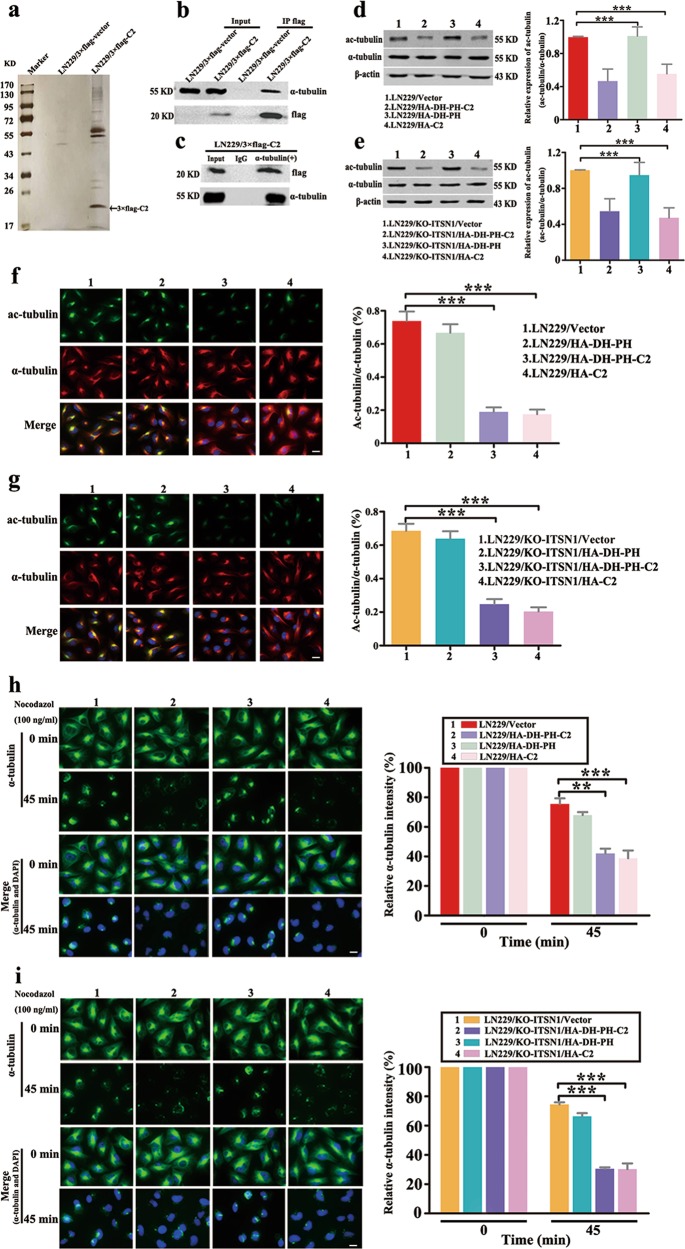
C2 domain of intersectin1 (ITSN1)-L interacted with α-tubulin and disturbed stable cytoskeletal microtubules. **a** Mass spectrometric analysis of 3×flag-C2-associated proteins. Cellular extracts were immunopurified with anti-flag M2 gel and eluted with flag peptides. The eluates were resolved by sodium dodecyl sulfate–polyacrylamide gel electrophoresis, silver-stained, and analyzed by mass spectrometry. **b** Immunoprecipitation (IP) was performed with anti-flag M2 affinity gel followed by immunoblotting with antibodies against flag and α-tubulin. **c** IP was performed by using antibodies anti-α-tubulin or control IgG followed by western blot. **d, e** Expression of ac-tubulin and α-tubulin was determined by western blot analysis in LN229 cells (**d**) and LN229/KO-ITSN1 cells (**e**). The relative ratio of ac-tubulin (ac-tubulin/α-tubulin) was measured by Gray analysis of the western blot data. **f**, **g** Representative immunofluorescent images of ac-tubulin and α-tubulin expression in LN229 cells (**f**) and LN229/KO-ITSN1 cells (**g**). Nuclei were stained with 4,6-diamidino-2-phenylindole. The fluorescence intensities of ac-tubulin and α-tubulin were quantified using the ImageJ software. **h**, **i** Images of microtubule stability in LN229 cells (**h**) and LN229/KO-ITSN1 cells (**i**) by using nocodazole (100 ng/ml) treated for 45 min and stained with anti-α-tubulin antibody. The fluorescence intensity of the remaining microtubules was measured. Values were expressed as mean ± SD from three independent experiments (two-way analysis of variance, **P < 0.01, ***P < 0.001). Scale bars, 20 μm

To gain the mechanisms of ITSN1-L-mediated motility suppression, mass spectrometry was employed to investigate the interaction proteins with C2 domain of ITSN1-L in vivo, and it showed that α-tubulin was one of the interaction proteins (Fig. 4a–c). α-Tubulin is a component of microtubules and ac-tubulin is a widely used marker of stable microtubules^21^. Therefore, we hypothesized whether ITSN1-L could regulate the stability of cytoskeletal microtubules to effect cells’ motility. As shown in Fig. 4d, e, the level of ac-tubulin was attenuated in DH-PH-C2 or C2 domain of ITSN1-L-overexpressing cells. The immunofluorescence staining also showed similar results (Fig. 4f, g). Next, to further verify this phenomenon, we also detected microtubules depolymerization by using nocodazole, a microtubule-destabilizing agent. α-tubulin depolymerized level in C2 domain of ITSN1-L upregulated cells was higher than that in control cells (Fig. 4h, i). Altogether, these results further suggested that ITSN1-L was able to regulate cell migration through inhibiting the stability of cytoskeletal microtubules.

### C2 domain of ITSN1-L promoted microtubule deacetylation through activation of HDAC6

**Fig. 5:**
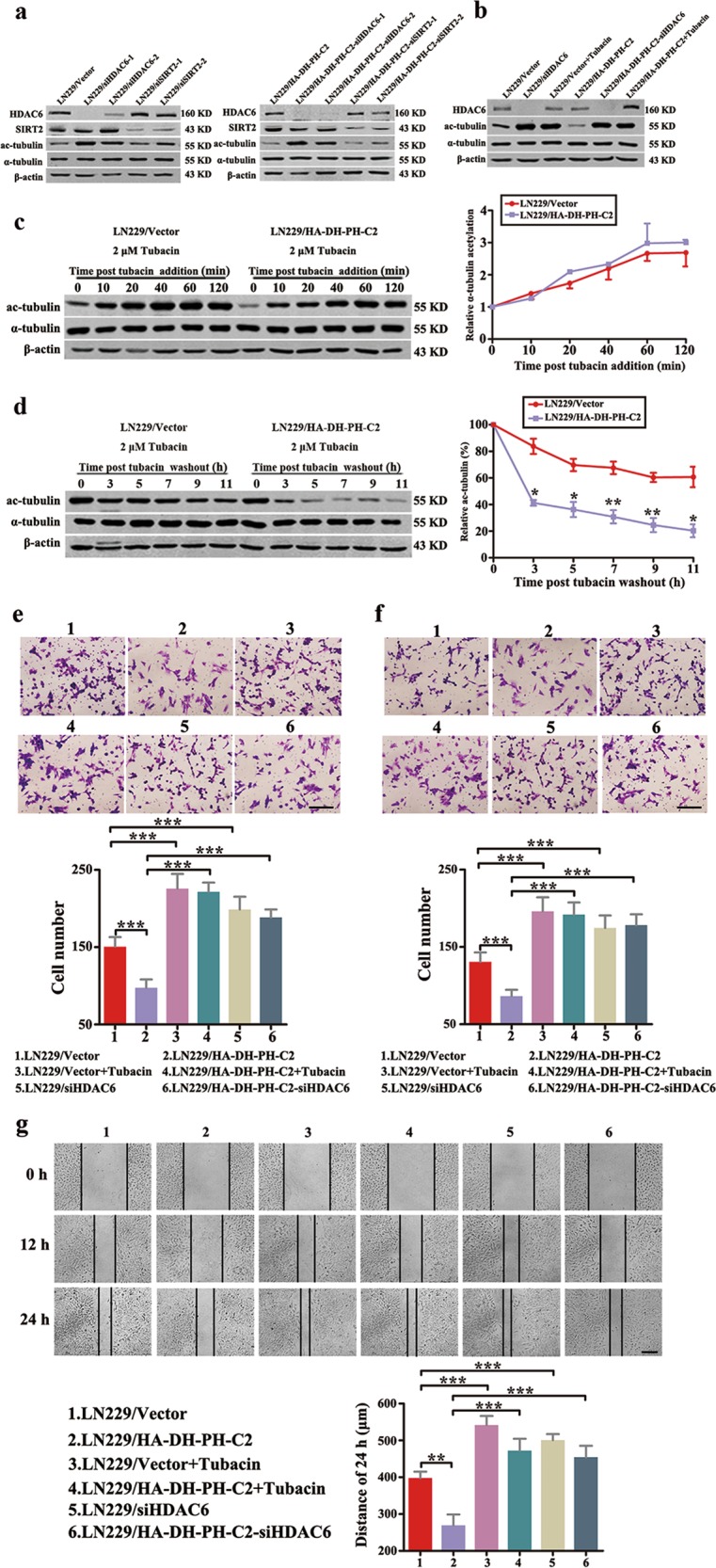
C2 domain of intersectin1 (ITSN1)-L promoted microtubule deacetylation through activation of HDAC6. **a** LN229 cells stably infected with lentivirus containing HDAC6 or SIRT2 shRNA sequences were lysed and analyzed by western blot. **b** Western blot of LN229 cells treated with shHDAC6 or 2 μM tubacin for 4 h. The level of HDAC6, ac-tubulin, and α-tubulin were examined. **c** Western blot (left panel) and quantitation (right panel) of the time course of α-tubulin acetylation after the addition of 2 μM tubacin. **d** Cells were treated with 2 μM tubacin for 12 h. Then tubacin was removed and the rate of α-tubulin deacetylation was tested by western blot (left panel) and quantitation (right panel). **e**–**g** Migration assay (**e**), invasion assay (**f**), and scratch assay (**g**) were performed in LN229 cells treated with shHDAC6 or 2 μM tubacin. Values were expressed as mean ± SD from three independent experiments (Student’s *t* test, *P < 0.05, **P < 0.01, ***P < 0.001). Scale bars, 200 μm

As is well known, ac-tubulin level is tightly controlled through the activity of acetyltransferases and deacetylases (in which HDAC6 and SIRT2 were the main deacetylase members to regulate ac-tubulin level)^22,23^. Therefore, we wondered that C2 domain of ITSN1-L inhibited ac-tubulin through either inhibition of the activity of acetyltransferases or activation of deacetylase. First, we confirmed that HDAC6, but not SIRT2, promoted the α-tubulin deacetylation in LN229 cells (Fig. 5a), which was consistent with previous results^24^. Likewise, similar results also confirmed by using tubacin, which was the specific inhibitor of HDAC6 in Fig. 5b. In the following, we found the acetyltransferase activity was similar in both control and LN229/HA-DH-PH-C2 cells by measuring the rate of α-tubulin acetylation upon HDAC6 restraint with tubacin (Fig. 5c). Meanwhile, HDAC6 activity in DH-PH-C2 domains of ITSN1-L-overexpressing cells was higher than the controls (Fig. 5d). These results revealed that HDAC6 activation rather than acetyltransferases inactivation promoted α-tubulin deacetylation in DH-PH-C2 domain-overexpressing cells. Then migration and invasion assays showed that reduction of HDAC6 or inhibition of HDAC6 activation by tubacin could promote the migration and invasion ability in DH-PH-C2 domain-overexpressing cells (Fig. 5e, f). Likewise, scratch assays also exerted the similar results that are shown in Fig. 5g. Collectively, these data demonstrated that C2 domain of ITSN1-L could inhibit cell motility through activating HDAC6 activity.

### C2 domain of ITSN1-L could reduce cell adherence to substrate and enhance cell–cell adhesion

**Fig. 6:**
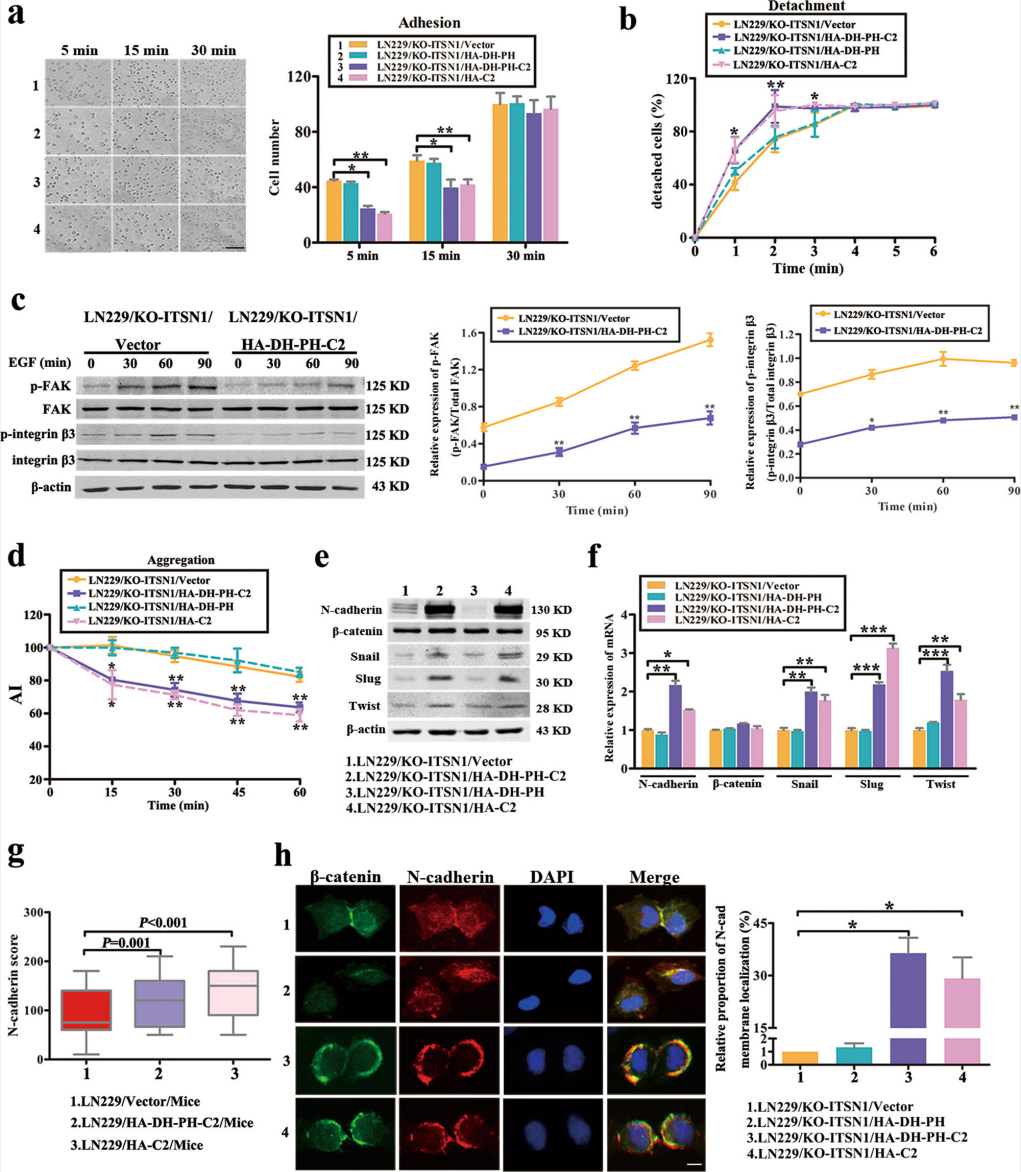
C2 domain of intersectin1 (ITSN1)-L could reduce cell adherence to substrate and enhance cell–cell adhesion. **a** Comparison of the substrate adhesion ability of stable expressed fragments of ITSN1-L in LN229/KO-ITSN1 cells at 5, 15, and 30 min, respectively. Cell number was determined in 5 fields on every coverslip under microscopy (×200). Scale bars, 200 μm. **b** The strength of different cell attachment to substratum was estimated in LN229/KO-ITSN1 cells by the detachment assay. The strength of attachment to substratum was estimated by the rate of detachment after trypsinization. **c** LN229/KO-ITSN1/HA-DH-PH-C2 and control cells were starved for 24 h before epidermal growth factor (100 ng/ml) stimulated for different time points. Phosphorylated focal adhesion kinase (FAK) and phosphorylated integrin β3 in whole cellular lysates from different cells were determined by western blot. Total FAK and integrin β3 were used as loading controls. Each result is representative from at least three independent experiments, and the band intensity ratio of phosphorylated protein vs its loading control is indicated in the right panel. **d** Aggregation assays showing the aggregation index (AI) of the indicated cells. **e**, **f** The expression of N-cadherin, β-catenin, Snail, Slug, and Twist were detected by western blot (**e**) and quantitative real-time PCR (**f**) in different fragments of ITSN1-L-overexpressed cells. **g** The expression pattern of N-cadherin was detected in paraffin section by immunohistochemical analysis. **h** Immunofluorescence assays were used to detect the distribution of N-cadherin and β-catenin in LN229/KO-ITSN1 cells. The nuclei of cells were labeled by 4,6-diamidino-2-phenylindole. Scale bars, 20 μm. Values were expressed as mean ± SD from three independent experiments (Student’s *t* test, *P < 0.05, **P < 0.01, ***P < 0.001)

Adhesion of cells to the growth substrate and cell–cell adhesion were two key processes associated with cell movement^25^. We then examined the adhesion ability of cells to substrate by adhesion and detachment assays. As shown in Fig. 6a, b and Supplementary Fig. 3a, b, adhesion ability to substrate of C2 domain of ITSN1-L-overexpressing cells was significantly impaired compared with controls. It was known that focal adhesion kinase (FAK) is one important scaffold protein binding to the cytoplasmic tail of integrin β to promote cell attachment^26,27^. Thus we examined the epidermal growth factor (EGF)-induced activation of FAK and integrin β3 by western blot in this study and it showed that phosphorylated FAK and phosphorylated integrin β3 levels were severely reduced in ITSN1-L DH-PH-C2 domain-overexpressing cells (Fig. 6c and Supplementary Fig. 3c), which were consistent with the results of Fig. 6a, b and Supplementary Fig. 3a, b.

It has been reported previously that reduction of cell–cell adhesion could enhance cell migration ability. In present study, we detected that the cell–cell adhesion ability of ITSN1-L C2 domain-overexpressing cells was significantly strengthened compared with controls in aggregation assay (Fig. 6d and Supplementary Fig. 3d). Meanwhile, differential genes and heatmap of cell–cell adhesion gene set showed that mRNA of CDH2 gene (which encodes N-cadherin protein) was upregulated in ITSN1-L high expression group compared with control (Supplementary Fig. 3e). We also found the protein and mRNA levels of N-cadherin and its upstream transcription factors (Snail, Slug and Twist) were higher in ITSN1-L C2 domain-overexpressing groups than controls (Fig. 6e, f and Supplementary Fig. 3f, g). In addition, the change of N-cadherin protein level was confirmed in animal experiments by immunohistochemistry (Fig. 6g). Next, the immunofluorescence staining results also showed that overexpression of ITSN1-L C2 domain promoted localization of β-catenin and N-cadherin from cytoplasm to membrane (Fig. 6h and Supplementary Fig. 3h). In summary, we had verified that C2 domain of ITSN1-L was essential for cell adhesion. Meanwhile, ITSN1-L could increase N-cadherin-dependent cell–cell adhesion and attenuate cell–substrate adhesion through FAK/integrin β3 pathway.

### C2 domain of ITSN1-L upregulated N-cadherin expression and translocation by ANXA2 and TUBB3/TUBB4

**Fig. 7:**
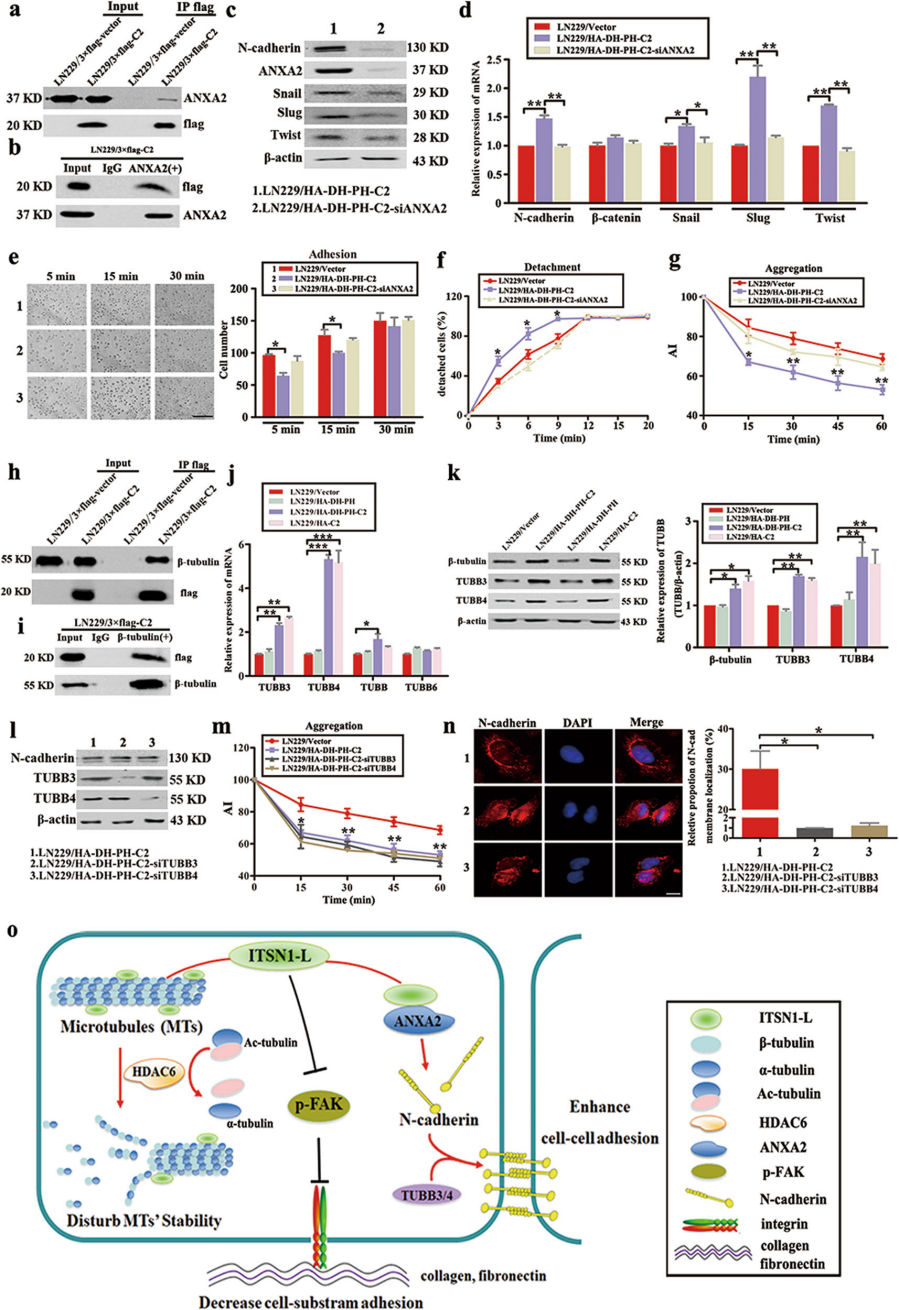
C2 domain of intersectin1 (ITSN1)-L upregulated N-cadherin expression and translocation by ANXA2 and TUBB3/TUBB4. **a** Immunoprecipitation (IP) was performed by using an anti-flag M2 affinity gel. Expression of flag-tagged C2 domain of ITSN1-L and ANXA2 was determined by western blot. **b** IP was performed by using antibodies against ANXA2 or control IgG. Expression of β-tubulin and C2 domain was determined by western blot analysis. **c** ANXA2 reduction cells were lysed and tested the expression of N-cadherin, Snail, Slug, and Twist by western blot. β-Actin was used as a loading control. **d** Total RNAs were prepared and mRNA levels of the indicated genes were examined by quantitative real-time PCR (qRT-PCR). The levels of mRNA were normalized against that of GAPDH. **e** Adhesion ability to substrate of the indicated cells were imaged (left panel) and counted (right panel) at 5, 15, and 30 min, respectively. Scale bars, 200 μm. **f**, **g** Detachment assay (**f**) and aggregation assay (**g**) results of the indicated cells. **h** IP was performed with anti-flag M2 affinity gel followed by immunoblotting with antibodies against β-tubulin and flag. **i** IP was performed by using anti-β-tubulin antibody followed by western blot. **j** qRT-PCR analysis to measure the mRNA expression of TUBBs. **k** Western blot tested the expression of β-tubulin, TUBB3, and TUBB4 in different fragments of ITSN1-L-overexpressing cells. **l** Cells were treated with the indicated siRNA, and the efficiency of knockdown and N-cadherin expression were examined by western blot. β-Actin was loaded as a control. **m** Aggregation assay results of the indicated cells. **n** Immunofluorescence assay was used to test the distribution of N-cadherin after TUBB3 or TUBB4 reduction. Analysis of surface localization of N-cadherin is shown in right panel. Scale bars, 20 μm. Values were expressed as mean ± SD from three independent experiments (Student’s *t* test, *P < 0.05, **P < 0.01, ***P < 0.001). **o** A proposed schematic model of the ITSN1-L function in cell motility

To further identify which potential molecules regulated N-cadherin protein level, candidate proteins were screened using mass spectrometry. We found C2 domain of ITSN1-L could interact with Annexin A2 (ANXA2) and it was confirmed by immunoprecipitation (IP) experiments (Fig. 7a, b). Of important, reduction of ANXA2 resulted in decreased N-cadherin and its upstream regulators in ITSN1-L C2 domain-overexpressing cells both by western blot and quantitative real-time PCR (qRT-PCR; Fig. 7c, d). Meanwhile, knocking down of ANXA2 also led to reduction in cell–cell adhesion and increase in cells’ adherence to substrate in ITSN1-L C2 domain-overexpressing cells (Fig. 7e–g). Previous studies noted that microtubule-dependent transport was essential for N-cadherin translocation and was required for cell–cell contact formation^28^. Our mass spectrometric assays also revealed that β-tubulin could interact with C2 domain of ITSN1-L and it was confirmed in Fig. 7h, i. β-Tubulin consisted of several subunits, such as TUBB, TUBB3, TUBB4, and TUBB6. Thereafter, we found only TUBB3 and TUBB4 were upregulated in C2 domain-overexpressing groups and reduction of TUBB3 or TUBB4 did not affect N-cadherin expression (Fig. 7j–l). Meanwhile, TUBB3 or TUBB4 reduction impaired the cell–cell adhesion ability and led to N-cadherin scatted in cytoplasm in C2 domain-overexpressing cells (Fig. 7m, n). As a consequence, we verified that ANXA2 was essential to regulate N-cadherin level and TUBB3 and TUBB4 were necessary for the translocation of N-cadherin for maintaining the cell–cell adhesion. Finally, we summarized the signaling pathways in Fig. 7o to show our hypothesis of ITSN1-L function in cell motility.

## Discussion

Although there are a lot of alternative splicing changes in tumors, its function has still remained in an early stage. A series of studies had shown that alternative splicing can be used to distinguish different subtypes of tumors^29^ and were associated with clinic stage, prognosis^30^, and tumor resistance^31^. However, the study of function of different subtypes generated by alternative splicing in tumor progression has remained limited. At present, few reports showed that different isoforms exhibited different functions. For example, splicing factor Sam68 can regulate the alternative splicing of Bcl-x and produce two isoforms of Bcl-x (L) and Bcl-x (S). Bcl-x (L) promoted cell growth and proliferation and inhibited programmed cell death, while Bcl-x (S) promoted cell apoptosis^32,33^. These studies have shown that it is important to understand alternative splicing patterns and mechanisms in tumor progression. In the present study, we first demonstrated the existence of ITSN1 alternative splicing in glioma progression of TCGA dataset. An inverse correlation between ITSN1-L mRNA expression and glioma progression was also shown that was in contrast to ITSN1-S. Moreover, our previous results showed that the two isoforms had their unique localization in CNS, ITSN1-L was enriched in neurons, and ITSN1-S could be detected in astrocytes and microglia that have cell division ability^16^. Therefore, these phenomena indicated that the function of two isoforms probably was different and clarifying the specific function of two isoforms will help us to find new mechanisms in glioma malignancy progression.

Our study provided solid evidences of functional differences between two variants; both in vivo and in vitro experiments showed that ITSN1-L had little effect on cell proliferation but could inhibit glioma cells migration and invasion, while ITSN1-S exerted opposite function. In additional, we also elucidated the detailed mechanisms of ITSN1-L inhibiting glioma progression. First, enrichment analysis showed that the function of ITSN1-L was strongly correlated with focal adhesion and migration. Moreover, solid evidences proved that ITSN1-L decreased cell–substrate adhesion by attenuating the p-FAK level. In fact, focal adhesion consists of microtubule cytoarchitecture, cell–extracellular components, and integrin^34^. It is well known that microtubule acetylation is essential for a variety of fundamental cellular functions, including the migration of diverse cell types^35^. In this study, we demonstrated that ITSN1-L could decrease microtubule acetylation by enhancing HDAC6 activity. Furthermore, it is of note that FAK-mediated activation of RhoA has been shown to stimulate microtubule acetylation^36^, consistent with the result of HDAC6 inhibition. Taken together, these findings suggested that these signaling pathways were probably integrated by ITSN1-L to coordinate the regulation of cell motility.

Reduced intercellular adhesion followed by cell invasion is an essential step in the progression from local malignancy to metastatic disease^37^. Cadherins are highly conserved transmembrane receptors that mediate cell–cell adhesion through their extracellular domain. Consistent with this, our studies showed upregulation of N-cadherin with increased cell–cell adhesion in ITSN1-L-overexpressing glioma cells. Meanwhile, we found that ITSN1-L overexpression promoted N-cadherin/β-catenin complex localization on membrane but did not affect the total level of β-catenin. As reported, β-catenin could translocate to nucleus to activate Wnt target genes, leading to glioma development^38^. Thus we speculated whether ITSN1-L overexpression could affect β-catenin subcellular location to inhibit the malignancy of glioma cells. However, the hypothesis needed to be further verified. Integrin-mediated cell–substrate adhesion and cadherin-mediated cell–cell adhesion often occur at the same time. It has been reported that these two different adhesion modes coordinately regulate the migration of tumor cells^39^. Recent study has shown an inverse correlation between N-cadherin level and glioma invasiveness^40–42^. Moreover, N-cadherin expression could affect the localization, number, and size of focal adhesions, promoting decreased turnover of focal adhesion and attenuated migration^40^. Therefore, it is reasonable to suppose that upregulated N-cadherin probably affects the turnover of focal adhesion to decrease cell–substrate adhesion. Therefore, whatever the mechanism was employed by glioma cells, the strength of cell–cell adhesion caused by increased N-cadherin expression and the impaired cell–substrate adhesion by decreased p-FAK/p-integrin β3 could attenuate the migration and invasion in ITSN1-L-overexpressing glioma cells.

Owing to the expression ratio of ITSN1-S to ITSN1-L being different between glioma and normal tissues, the tumor tissues were more inclined to express ITSN1-S and inhibit the expression of ITSN1-L subtype, which was beneficial to the malignant progression of glioma. Therefore, the two isoforms together regulated the malignant progression of glioma, rather than ITSN1-S alone. This may partly explain why using single protein as treatment target is inefficient in partial patients. The efforts to discover the mechanisms of ITSN1 isoform-specific function will provide novel insights to detect more effective therapeutic strategies for glioma treatment. Thus preventing glioma progression by controlling ITSN1-S expression only would not be the most effective method. Regulating the ratio of the two isoforms by alternative splicing leading to a low ratio of ITSN1-S to ITSN1-L expression should be more effective in glioma treatment. At present, the opposite function of ITSN1 two isoforms was only studied in the progression of glioma; whether it is similar or not in other tumors needs to be verified. Until now, our research provided an experimental support for this hypothesis, and we will look for alternative splicing regulatory mechanisms in the near future.

## Materials and methods

### Cell culture and reagents

LN229 glioblastoma cells were cultured in Dulbecco’s modified Eagle’s medium (DMEM) medium supplemented with 10% fetal bovine serum (FBS) in a 5% CO_2_ incubator at 37 °C. Cells were tested and authenticated in Beijing Microread Genetics Co., Ltd. (Beijing, China) by short tandem repeat profiling. Polyclonal anti-human ITSN1-S antibodies were raised in rabbits as previously described^19^.

### Plasmid construction and transfection

Clone of *Homo sapiens* of full-length ITSN1-S was obtained from Gene Copoeia Inc. (CA, USA). ITSN1-L was provided by Youbio Inc. (Hunan, China). 3×flag-ITSN1-S-HA or GFP-ITSN1-L was constructed via PCR with related primers. HA and 3×flag-labeled fragments of ITSN1-L, including DH-PH-C2 (aa 1237–1679), DH-PH (aa 1462–1679), and C2 (aa 1583–1679), were inserted into pCDH-CMV-MCS-EF1-Puro lentiviral vector. Specific shRNA and scrambled sequence (details shown in Supplementary Table 1) were synthesized and cloned into pLKO.1 vector, respectively. The plasmids were next co-transfected into HEK-293T cells with the packing plasmids ΔR and pVSVg to produce lentivirus. Stable lentivirus-infected cells were selected with puromycin and verified by western blot analysis. Control siRNA, siTUBB3 (5′-gcggaucagcgucuacuacaa-3′), and siTUBB4(5′-gaggccacaggaggaaauuau-3′) were synthesized at GenePharma Inc. (Shanghai, China). The siRNA oligonucleotides were transfected into cells using lipofectamine 2000 (Invitrogen, New York, USA) as per the manufacturer’s recommendations. Interference efficiency of siRNA was routinely assessed by western blot at 48 h after transfection.

### CRISPR/Cas9 mediated *ITSN1* gene knockout

The optimized gRNA (targeting *ITSN1* gene) construct and the Cas9 expression construct, pGK1.1-gRNA-Cas9-puro, were obtained from Genloci Biotechnologies Inc. (Nanjing, China). The gRNA sequence targeting the exon 1 of ITSN1 was: 5′-CCAACACCTTTTGGTGGTAAGTT-3′. Transfection was performed with X-treme GENE HP DNA Transfection Reagent (Roche, France) according to the manufacturer’s instructions. Puromycin-resistant single cell-derived colonies were analyzed by western blot and DNA sequencing to confirm ITSN1 knockout.

### Western blot analysis

Briefly, the cellular lysates were prepared in 1× sodium dodecyl sulfate (SDS) lysis buffer and then were resolved by SDS–polyacrylamide gel electrophoresis (PAGE) and transferred onto nitrocellulose membranes (Millipore, Billerica, MA, USA). The membranes were incubated overnight at 4 °C with the appropriate primary antibody and were then treated with secondary antibodies (Li-Cor Biosciences, Lincoln, NE, USA). Infrared signals were examined by using the Odyssey imaging system (Li-Cor Biosciences, Lincoln, NE, USA).

### BrDU (5-bromo-2-deoxyuridine) assay

Cells were cultured in 6-well plates at 37 °C in a CO_2_ incubator. BrDU 500 μM (Sigma, St. Louis, MO, USA) was added into each well, it can be incorporated into S-phase cells. After 6 h, cells were fixed with 4% paraformaldehyde and incorporated BrDU was detected and quantified. Cell nuclei were stained with 4,6-diamidino-2-phenylindole (DAPI).

### Cell ATP/viability assay and SRB assay

Cells (1 × 10^5^ cells/well) were plated in 24-well plates with 4 replicates for 5 days. Using the CellTiter-Glo Luminescent Cell Viability Assay Kit (Promega, Madison, WI, USA), the ATP levels were measured as per the manufacturer’s description. For the SRB (Sigma, St. Louis, MO, USA) assay, cells were fixed with 10% trichloroacetic acid, then were washed and stained with SRB (0.4%). Tris-base (10 mM) was added to dissolve the SRB and absorbance was measured with microplate reader at 546 nm.

### Three-dimensional proliferation

Collagen A, B, and C from Primaster (Osaka, Japan) were mixed together on ice in a ratio 8:1:1. The collagen solution was added to the prepared cell suspension. The collagen-cell mixture was placed in 6-well plates at 30 μl/drop and placed in 37 °C incubator for 45 min. After gelation, DMEM (3 ml) medium containing 10% FBS were added to each well and incubated in a CO_2_ incubator for multiple time points. At the end of the incubation, neutral red (30 μl) was added to each well for staining. Each collagen droplet was fixed with 10% neutral formalin, washed in water, air dried, and images were taken under a light microscope (Olympus, Tokyo, Japan) at ×4 and analyzed using the Scion image analysis system to observe the proliferation of tumor cells.

### Scratch assay

In all, 7 × 10^5^ cells were plated in 35 mm dishes to form a monolayer overnight. Then the monolayer was lined out with an even trace in the middle using a 10 μl pipette tip. After washing with phosphate-buffered saline (PBS), the cells were cultured in DMEM containing 2% FBS for 24 h at 37 °C incubator, and the wounds were photographed at intervals. The distance of the wounds was measured by the Photoshop software.

### Migration and invasion assay

The migration and invasion capacities of the cells were analyzed using 24-well transwell chambers (Corning, NY, USA) with polycarbonate membranes (8-μm pore size). Cells (1 × 10^4^) in serum-free medium containing 0.1% bovine serum albumin (BSA) were added to the upper chamber, and the lower chamber was filled with medium supplemented with 0.1% BSA and EGF (50 ng/ml) (Peprotech, NJ, USA). Following 24 h of incubation, cells on the upper chamber were completely scraped, and the cells on the lower surface of the membrane were fixed and stained with Giemsa solution and photographed under a microscope (Olympus, Tokyo, Japan) at ×200 magnification. For invasion assay, transwell was coated with prediluted extracellular matrix (3.2 mg/ml) (BD, NJ, USA) for 1 h before cells were added to the upper chamber. The following steps were the same as migration assay.

### IP, silver staining, and mass spectrometry

Cellular extracts were obtained in IP lysis buffer at 4 °C overnight followed by centrifuging at 12,000 × *g* for 30 min. The protein supernatant was incubated with anti-flag M2 gel for 4 h at 4 °C. After washing with Hepes lysis buffer four times, flag peptides were applied to elute the protein complex from the gel. The eluted proteins were resolved by SDS-PAGE, silver stained, and subjected to liquid chromatography tandem mass spectrometry sequencing provided by the Beijing Genomics Institute (Beijing, China).

### Co-immunoprecipitation (CO-IP)

The CO-IP was performed essentially the same as previously described^43^. Briefly, cellular lysates were obtained in CO-IP lysis buffer and gently rotated at 4 °C overnight followed by centrifuging at 12,000 × *g* for 10 min. The supernatant was immunopurified with anti-flag M2 affinity gel and eluted with flag peptides. Western blot was used to examine the expression of α-tubulin, β-tubulin, and ANXA2 (Figs. 4b and 7a, h).

Cell lysates were gently rotated at 4 °C overnight followed by centrifuging. Protein A was added to remove nonspecific protein, the mixture was centrifuged, and the supernatant was divided into two groups by using antibodies or control IgG. Finally, the precipitates were subjected to western blot to examine the expression of target protein (Figs. 4c and 7b, i).

### Immunofluorescence analysis

Briefly, cells were grown on fibronectin-coated glass coverslips. After 24 h, cells were fixed with 4% paraformaldehyde and permeabilized with 0.2% Triton X-100. Primary antibody was used at 4 °C overnight and the fluorescent-conjugated secondary antibodies (Invitrogen, New York, USA) were used at room temperature for 1 h in dark. Cell nuclei were stained with DAPI (Solarbio, Beijing, China), and the cells were examined by a fluorescence microscope (Olympus, Tokyo, Japan). The fluorescence intensity was measured by the ImageJ software.

### Microtubule stability assay

Cells were treated with 100 ng/ml of nocodazole (Sigma, St. Louis, MO, USA) for 45 min to induce microtubule depolymerization. The cells were then washed with PEMT buffer (100 mM PIPES, 1 mM EGTA, 2 mM MgCl_2_, and 0.1% TritonX-100, pH 6.8), and the residual microtubules were examined using immunofluorescence staining and microscopy. The fluorescence intensity was measured by the ImageJ software.

### Immunohistochemistry staining

Immunohistochemistry for N-cadherin (Santa Cruz, California, USA) and Ki67 (Zymed, California, USA) were performed using standard techniques by Streptavidin-Peroxidase (S-P) method. Sections were incubated with primary antibody overnight at 4 °C and then were incubated with secondary antibody. The enzyme substrate was 3, 3-diaminobenzidine tetrahydrochloride (DAB).

### RNA extraction and qRT-PCR

Total RNA was isolated from cells using Trizol reagent (Invitrogen, Carlsbad, CA, USA) according to the manufacturer’s instructions. cDNA was generated by the RTase M-MLV (Takara, Shiga-ken, Japan) as described in the manufacturer’s protocol. Quantitation of all gene transcripts was done by qRT-PCR using SYBR Green PCR Master Mix (TaKaRa, Shiga-ken, Japan), and the expression of GAPDH was used as the internal control. The primer pairs used are shown in Supplementary Table 2; fold changes were calculated using the ΔΔCt method in Microsoft Excel.

### Adhesion assay

The adhesion assays were performed as previously described^44^. Cells (2.5 × 10^5^ cells/ml) were suspended in complete medium and incubated at 37 °C for 20 min. Then 1 ml of cell solution was plated in 12-well plates containing fibronectin-coated glass coverslips. After incubation at different intervals, the cells were washed with cold PBS and fixed with 4% paraformaldehyde. The cells attached to the coverslips were counted under a light microscope (Olympus, Tokyo, Japan) at ×200.

### Detachment assay

The detachment assay was carried out as previously described^44^. Cells (1 × 10^5^ cells/well) were plated in 24-well plates, which had been pretreated with 1.2 mg/ml of matrigel. After 48 h, cells were washed with PBS and then trypsinized with 0.25% trypsin at 20 °C gently. The number of detached cells was determined at various time points, and the total number of cells/well was determined after complete trypsinization.

### Aggregation assay

Cell–cell adhesion ability was detected by an aggregation assay, which was carried out as described previously^44^. After centrifugation, cells were resuspended in DMEM (serum-free medium). Single-cell suspension (5 × 10^5^ cells/ml) were plated in 1% BSA-coated 12-well plates and incubated at 37 °C incubator for different time periods. The cells and cell clusters were counted using a hemocytometer. Aggregation index (AI) = (Number of single cells + Number of cell clusters)/Total number of cells initially added.

### Tumor proliferation and invasion in xenografts

Male athymic Nu/Nu mice (4–6 weeks of age) were purchased from Model Animal Research Center of Nanjing University (Nanjing, China). A total of 3 × 10^6^ cells were subcutaneously inoculated into each mouse. The volume of the tumors was calculated every week. For invasion, 9 weeks after glioblastoma cell subcutaneous injection, the mice were sacrificed and the tumor were removed and fixed with 4% paraformaldehyde. Samples were soaked in wax and then were cut to 5-mm-thick sections with routine histological methods. HE staining was performed on the transplanted tumors to observe the invasion of the transplanted tumors.

### Bioinformatics analysis

The Glioma dataset including patient’s clinical information and processed RNA-sequencing data were downloaded from the database of TCGA (http://gdac.broadinstitute.org). The data contained 678 glioma samples (Grade II *N* = 249, Grade III *N* = 265, Grade IV *N* = 159) samples and 5 normal tissue samples with complete survival information. The mRNA expression of the specific variant was the mean of several transcript fragments per kilobase of exon per million fragments mapped levels. The total value of mRNA expression levels from specific variant was used to compare the difference in ITSN1-L, ITSN1-S, and the ratio of ITSN1-S to ITSN1-L expression between different Grade and normal by one-way analysis of variance (ANOVA) and to acquire the prognostic significance of specific variant mRNA expression in glioma patients by Kaplan–Meier survival analysis. The scan cut-off mode based on median ITSN1 expression was selected by Kaplan–Meier analysis.

Glioma samples from RNA-seq data were divided into two groups according to the median values of the expression of ITSN1-L gene (high vs low expression). The aberrantly expressed mRNAs were analyzed by the R software. DEGs were screened by |log_2_ (Fold Change)| > 2 and *P* < 0.05. The functional annotation tool of GSEA and DAVID Bioinformatics Resources 6.8 were used to verify the remarkable enrichment of gene sets and pathways in the resulting dataset.

### Statistical analysis

The SPSS 19.0 software package (SPSS, Chicago, IL, USA) was used for statistical analysis. One-way ANOVA was used to compare means of multiple experimental groups. When comparing the means of two different groups, two-sided Student’s *t* test was applied. Chi-square test was used for the in vivo analysis. *P* < 0.05 was considered statistically significant in all analyses.

## Supplementary information

Supplementary figure1

Supplementary figure2

Supplementary figure3

Supplementary figure legends

Supplementary Table1

Supplementary Table2

Full gel images
