# Supplementary material for: Alternative splicing-derived intersectin1-L and intersectin1-S exert opposite function in glioma progression
Source: Cell Death Dis. 2019 Jun 3;10(6):431. doi: 10.1038/s41419-019-1668-0 (PMC6547669; doi:10.1038/s41419-019-1668-0)
Supplement: Supplementary file 4 — Supplementary figure legends [file 41419_2019_1668_MOESM4_ESM.doc]

**Supplementary Fig. 1: ITSN1-L did not affect proliferation in glioma cells.**

**a** SRB assay results. **b** LN229 cells stably infected with lentivirus containing ITSN1-S shRNA sequences or HA-tagged fragments of ITSN1-L (DH-PH-C2) were lysed and analyzed by Western blot. β-actin was used as loading control. **c,d** Proliferation ability was examined by ATP/viability assay (c) and SRB assay (d). **e** Three-dimensional proliferation assay was performed. Colonies were stained with neutral red after different time points of growth and then photographed (×4) and analyzed using the Scion image analysis system. Scale bars，500 μm. Values were expressed as mean±SD from three independent experiments (Student’s t-test, ^***^*P*<0.001).

**Supplementary Fig. 2:** **ITSN1-S and ITSN1-L displayed opposite roles in cell migration and invasion.**

**a** Migration assay of indicated cells. Cells migrating through transwell inserts were stained, photographed (×200) and quantified. **b** Results of scratch assay. The images were photographed at 0, 12 and 24 h (×100). **c** Invasion assay results. Cells invading through ECM-coated transwell inserts were stained, photographed (×200) and quantified. **d** Several exogenous different domain structure fragments of ITSN1-L were transfected into LN229 cells and tested by antibody against HA in Western blot. **e,f,g** Migration assay (e), scratch assay (f) and invasion assay (g) results of indicated cells. **h** The heatmap showed the invasion related genes differential expression between ITSN1-L high expression samples and ITSN1-L low expression samples. **i** qRT-PCR results of mRNA level of MMP2 and MMP9. GAPDH was used as control. Values were expressed as mean±SD from three independent experiments (Student’s t-test, ^**^*P*< 0.01, ^***^*P*< 0.001). Scale bars, 200 μm.

Supplementary Fig. 3: C2 domain of ITSN1-L promoted microtubule deacetylation through activation of HDAC6.

a Comparison of the substrate adhesion ability of stable expressed fragments of ITSN1-L in LN229 cells at 5, 15 and 30 min, respectively. Cell numbers were determined in 5 fields on every coverslip under microscopy (×200). Scale bars，200 μm. **b** The strength of different cell attachment to substratum was estimated LN229 cells by the detachment assay. The strength of attachment to substrate was estimated by the rate of detachment after trypsinization. **c** LN229/HA-DH-PH-C2 and control cells were starved for 24 h before EGF (100 ng/ml) stimulated for different time points. Phosphorylated FAK and phosphorylated integrin β3 in whole cellular lysates from the different cells were determined by Western blot. Total FAK and integrin β3 were used as loading controls. Each result is representative from at least three independent experiments, and the band intensity ratio of phosphorylated protein to its loading control is indicated in the right panel. **d** Aggregation assays showing the aggregation index (AI) of indicated cells. **e** The heatmap showed the cell-cell adhesion related genes differential expression between ITSN1-L high expression samples and ITSN1-L low expression samples. **f** qRT-PCR results of mRNA level of the indicated cells. GAPDH was used as control. **g** The expression of N-cadherin, β-catenin, Snail, Slug and Twist were detected by Western blot in different fragments of ITSN1-L overexpressed cells. β-actin was used as a loading control. **h** Immunofluorescence assays were used to detect the distribution of N-cadherin and β-catenin in LN229 cells. The nuclei of cells were labeled by DAPI. Scale bars，20 μm. Values were expressed as mean±SD from three independent experiments (Student’s t-test, ^*^*P*<0.05, ^**^*P*<0.01, ^***^*P*<0.001).
