# Supplementary material for: Alternative splicing-derived intersectin1-L and intersectin1-S exert opposite function in glioma progression
Source: Cell Death Dis. 2019 Jun 3;10(6):431. doi: 10.1038/s41419-019-1668-0 (PMC6547669; doi:10.1038/s41419-019-1668-0)
Supplement: Supplementary file 5 — Supplementary Table1 [file 41419_2019_1668_MOESM5_ESM.doc]

**Supplementary Table 1. ShRNA sequence specific targeting unique protein.**

| **Gene** | **shRNA sequence** |
| --- | --- |
| **ITSN1-S** | **5′-GATCCgcagaggagttcagtatctctCTCGAGagagatactgaactcctctgcTTTTTG-3′** |
| **ANXA2** | **5′-GATCCcgggatgctttgaacattgaaCTCGAGttcaatgttcaaagcatcccgTTTTTG-3′** |
| **HDAC6#1** | **5′-GATCCgtcacttcgaagcgaaatattCTCGAGaatatttcgcttcgaagtgacTTTTTG-3′** |
| **HDAC6#2** | **5′-GATCCgacaacatggaggaggacaCTCGAGtgtcctcctccatgttgtcTTTTTG-3′** |
| **SIRT2#1** | **5′-GATCCctaagctggatgaaagagaCTCGAGtctctttcatccagcttagTTTTTG-3′** |
| **SIRT2#2** | **5′-GATCCcctagaggccaaggcttaaCTCGAGttaagccttggcctctaggTTTTTG-3′** |
| **Scramble** | **5′-GATCCgttctccgaacgtgtcacgtCTCGAGacgtgacacgttcggagaacTTTTTG-3′** |
