# Supplementary material for: Alternative splicing-derived intersectin1-L and intersectin1-S exert opposite function in glioma progression
Source: Cell Death Dis. 2019 Jun 3;10(6):431. doi: 10.1038/s41419-019-1668-0 (PMC6547669; doi:10.1038/s41419-019-1668-0)
Supplement: Supplementary file 6 — Supplementary Table2 [file 41419_2019_1668_MOESM6_ESM.doc]

**Supplementary Table 2. Primer pairs were used in Quantitative real-time PCR.**

| **Target** | **Primer-F** | **Primer-R** |
| --- | --- | --- |
| **MMP2** | **cctgggcagattccaaacct** | **gtacacgcgagtgaaggtga** |
| **MMP9** | **gcaatgctgatgggaaaccc** | **agaagccgaagagcttgtcc** |
| **N-cadherin** | **tgacaatgaccccacgctc** | **gtcctgctcaccactac** |
| **Snail** | **gcgagctcaggactctaat** | **ggacagagtcccagatgagc** |
| **Slug** | **tcatctttgggcgagtgag** | **tgcagctgcttatgtttggc** |
| **Twist** | **gccggagacctagatgtcatt** | **cccacgccctgtttctttga** |
| **β-catenin** | **tacctcccaagtcctgtatgag** | **tgagcagcatcaaactgtgtag** |
| **TUBB3** | **accccggactcccttgaaca** | **tctcaagaagctctccagtttgg** |
| **TUBB4** | **accttccttcacccctgact** | **agagggtgaaagagaagttgga** |
| **TUBB** | **atccagagcagggaaagctg** | **ctcaggccgttgttctaggg** |
| **TUBB6** | **cgtccgcagagccagttc** | **gctctggaacagatagcgca** |
