# Supplementary material for: Alternative splicing-derived intersectin1-L and intersectin1-S exert opposite function in glioma progression
Source: Cell Death Dis. 2019 Jun 3;10(6):431. doi: 10.1038/s41419-019-1668-0 (PMC6547669; doi:10.1038/s41419-019-1668-0)

Full unedited gel for Figure 2a

ITSN1-L

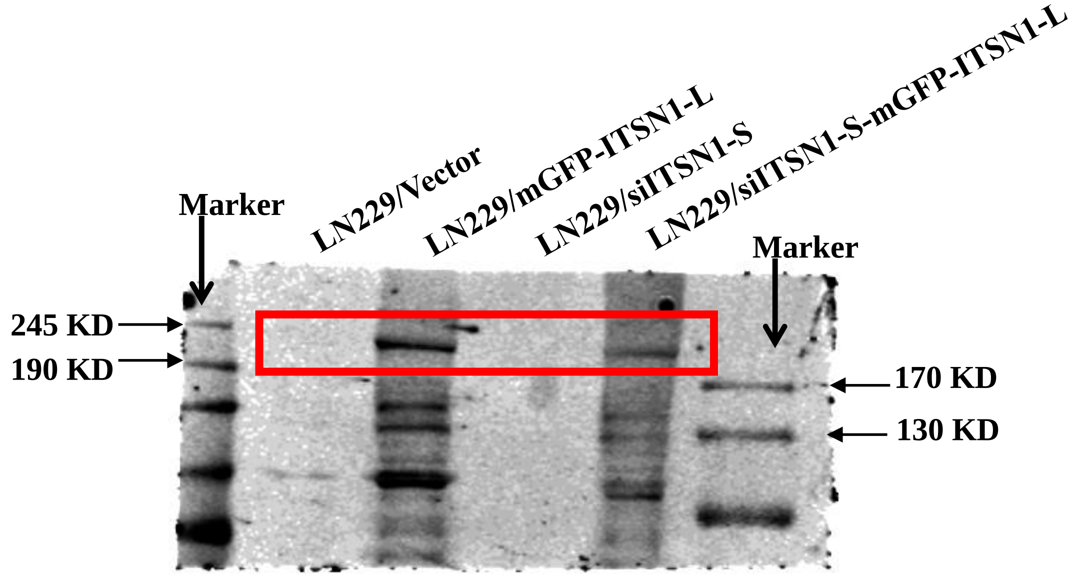

Full unedited gel for Figure 2a

ITSN1-S

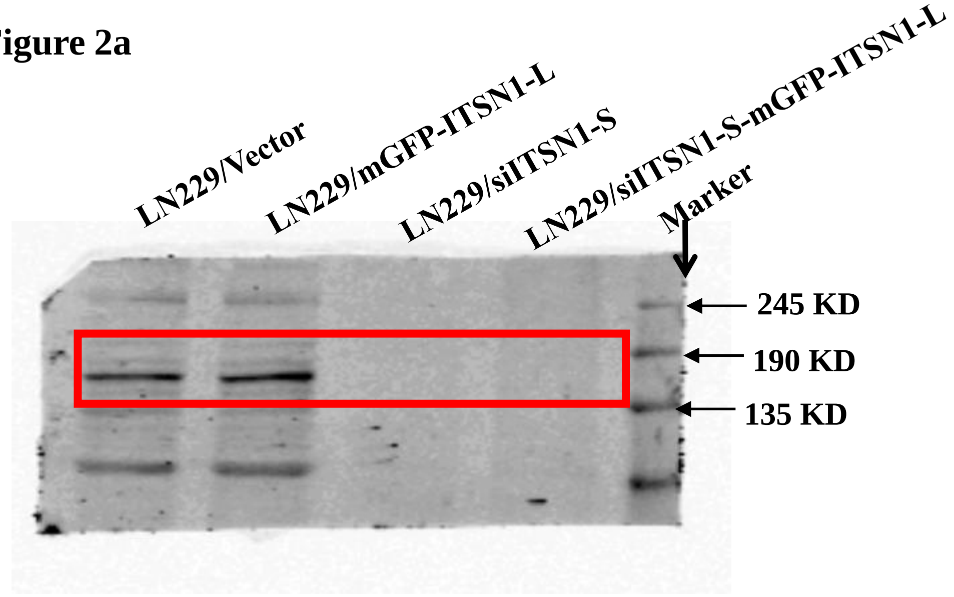

Full unedited gel for Figure 2a

β-actin

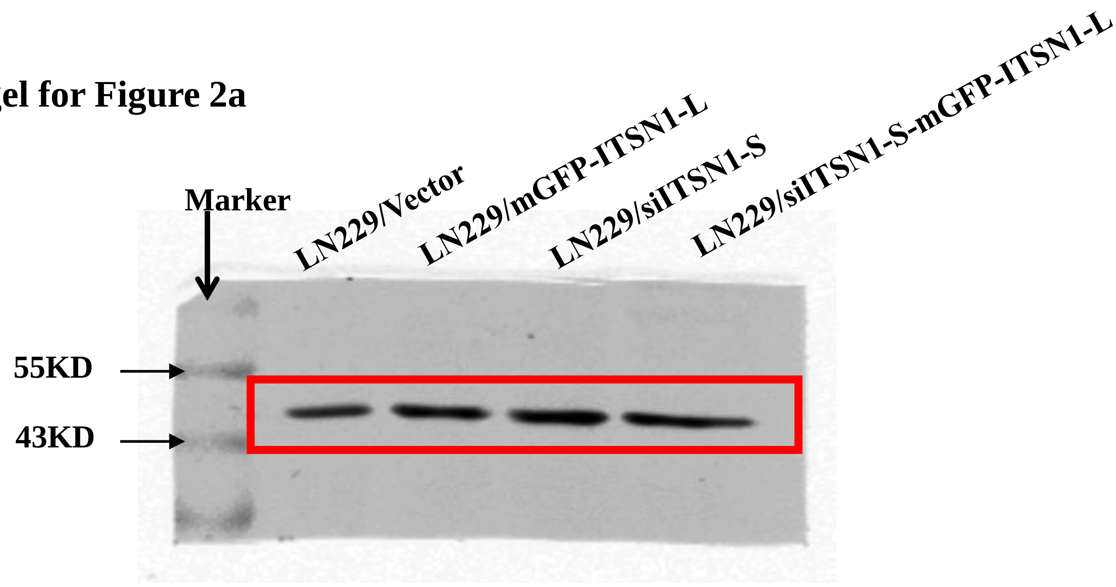

Full unedited gel for Figure 2d

ITSN1-L

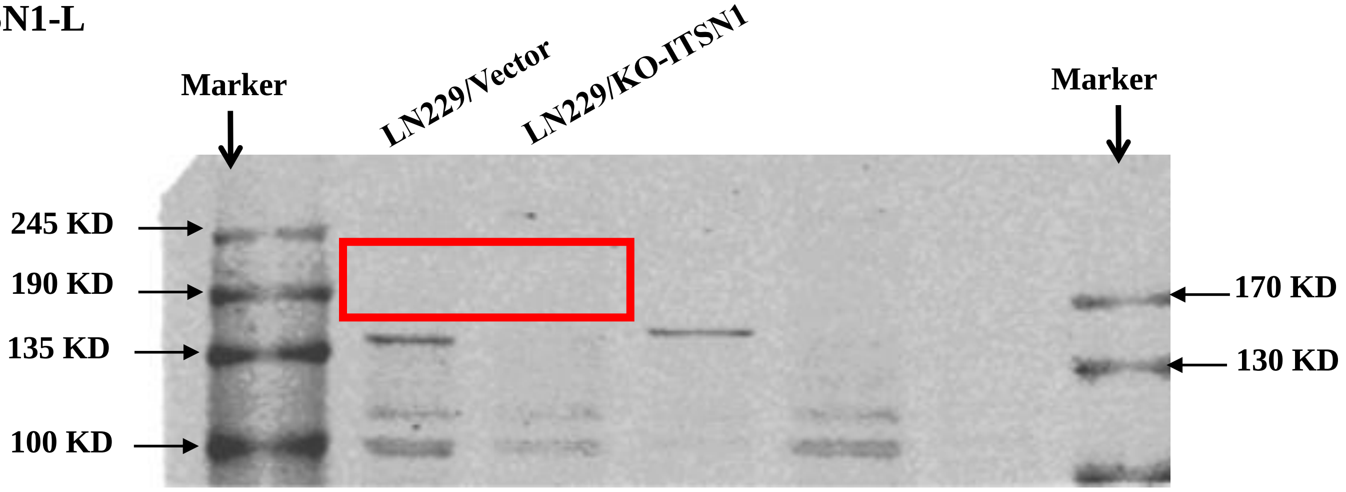

Full unedited gel for Figure 2d

ITSN1-S

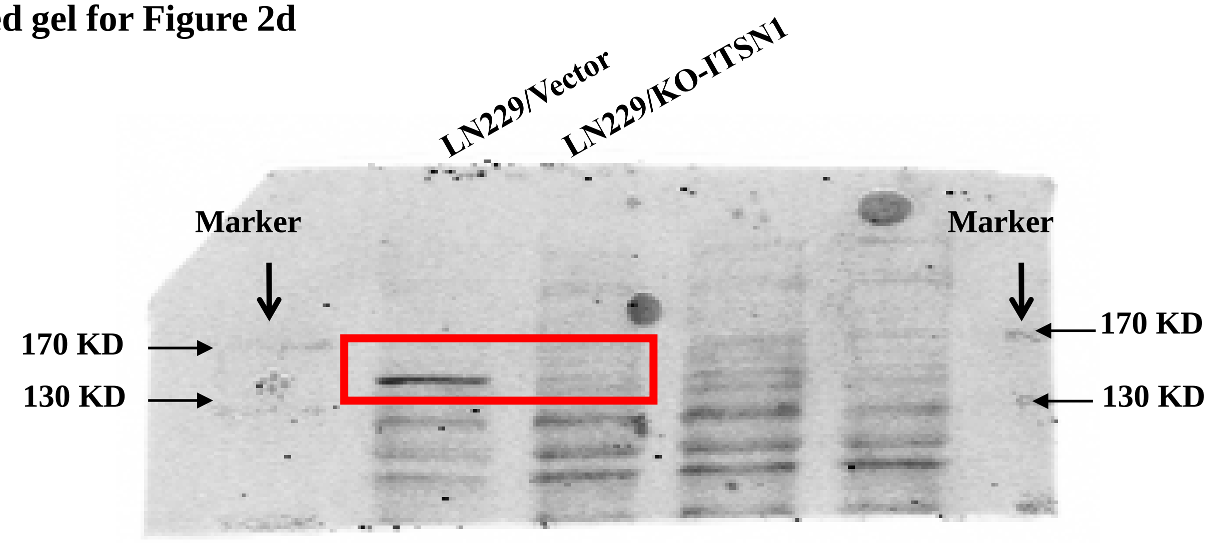

Full unedited gel for Figure 2d

β-actin

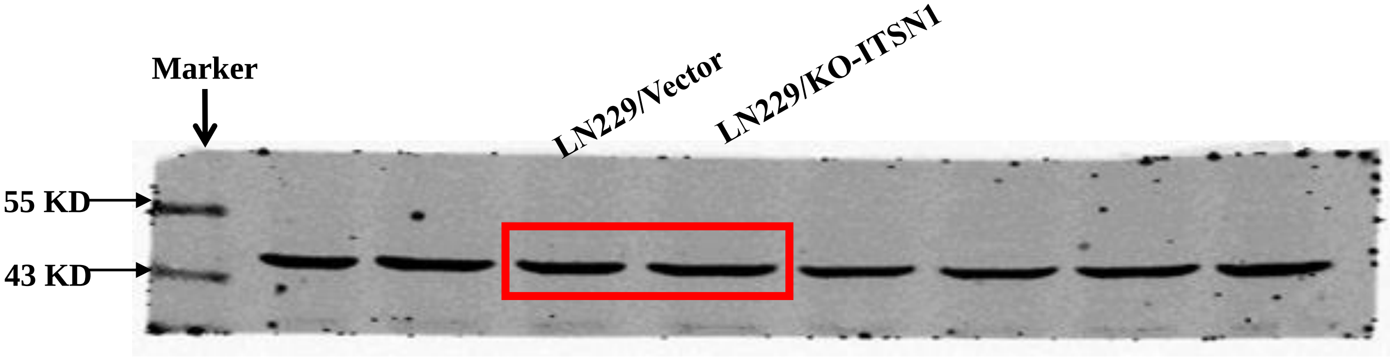

Full unedited gel for Figure 2e

ITSN1-L

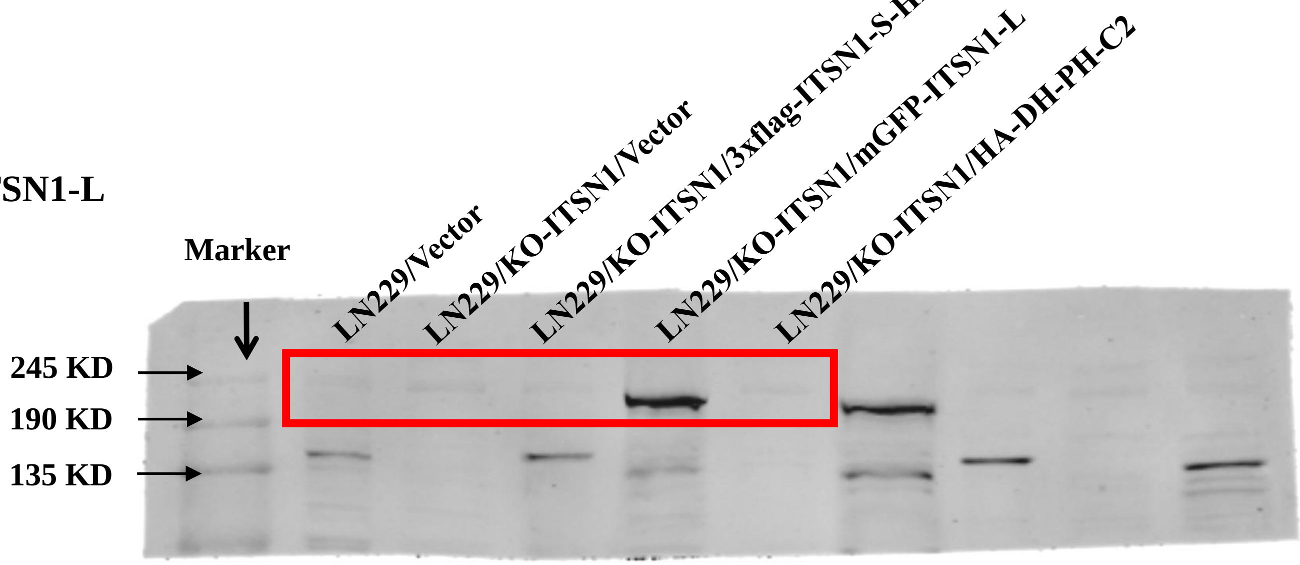

Full unedited gel for Figure 2e

ITSN1-S

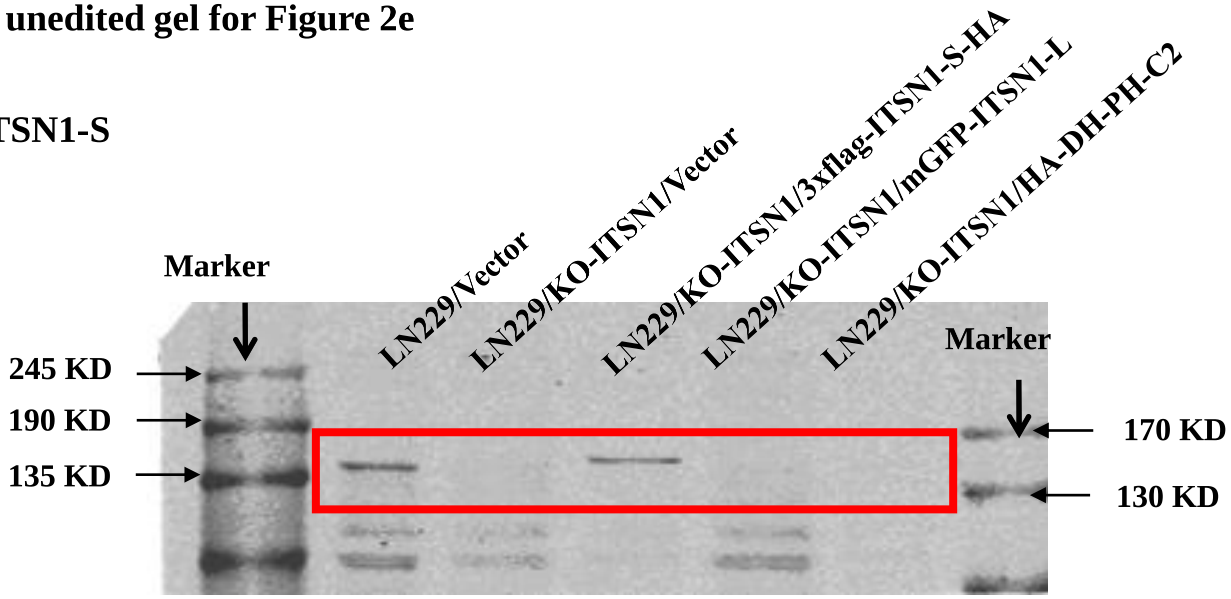

Full unedited gel for Figure 2e

flag

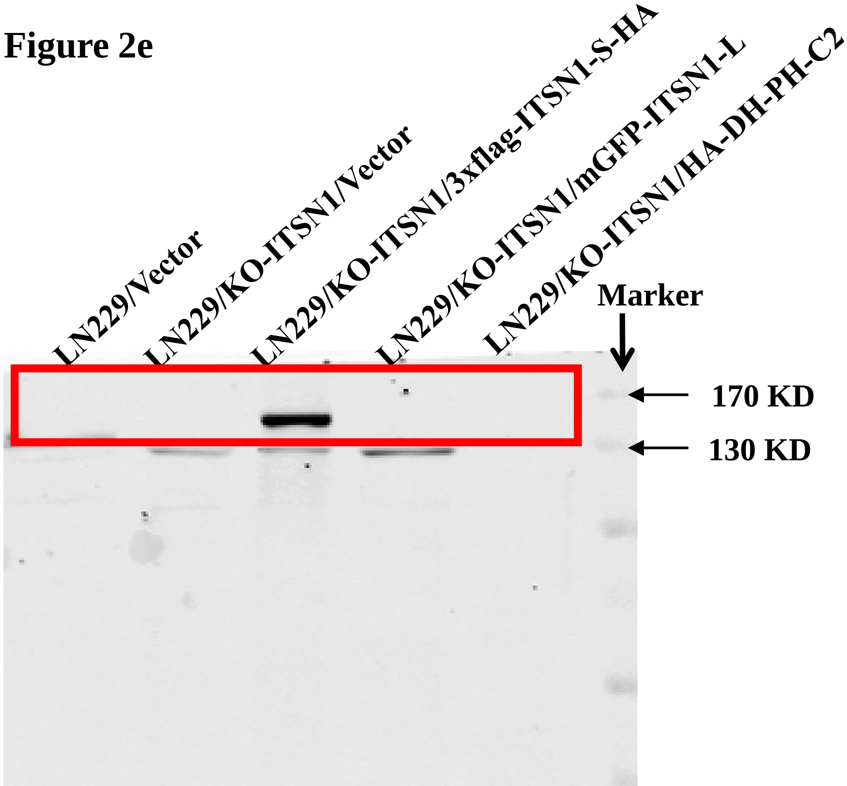

Full unedited gel for Figure 2e

HA

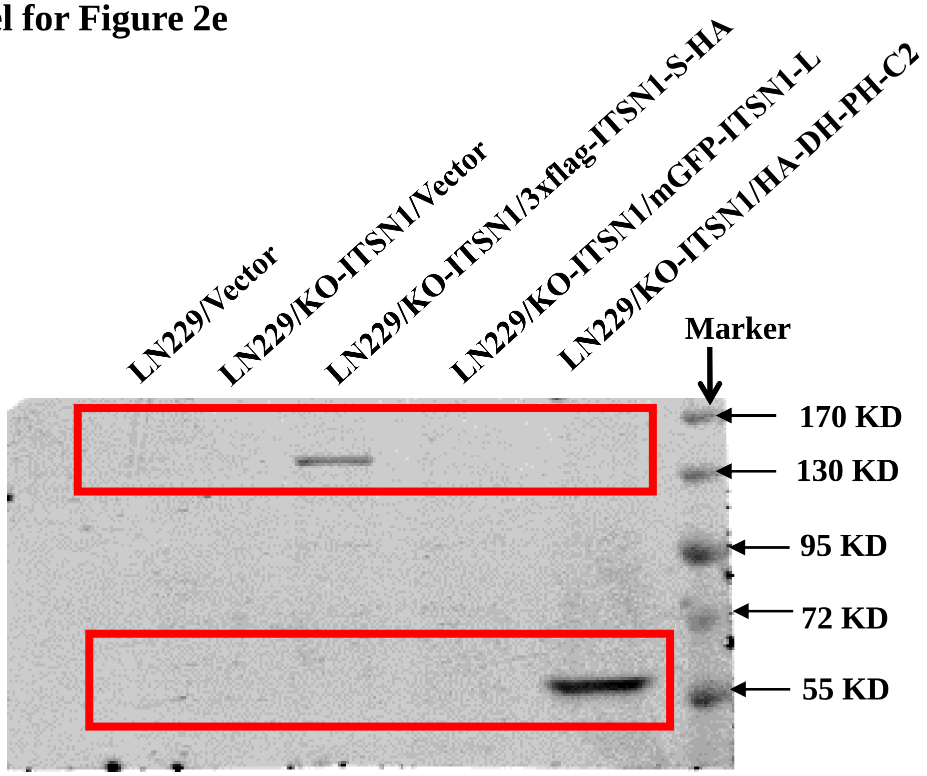

Full unedited gel for Figure 2e

β-actin

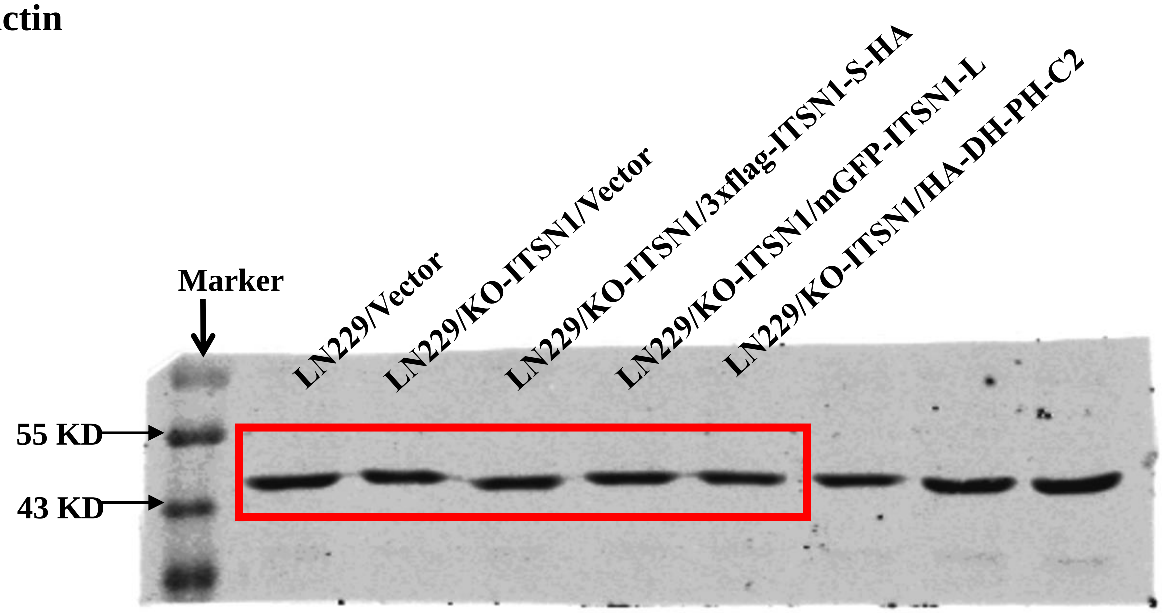

Full unedited gel for Figure 3a

ITSN1-S

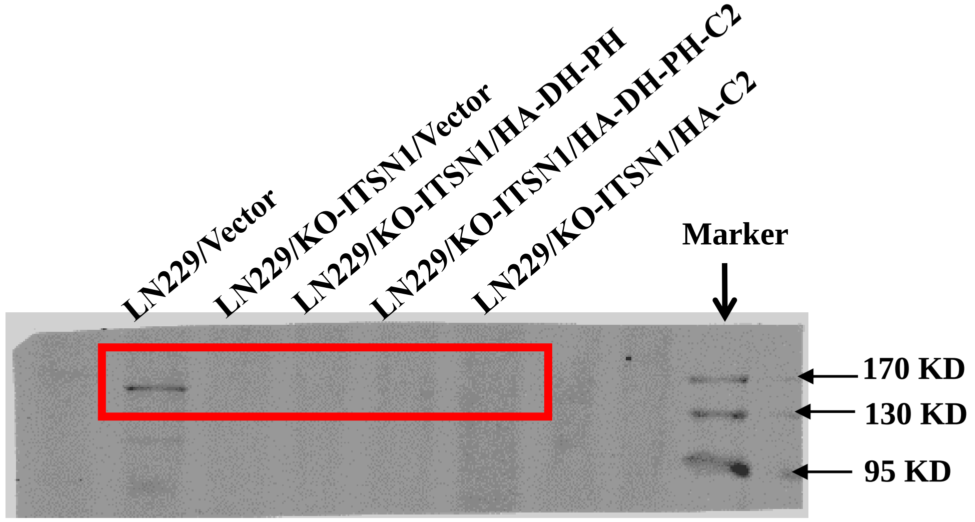

Full unedited gel for Figure 3a

HA

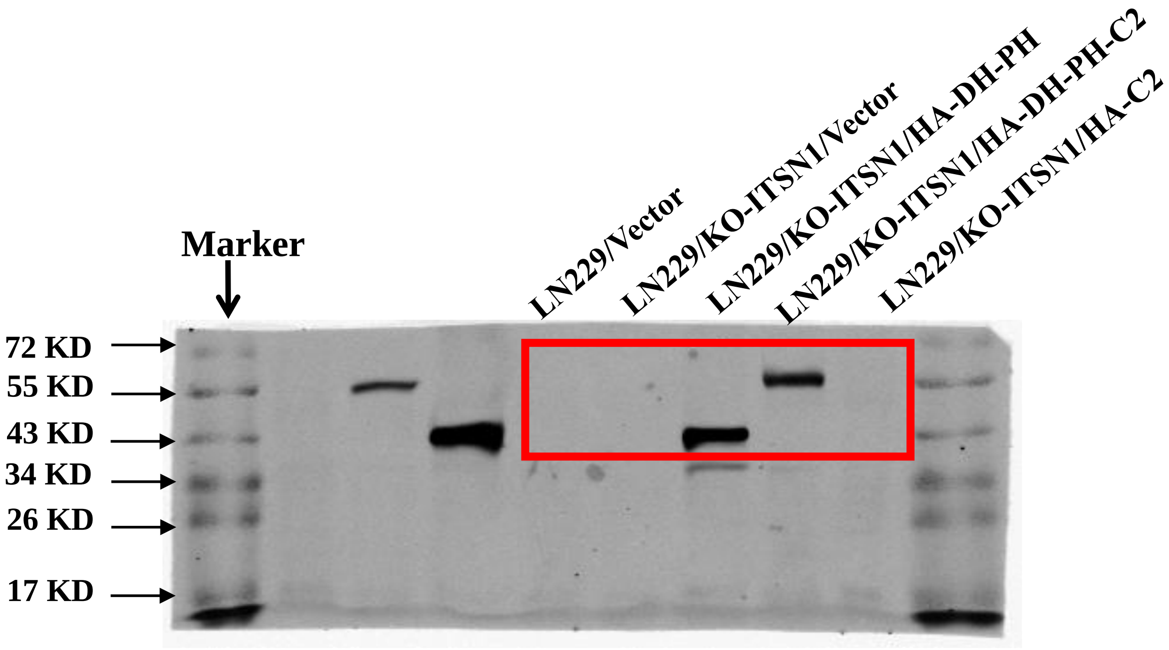

Full unedited gel for Figure 3a

HA

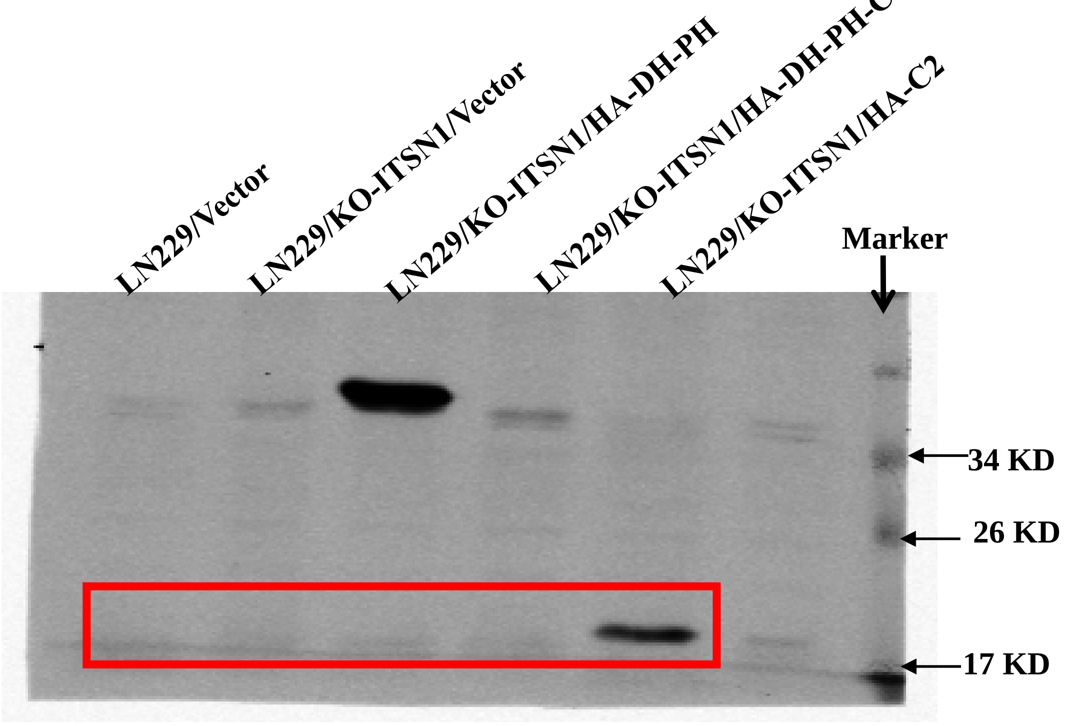

Full unedited gel for Figure 3a

β-actin

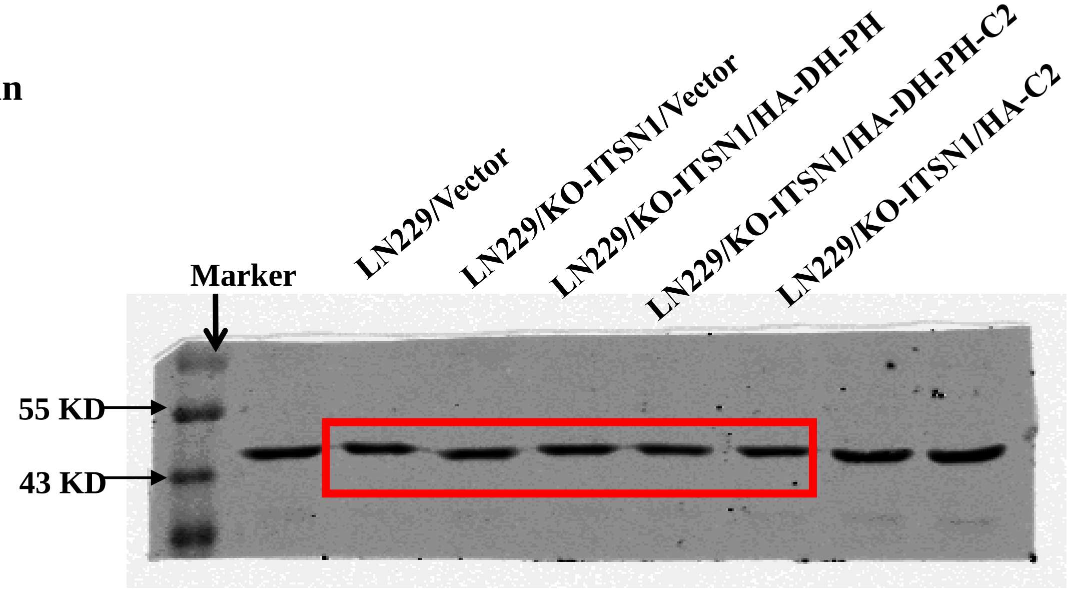

Full unedited gel for Figure 4a

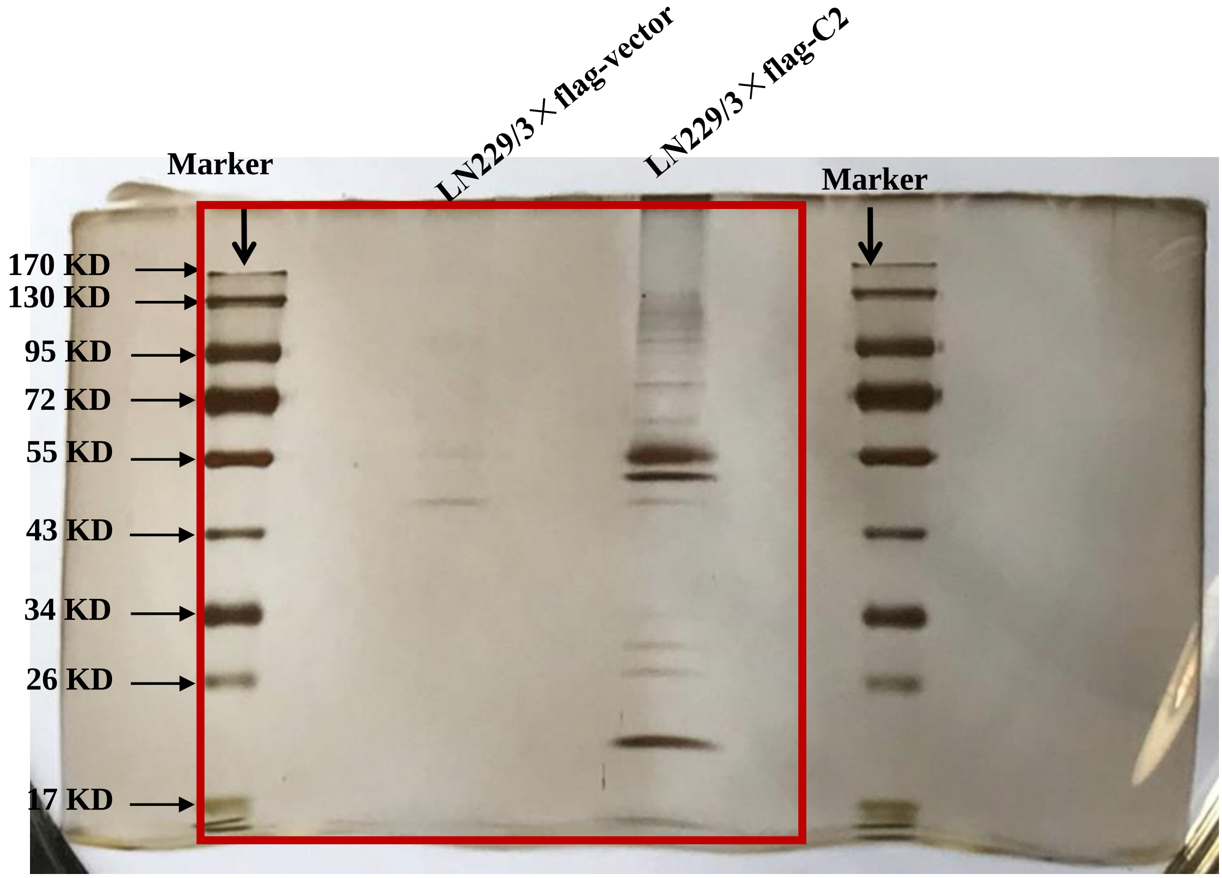

Full unedited gel for Figure 4b

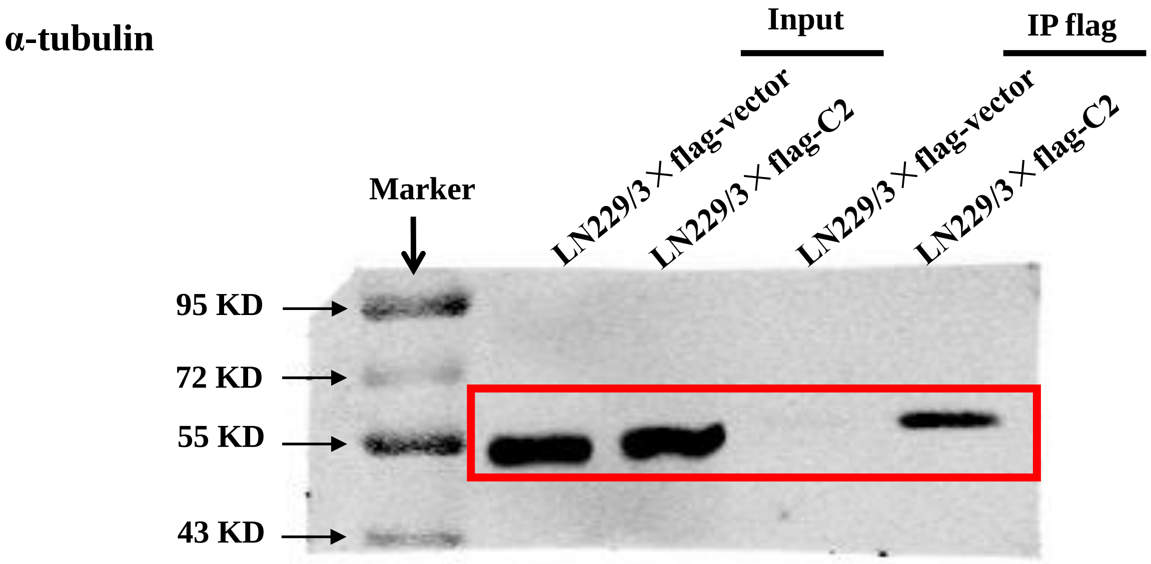

Full unedited gel for Figure 4b

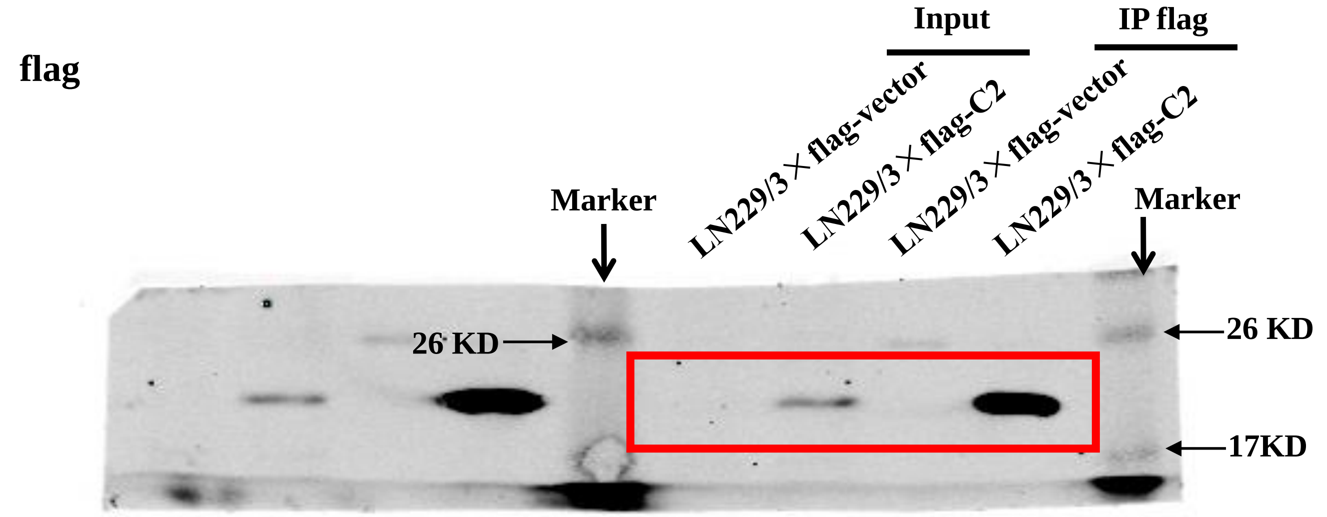

Full unedited gel for Figure 4c

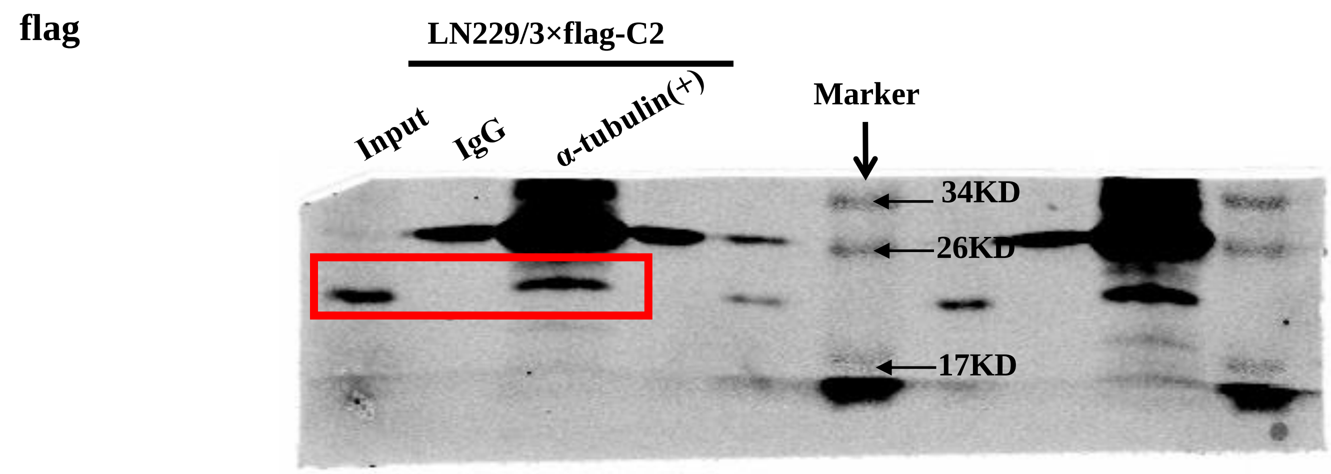

Full unedited gel for Figure 4c

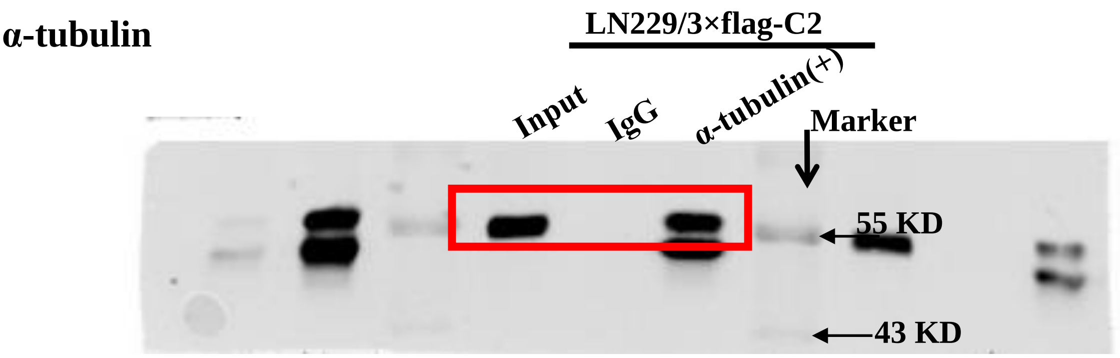

Full unedited gel for Figure 4d

ac-tubulin

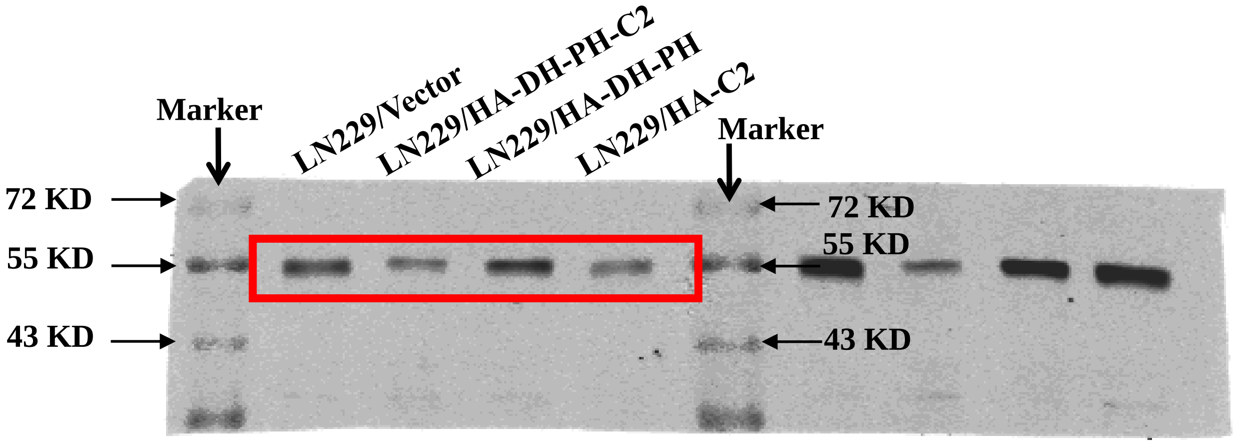

Full unedited gel for Figure 4d

α-tubulin

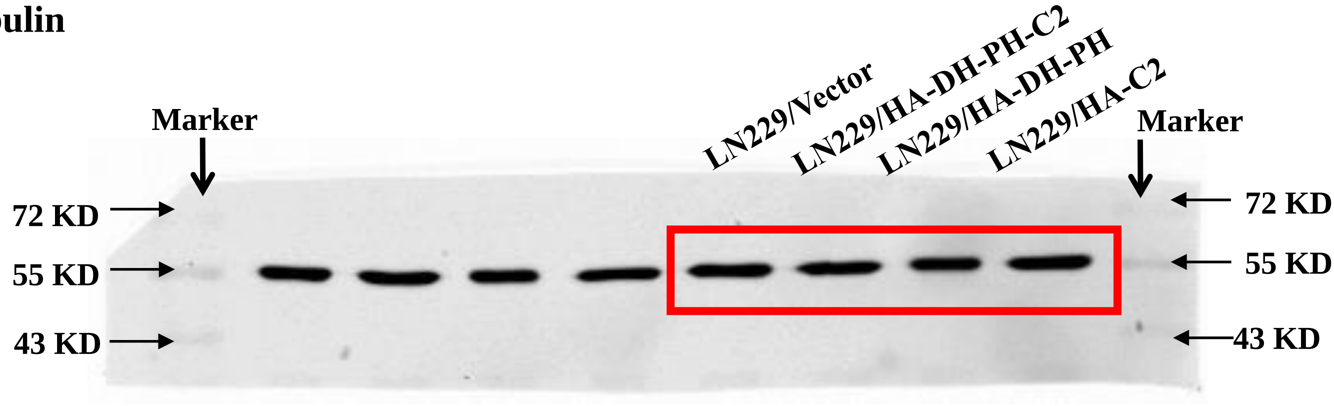

Full unedited gel for Figure 4d

β-actin

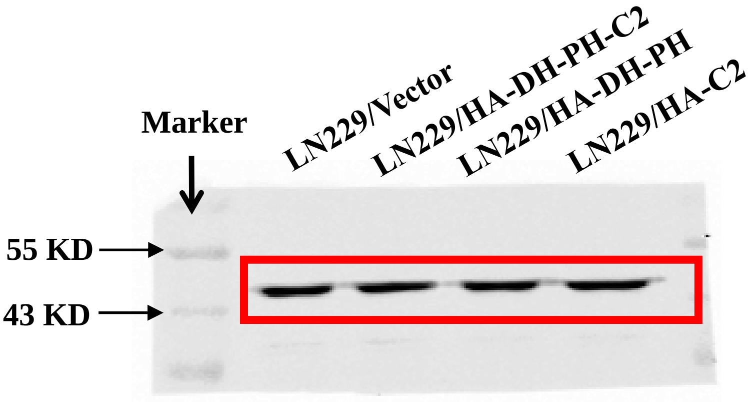

Full unedited gel for Figure 4e

ac-tubulin

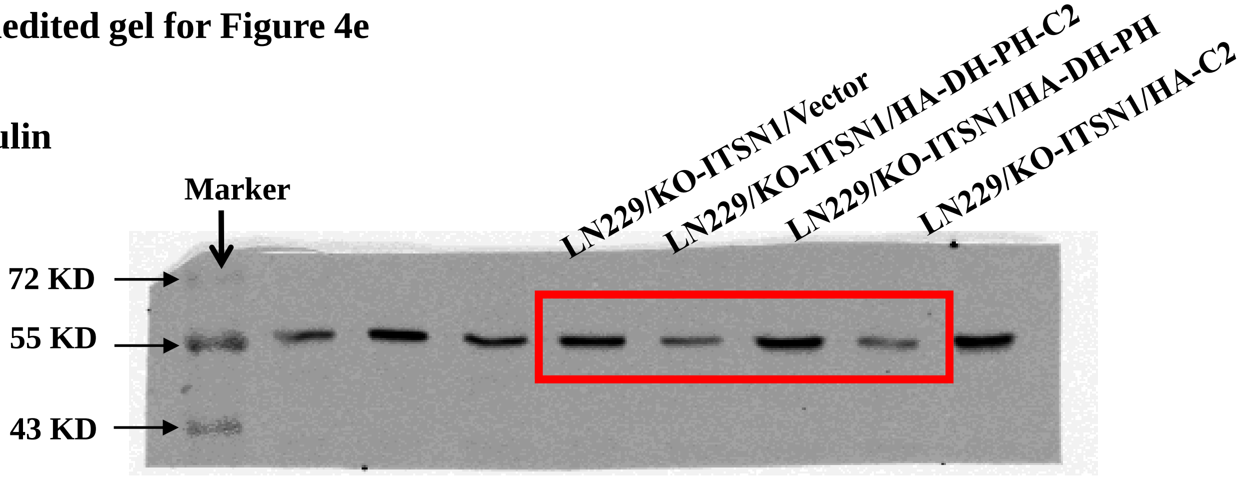

Full unedited gel for Figure 4e

α-tubulin

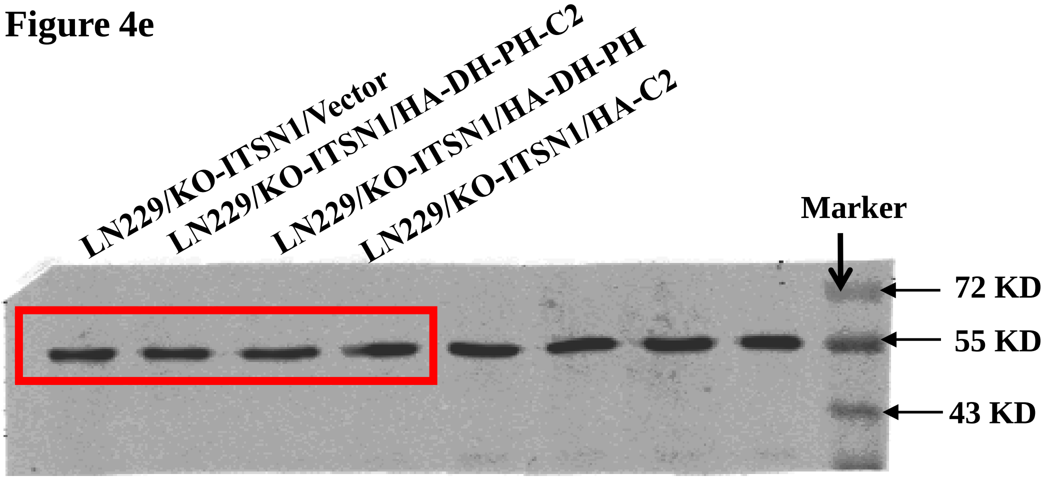

Full unedited gel for Figure 4e

β-actin

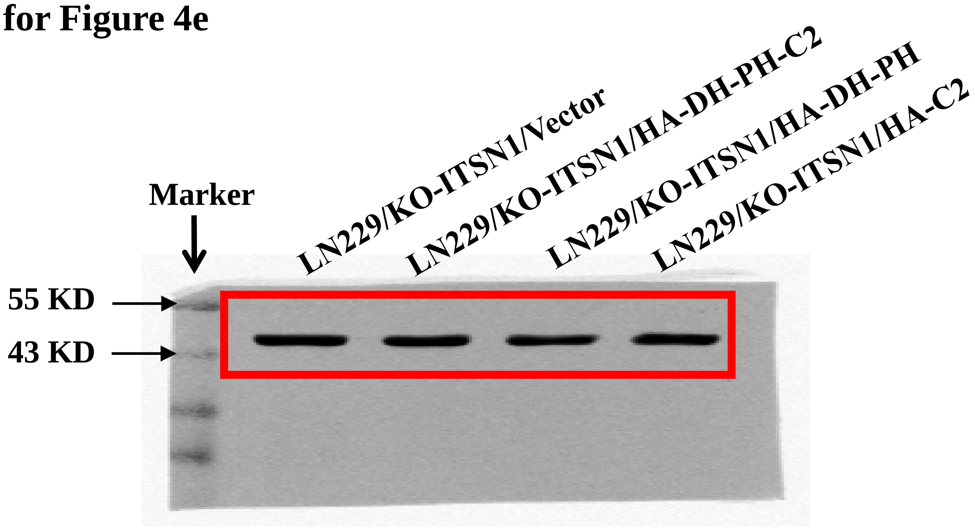

Full unedited gel for Figure 5a left

HDAC6

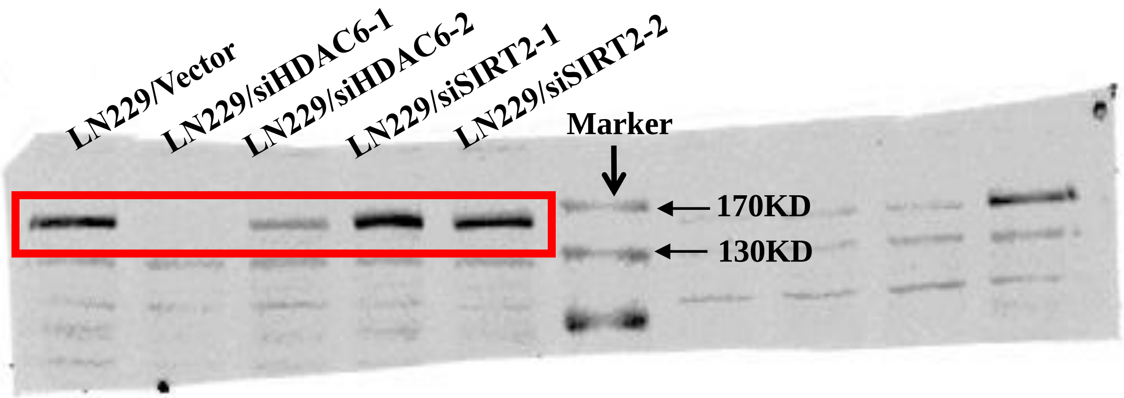

Full unedited gel for Figure 5a left

SIRT2

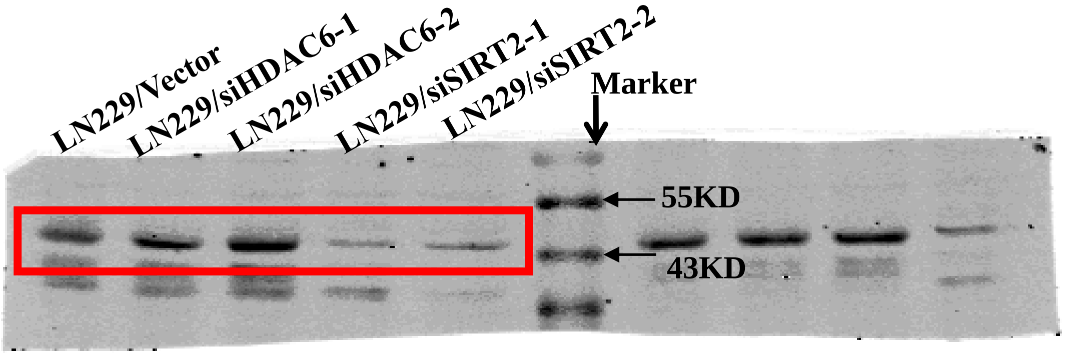

Full unedited gel for Figure 5a left

ac-tubulin

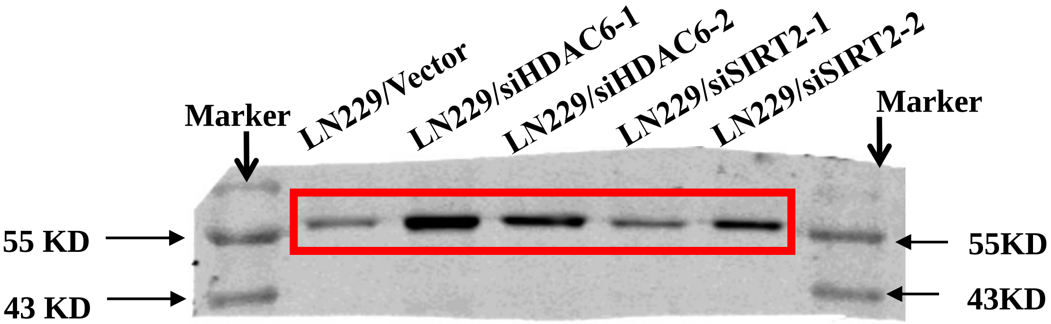

Full unedited gel for Figure 5a left

α-tubulin

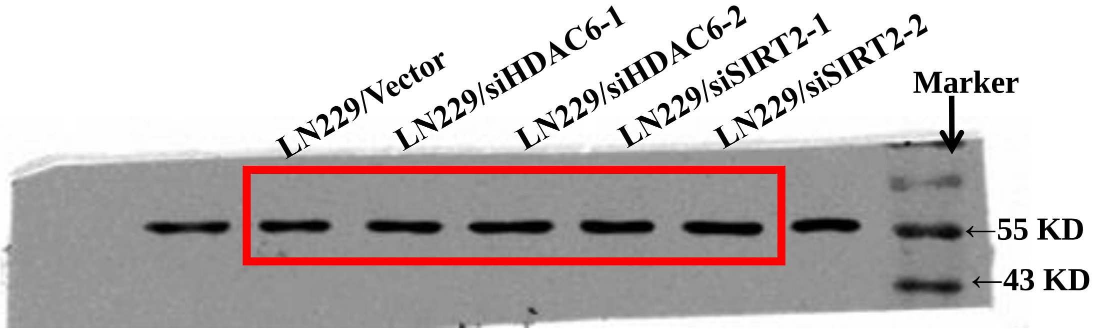

Full unedited gel for Figure 5a left

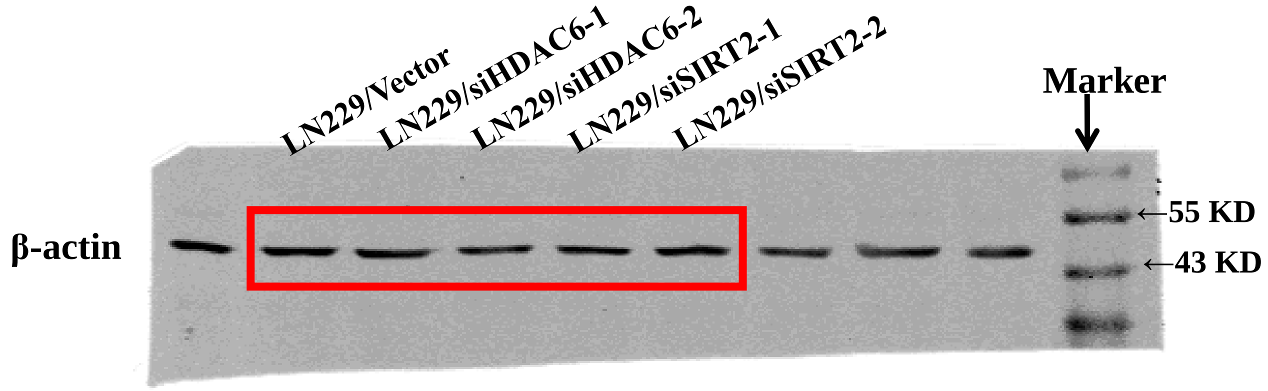

Full unedited gel for Figure 5a right

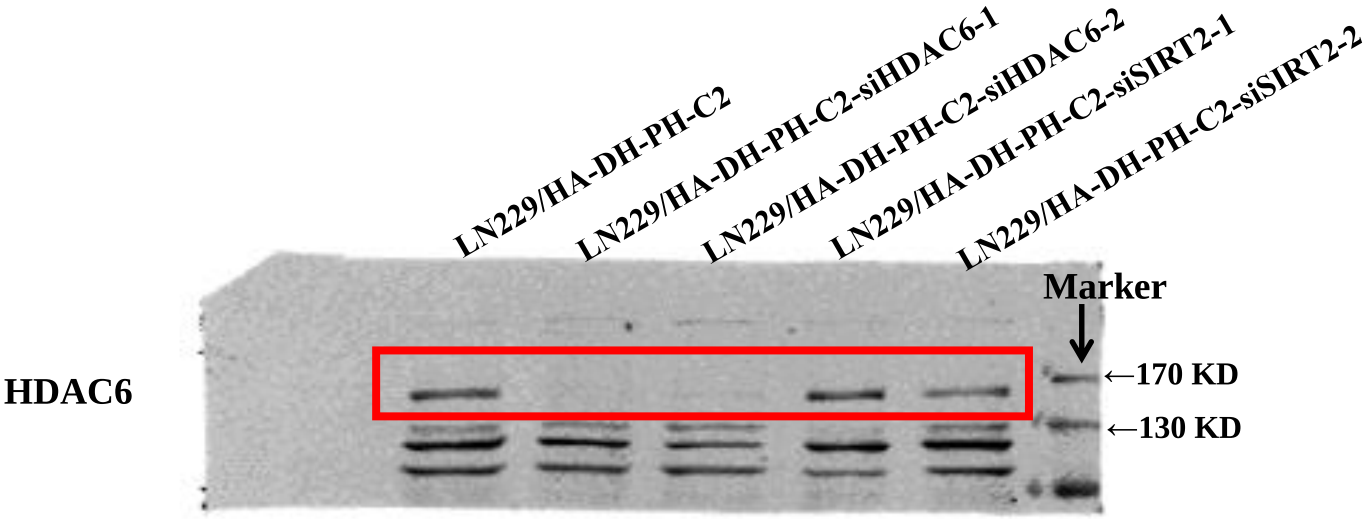

Full unedited gel for Figure 5a right

SIRT2

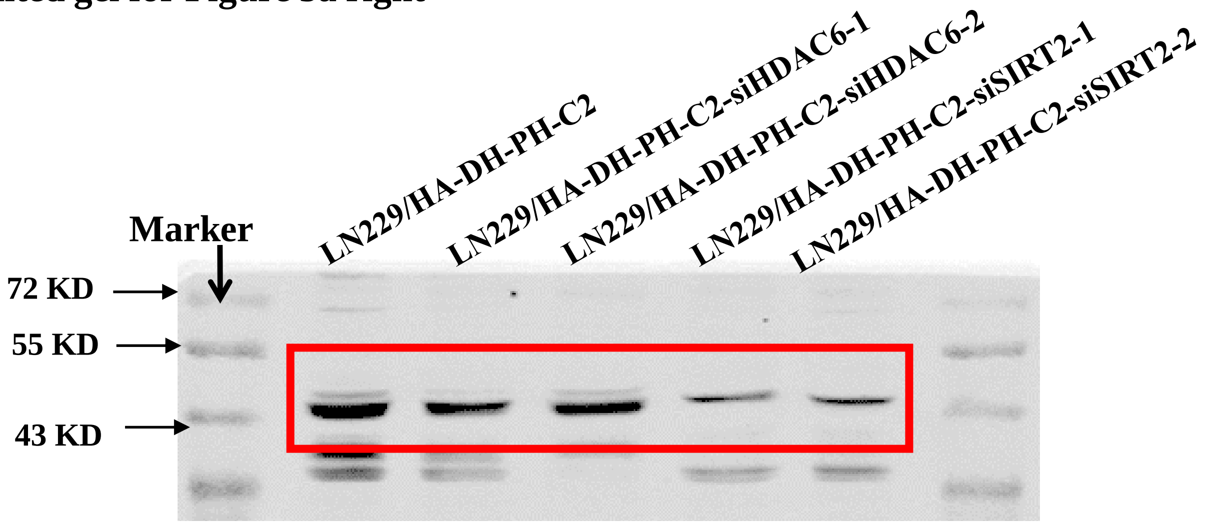

Full unedited gel for Figure 5a right

ac-tubulin

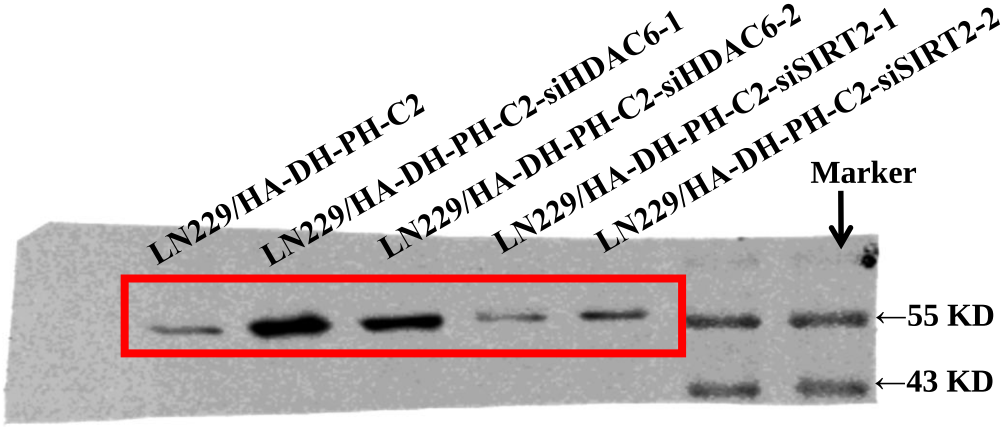

Full unedited gel for Figure 5a right

α-tubulin

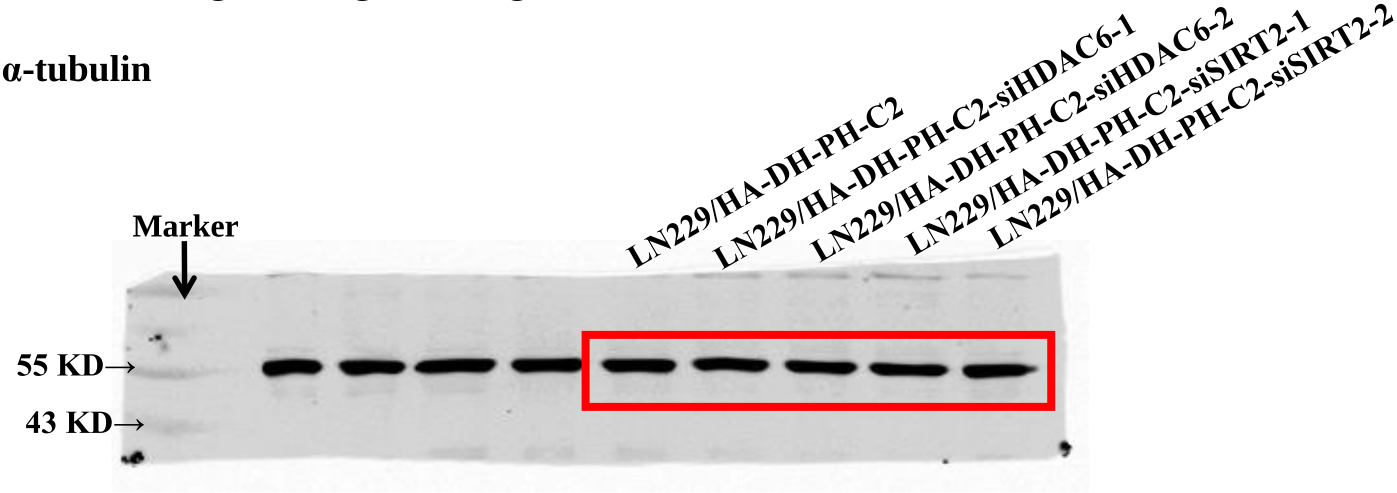

Full unedited gel for Figure 5a right

β-actin

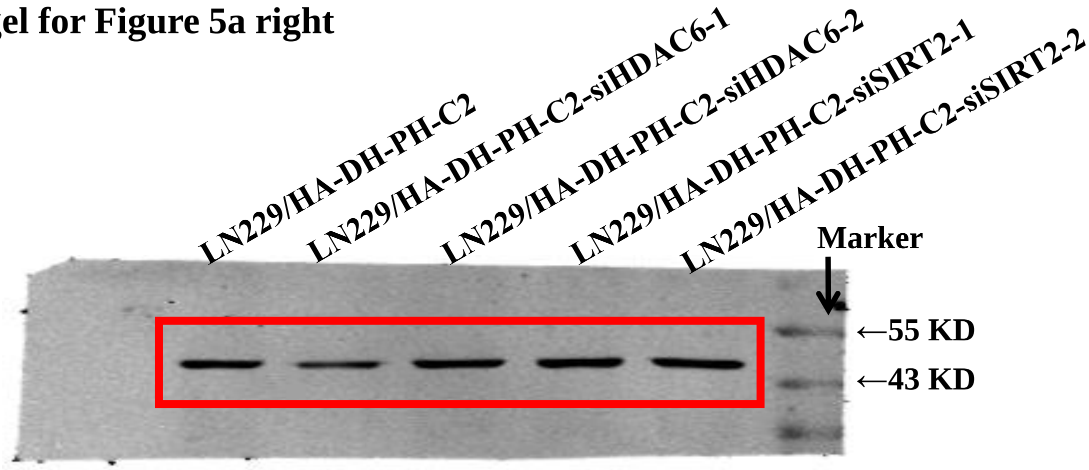

Full unedited gel for Figure 5b

HDAC6

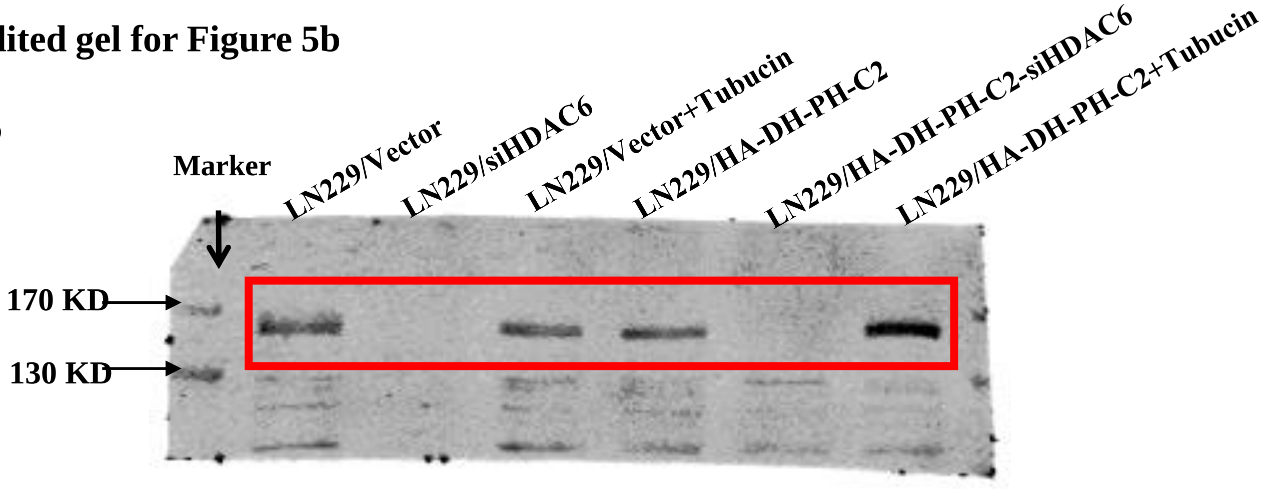

Full unedited gel for Figure 5b

ac-tubulin

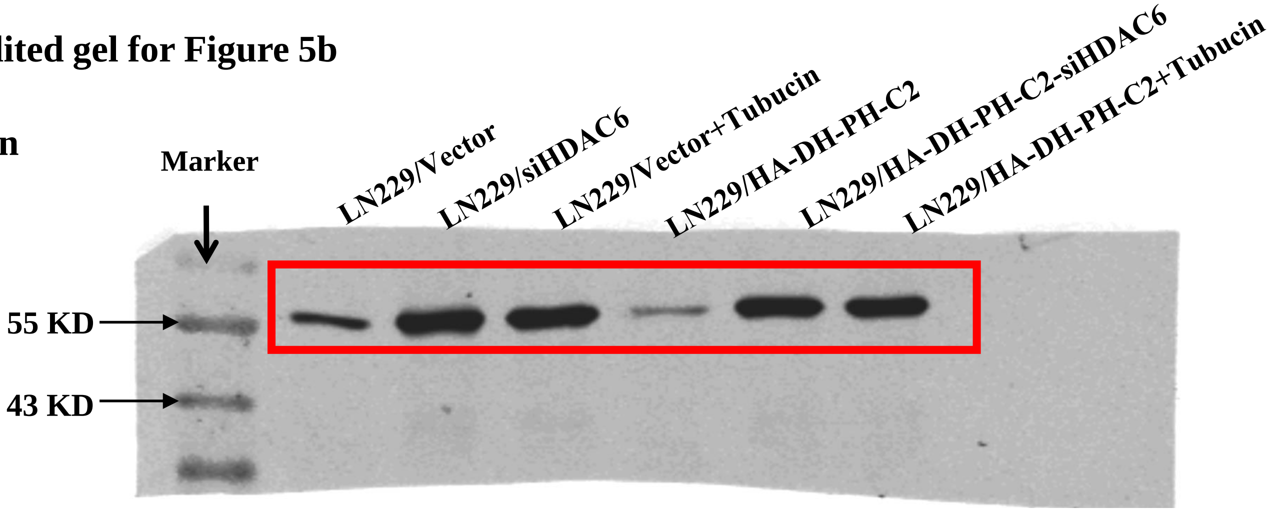

Full unedited gel for Figure 5b

α-tubulin

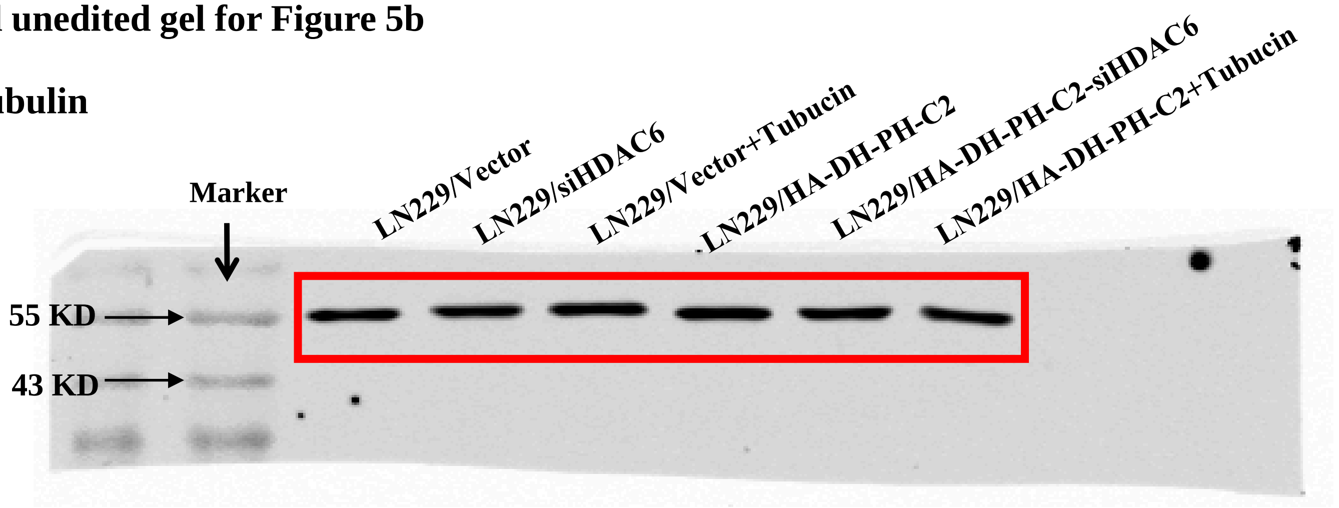

Full unedited gel for Figure 5b

β-actin

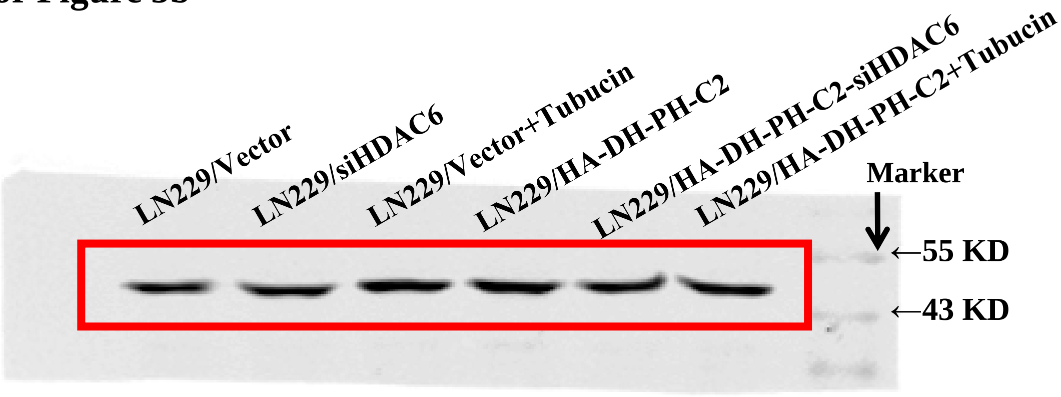

Full unedited gel for Figure 5c

ac-tubulin

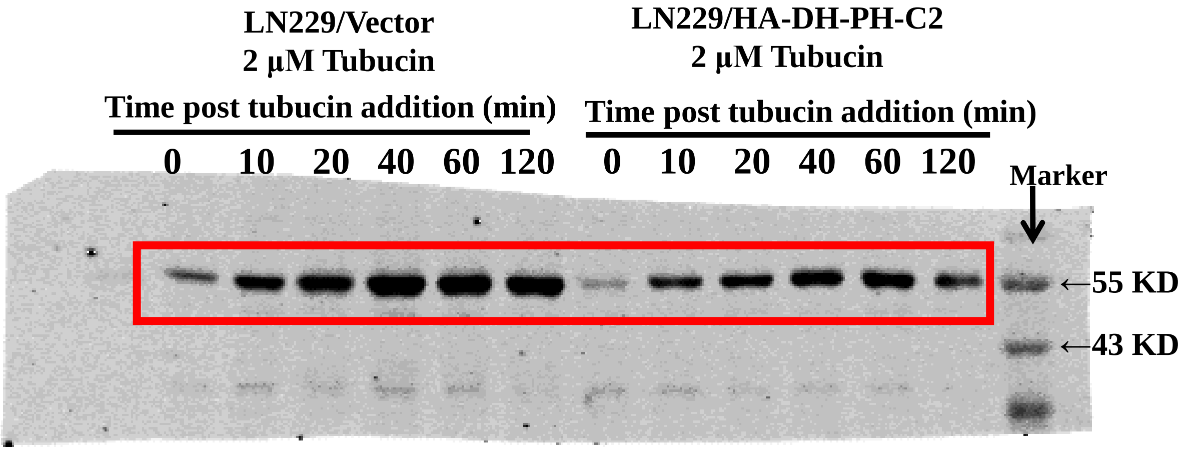

Full unedited gel for Figure 5c

α-tubulin

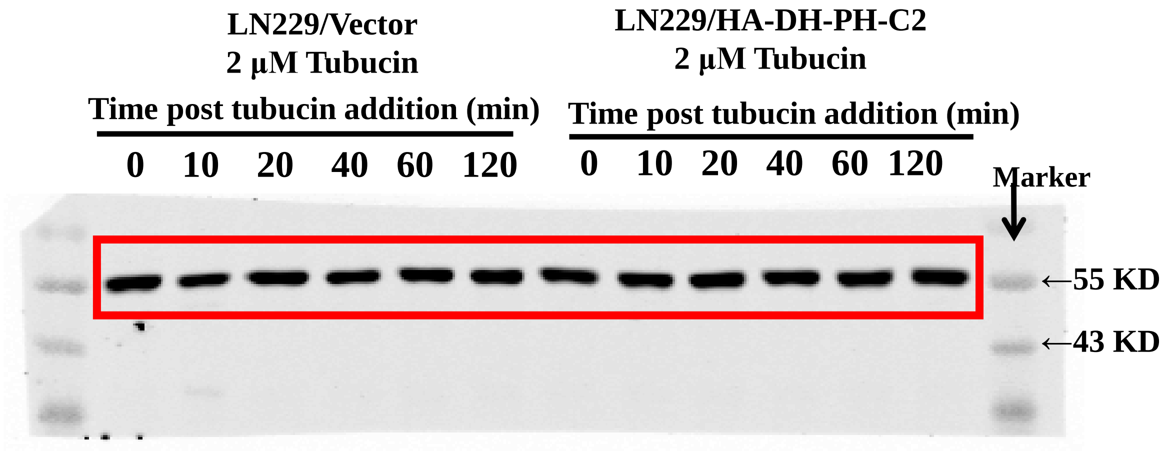

Full unedited gel for Figure 5c

β-actin

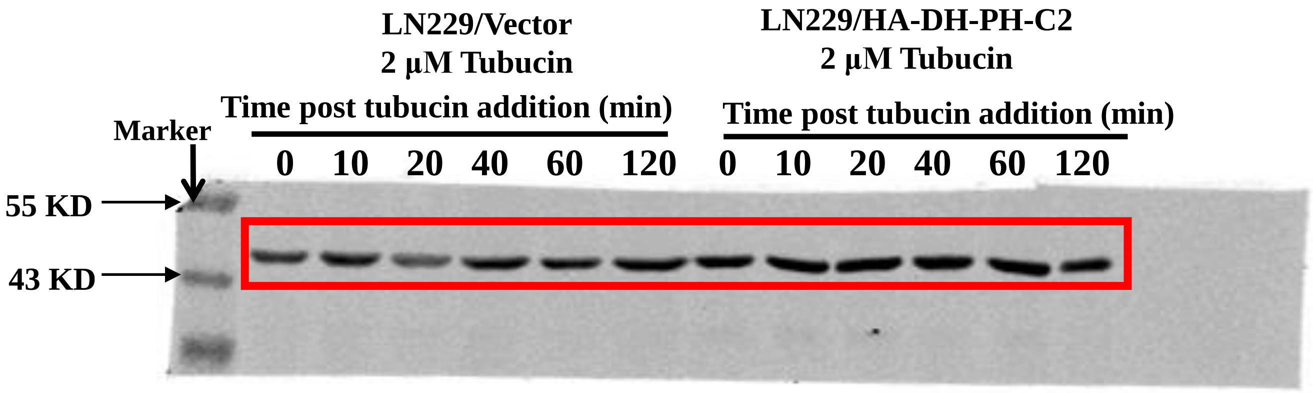

Full unedited gel for Figure 5d

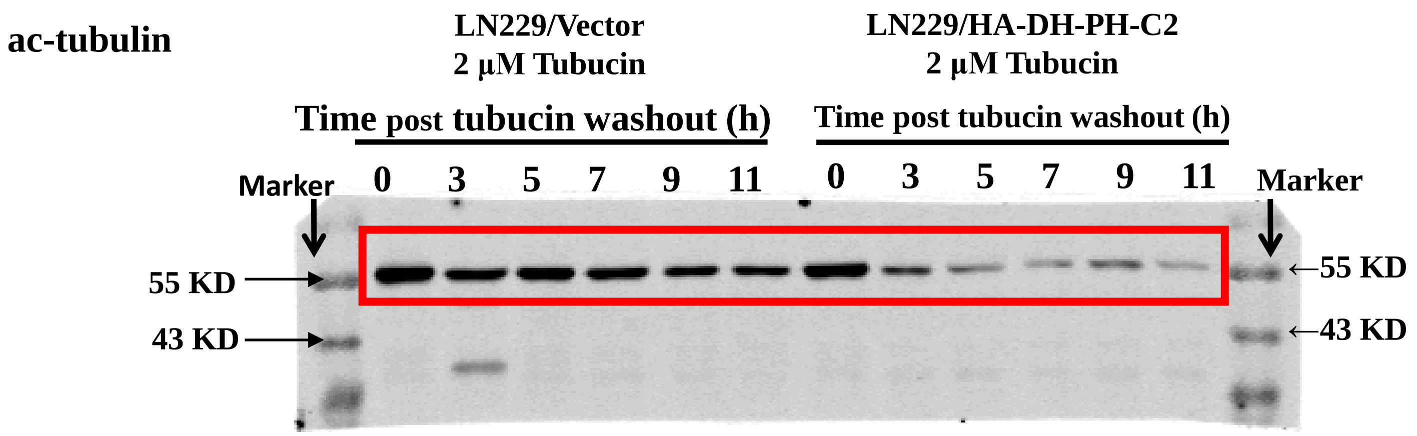

Full unedited gel for Figure 5d

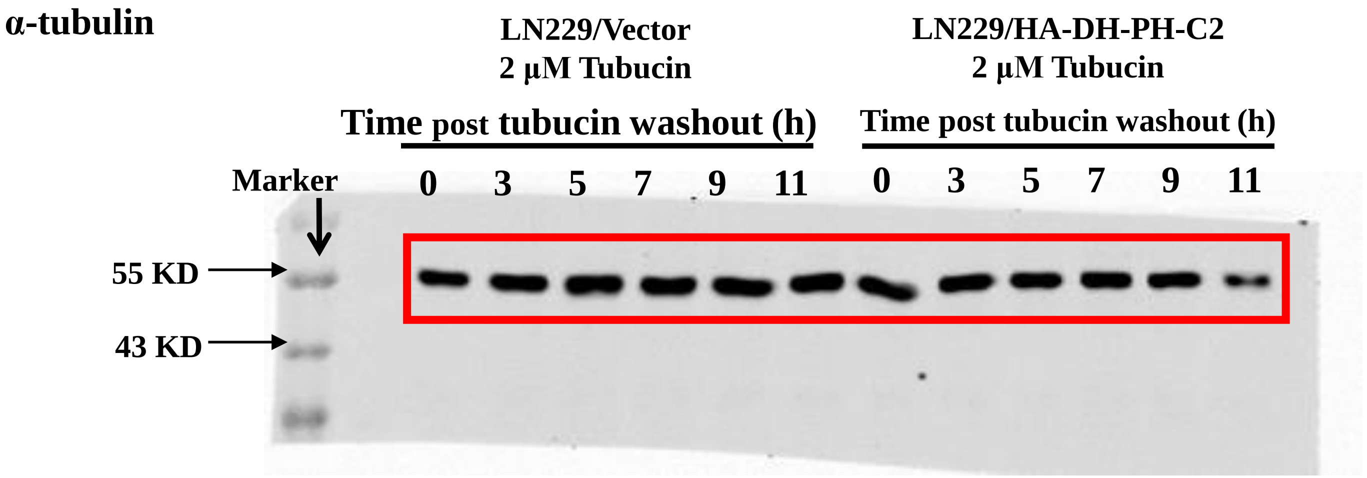

Full unedited gel for Figure 5d

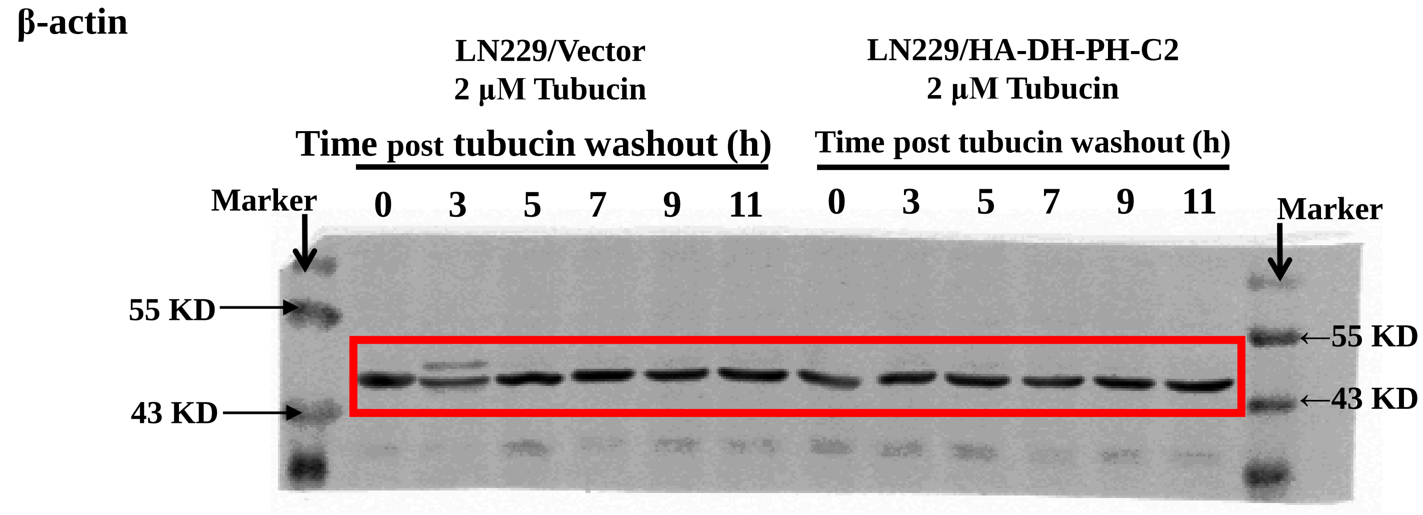

Full unedited gel for Figure 6c

p-FAK

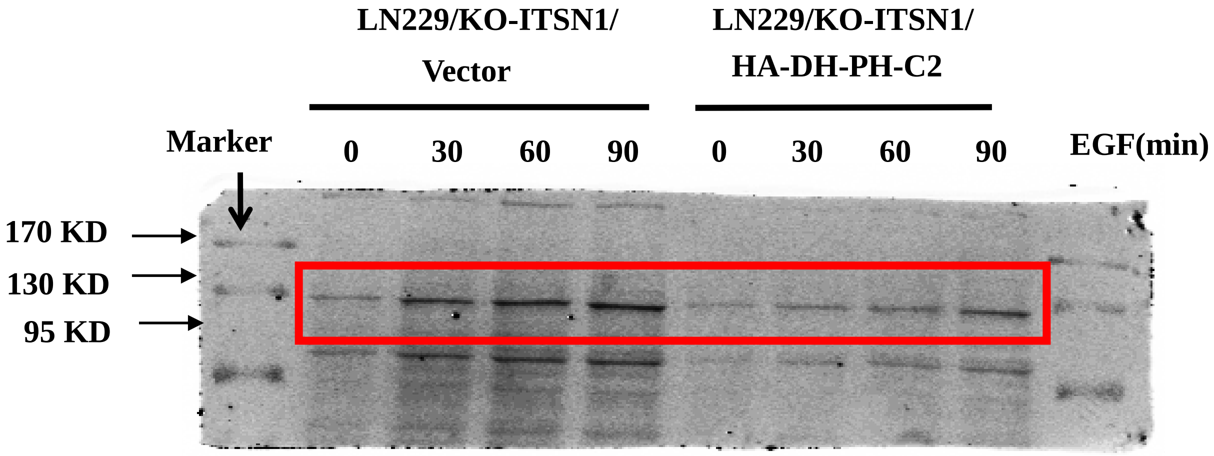

Full unedited gel for Figure 6c

FAK

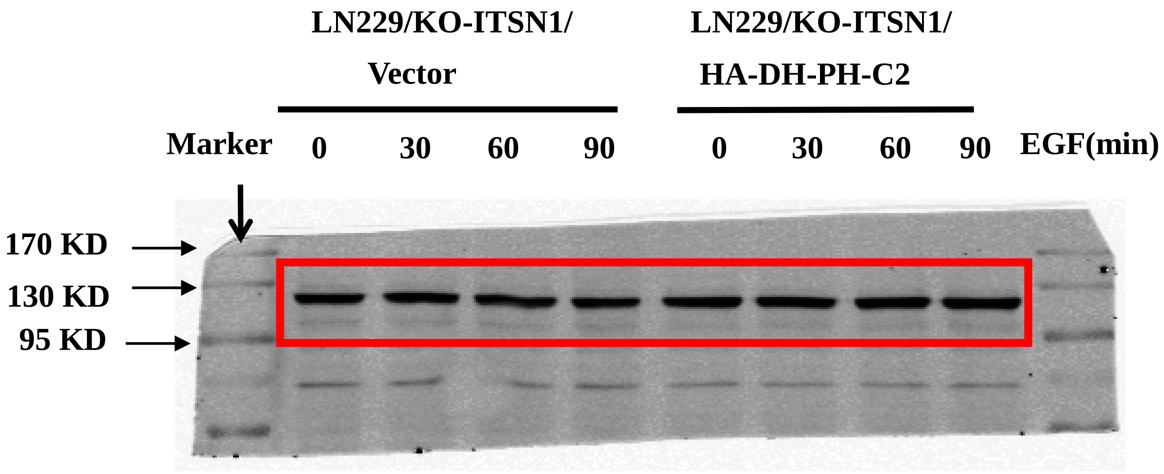

Full unedited gel for Figure 6c

p-integrin β3

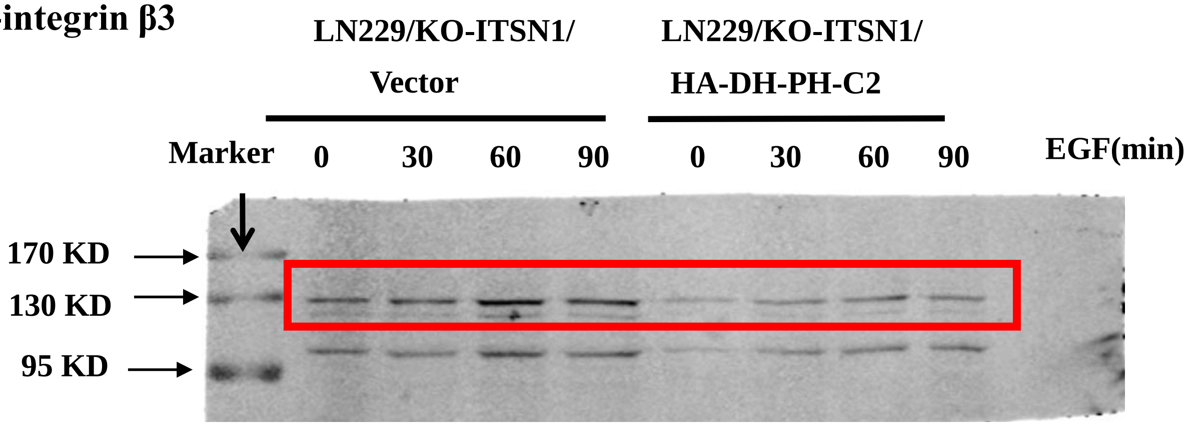

Full unedited gel for Figure 6c

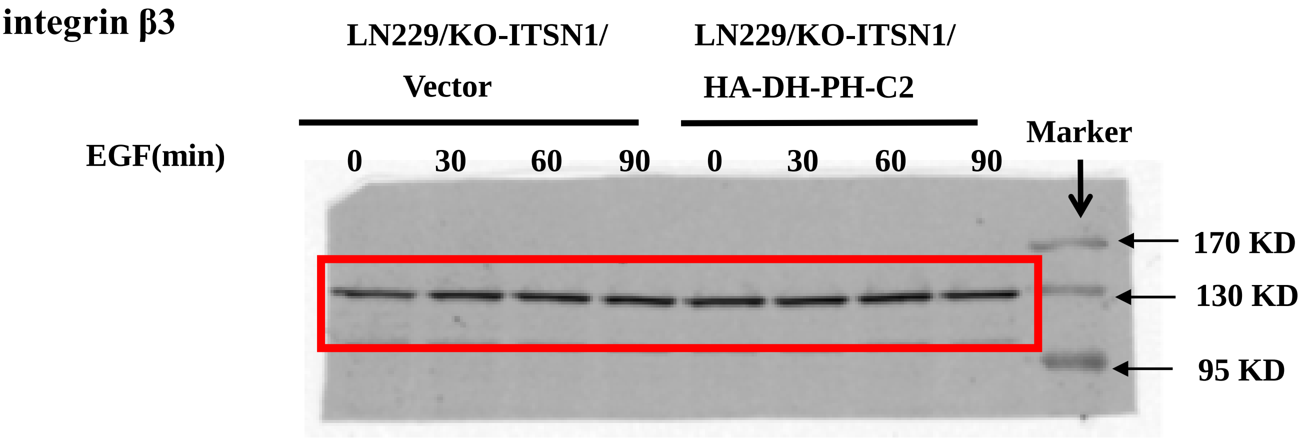

Full unedited gel for Figure 6c

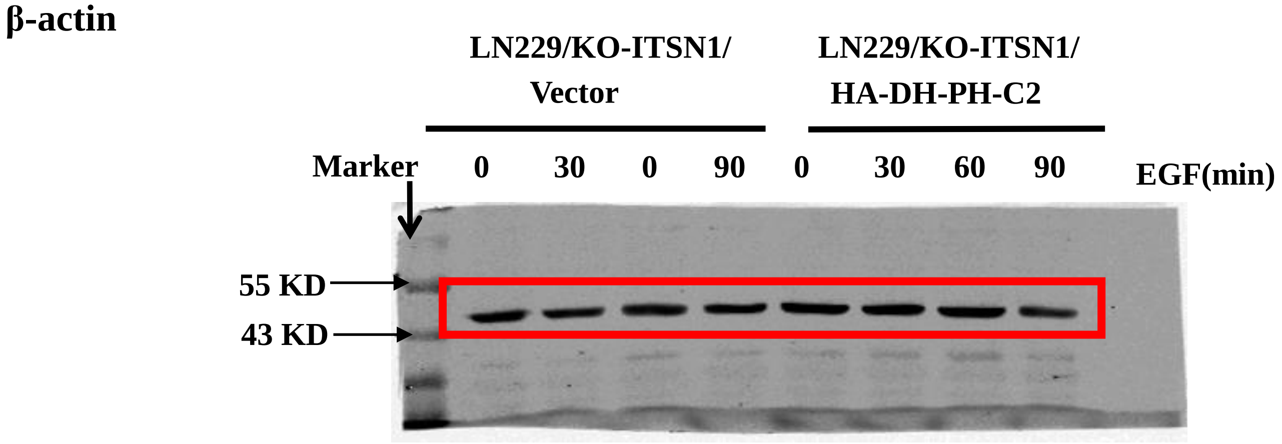

Full unedited gel for Figure 6e

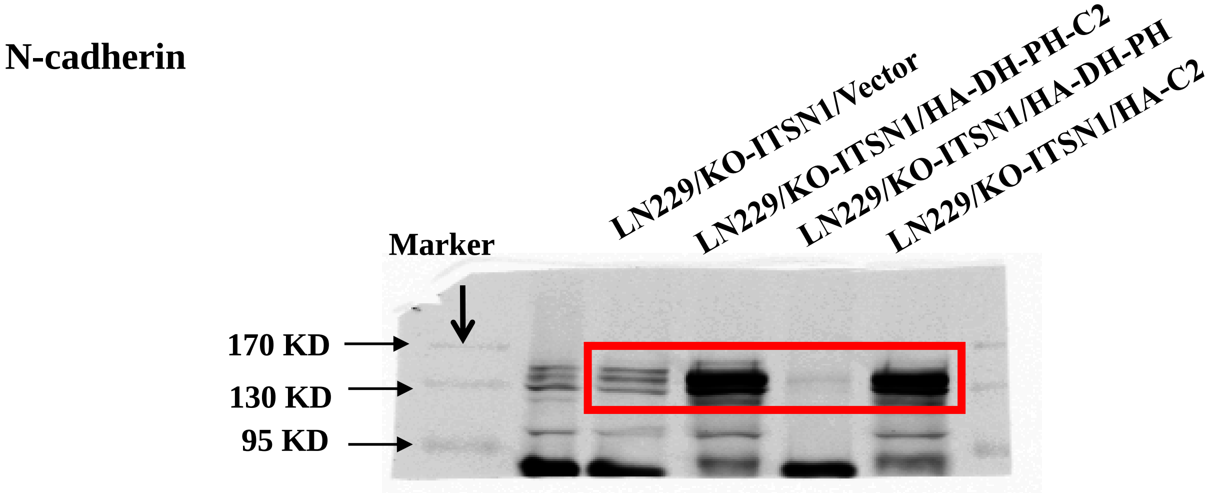

Full unedited gel for Figure 6e

β-catenin

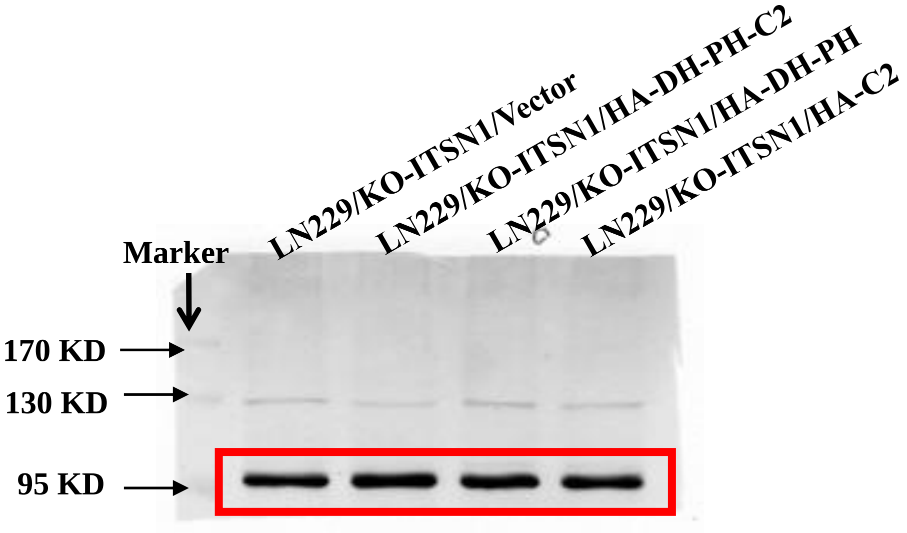

Full unedited gel for Figure 6e

Snail

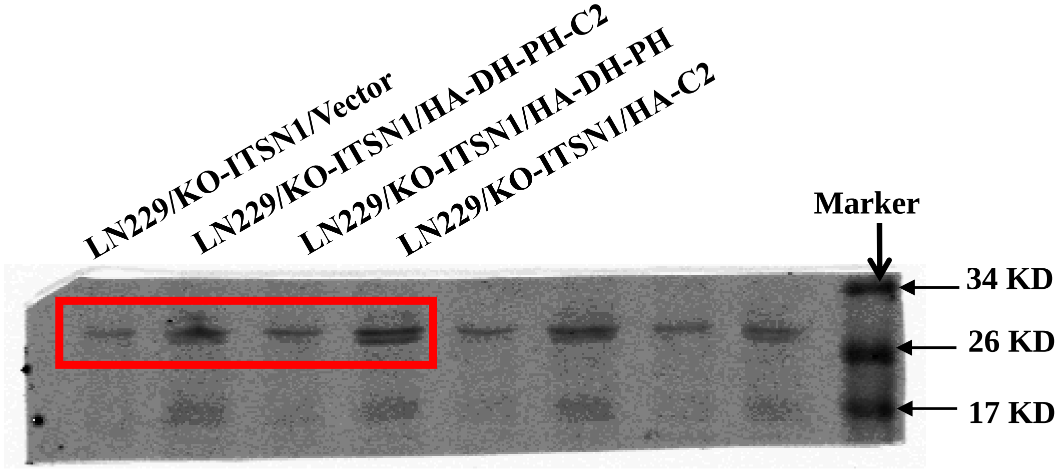

Full unedited gel for Figure 6e

Slug

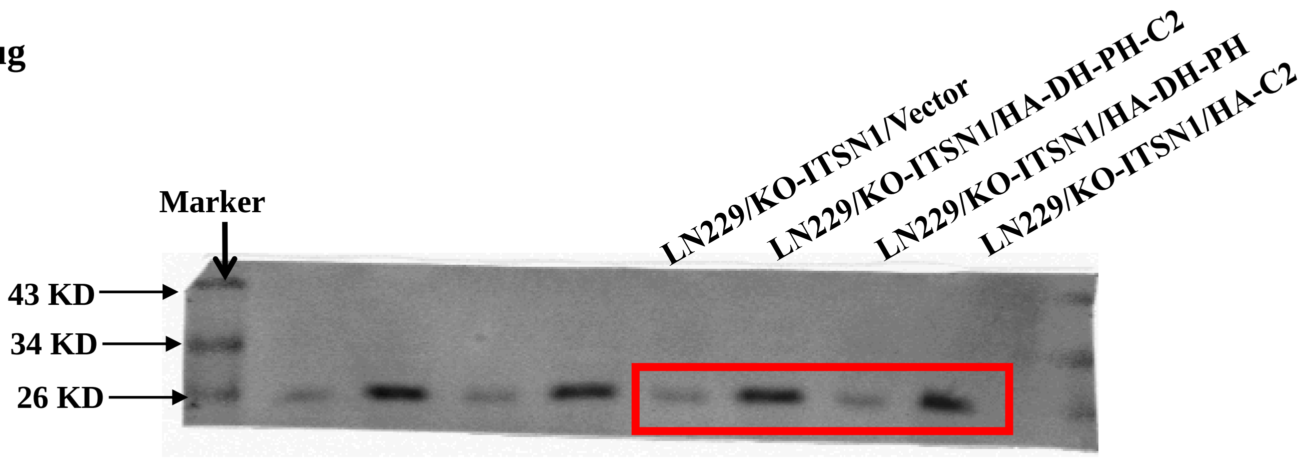

Full unedited gel for Figure 6e

Twist

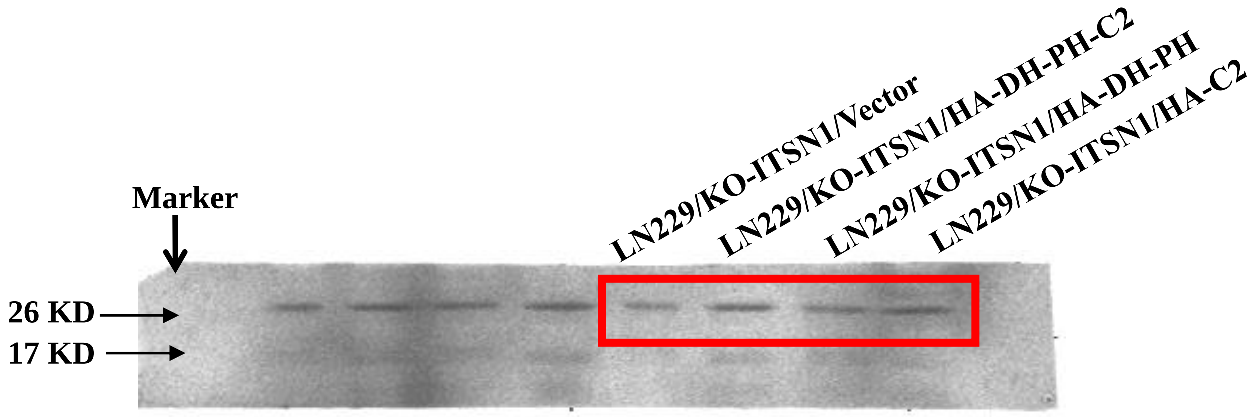

Full unedited gel for Figure 6e

β-actin

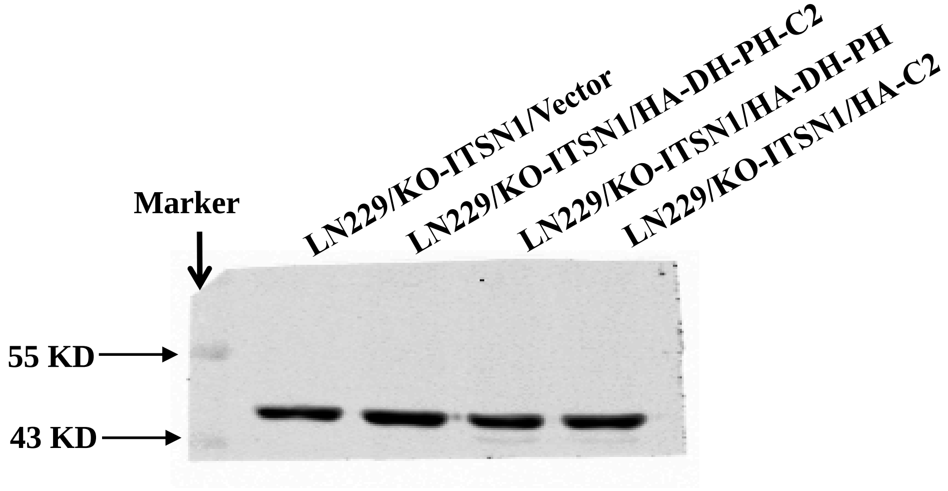

Full unedited gel for Figure 7a

ANXA2

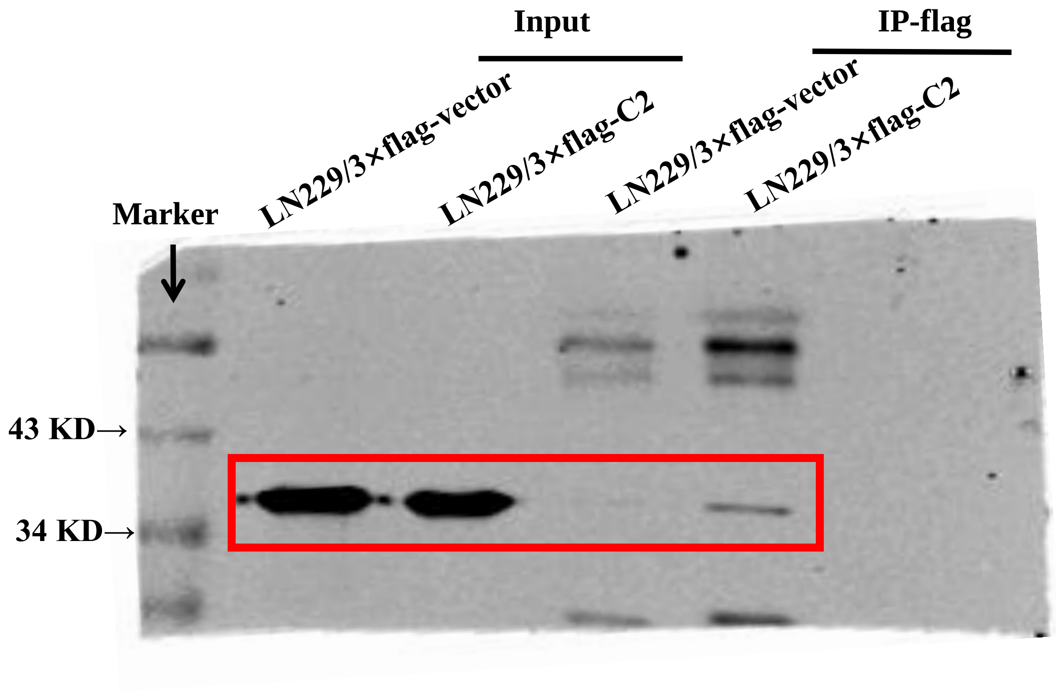

Full unedited gel for Figure 7a

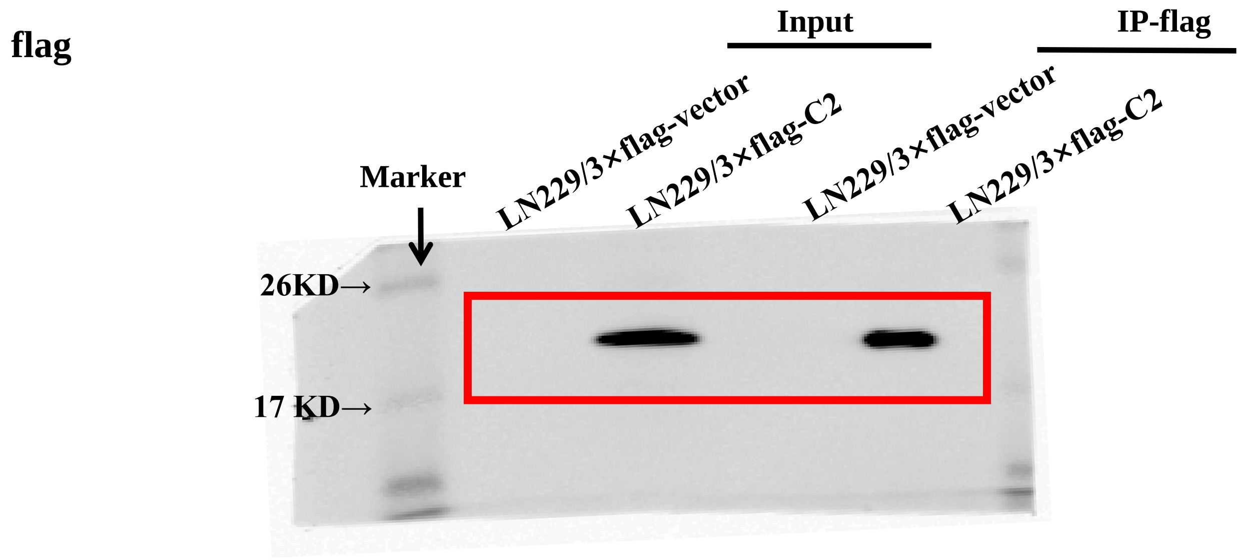

Full unedited gel for Figure 7b

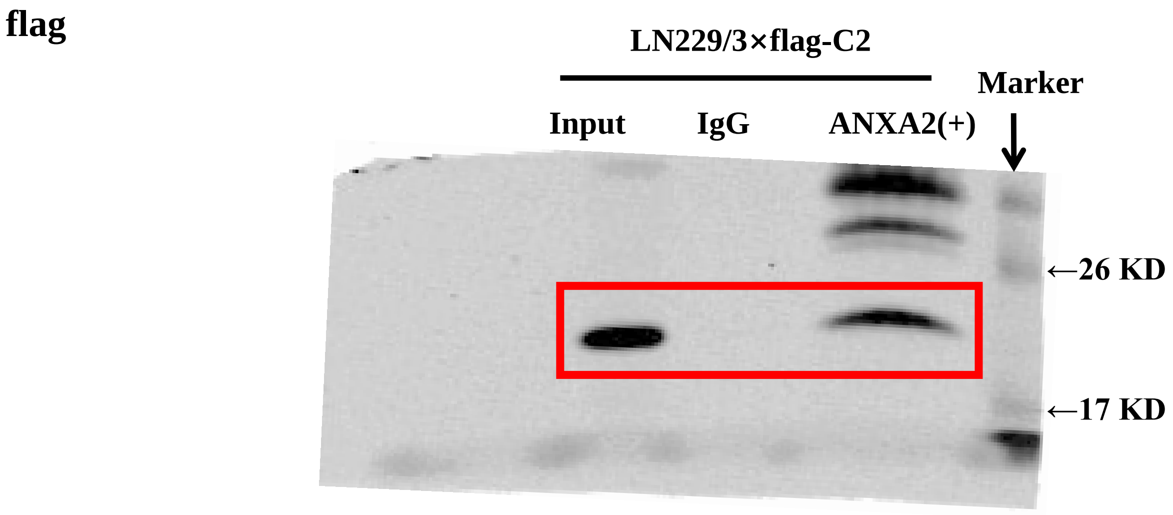

Full unedited gel for Figure 7b

ANXA2

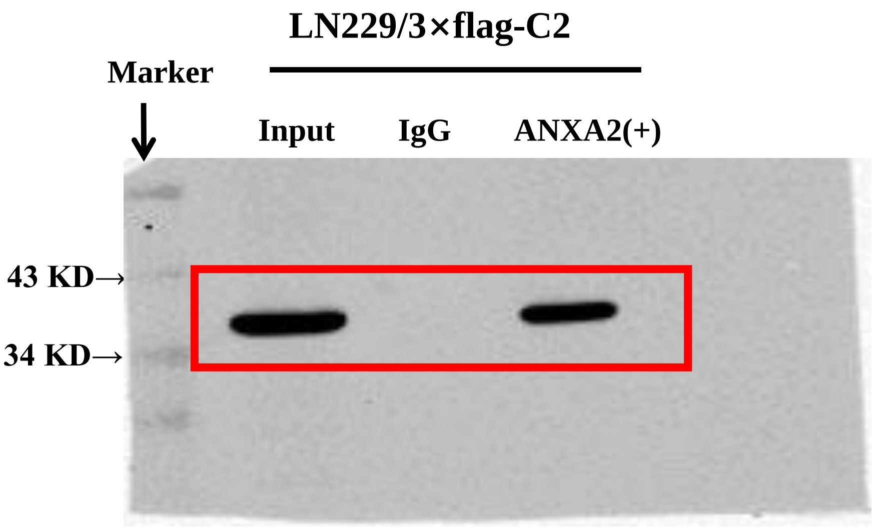

Full unedited gel for Figure 7c

N-cadherin

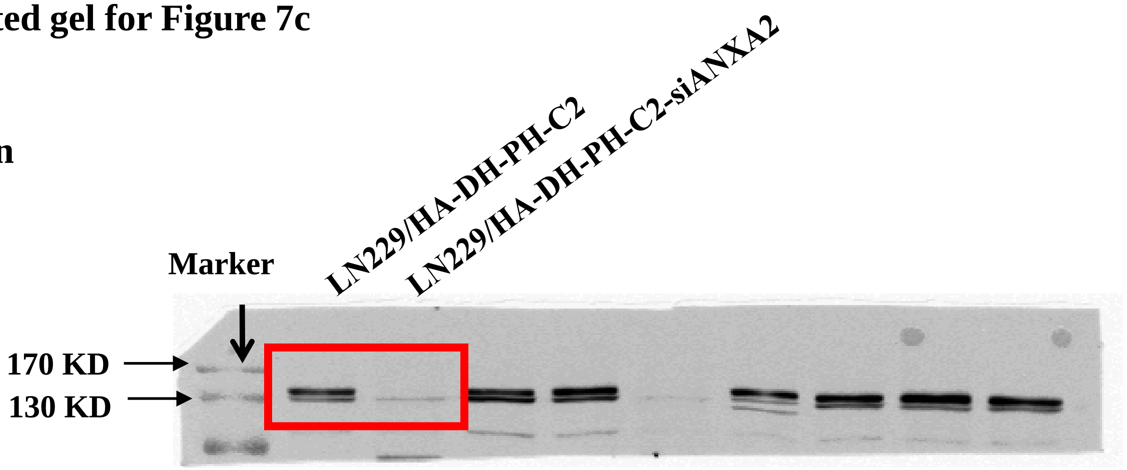

Full unedited gel for Figure 7c

ANXA2

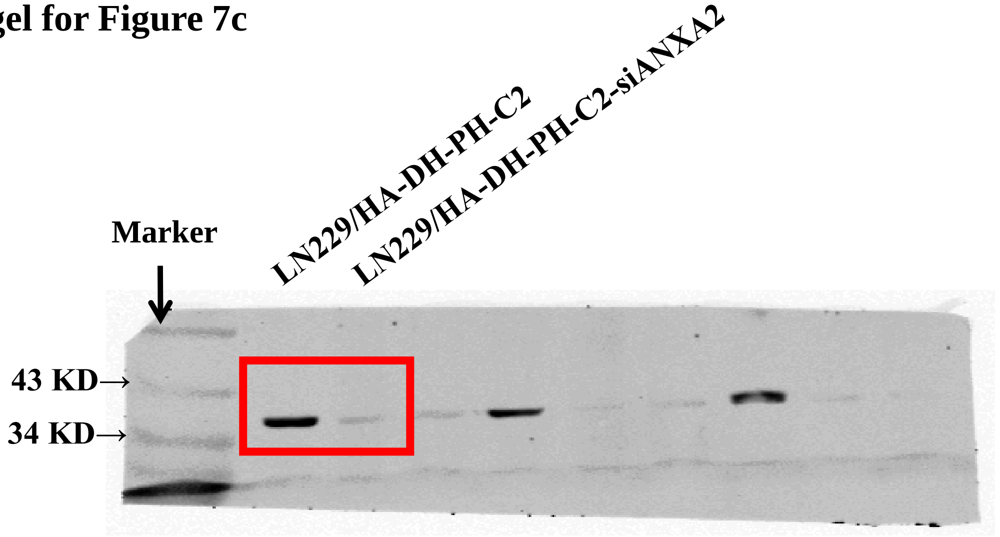

Full unedited gel for Figure 7c

Snail

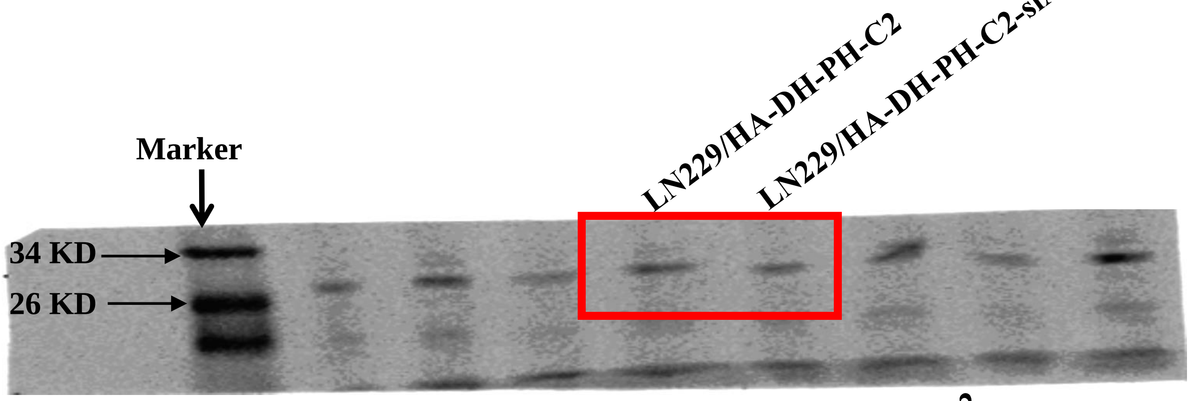

Full unedited gel for Figure 7c

Slug

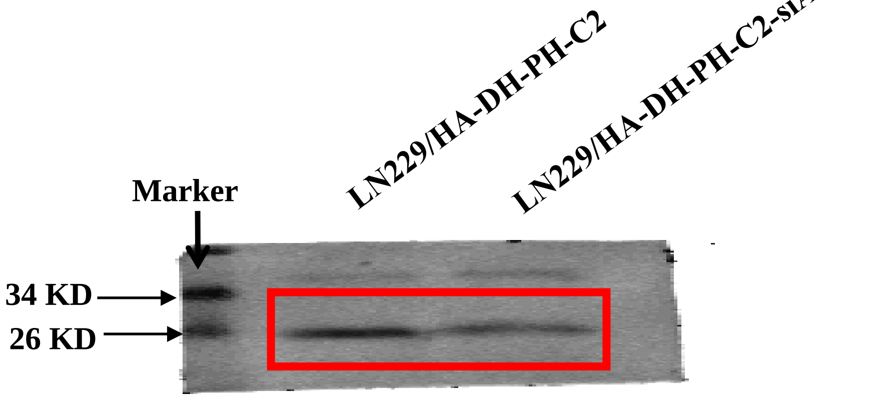

Full unedited gel for Figure 7c

Twist

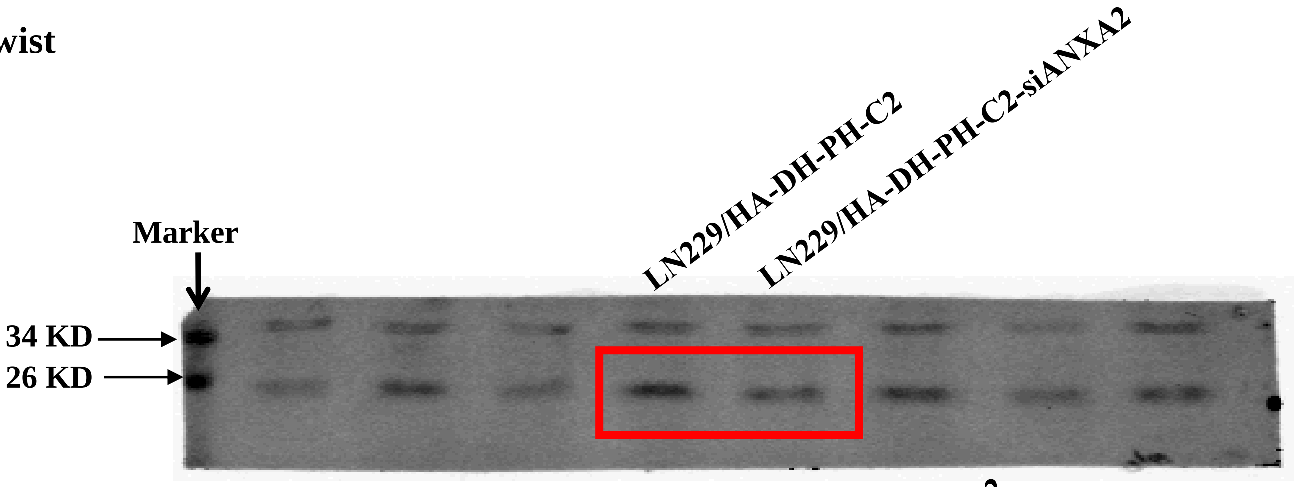

Full unedited gel for Figure 7c

β-actin

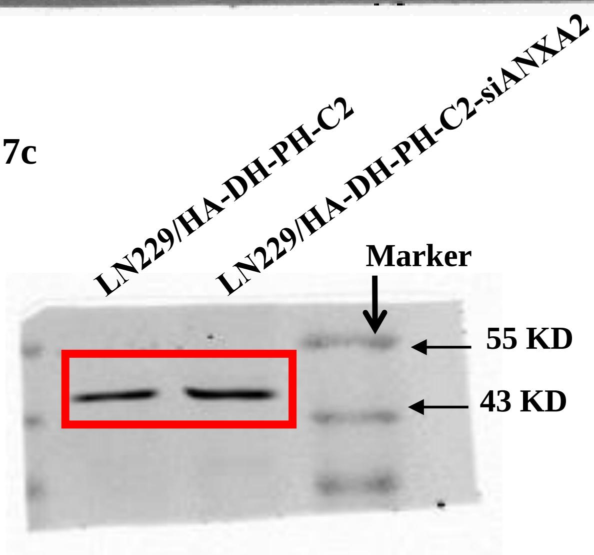

Full unedited gel for Figure 7h

β-tubulin

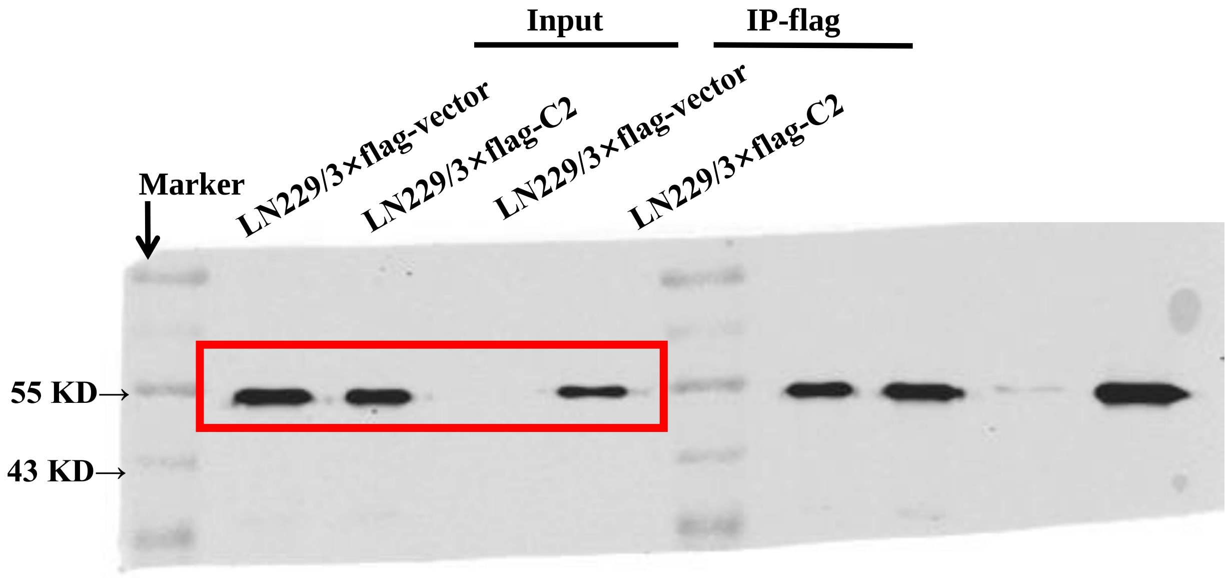

Full unedited gel for Figure 7h

flag

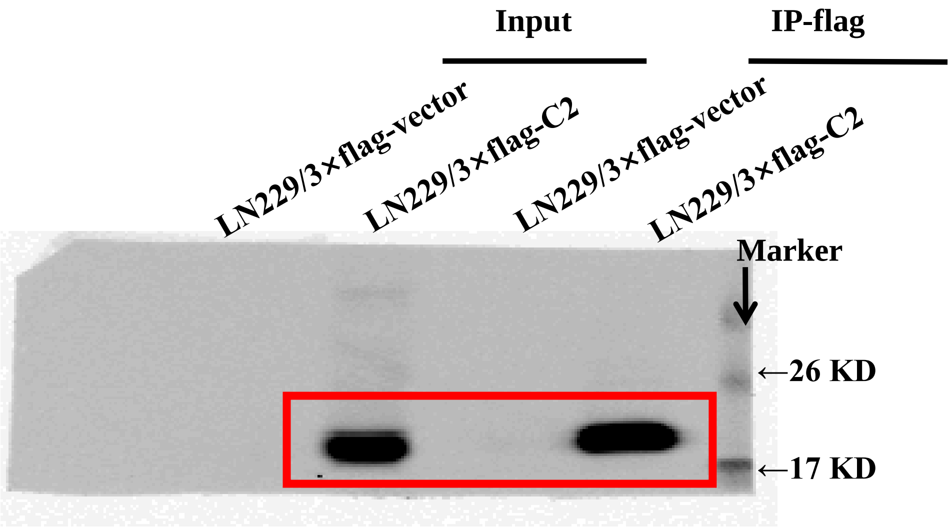

Full unedited gel for Figure 7i

flag

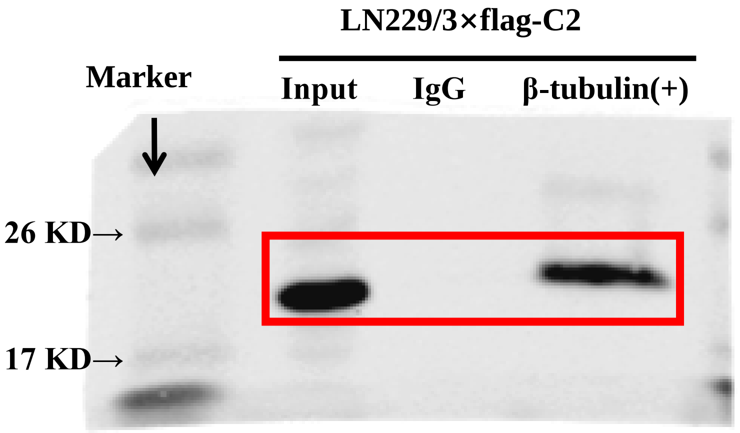

Full unedited gel for Figure 7i

β-tubulin

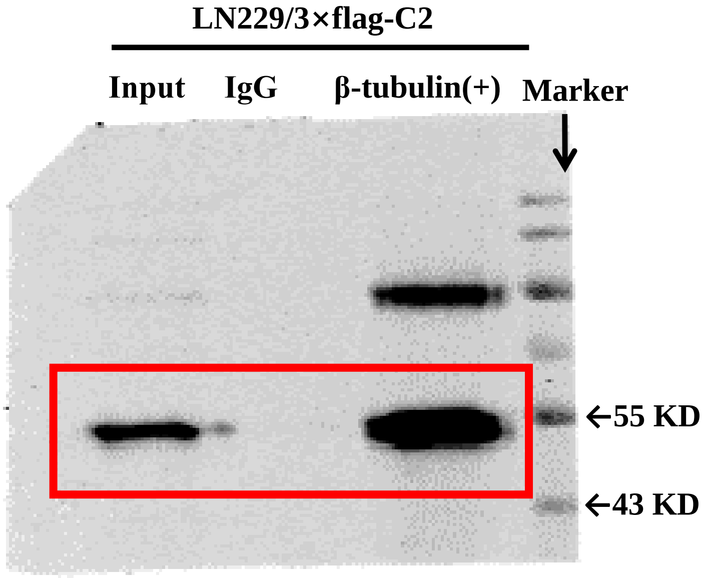

Full unedited gel for Figure 7k

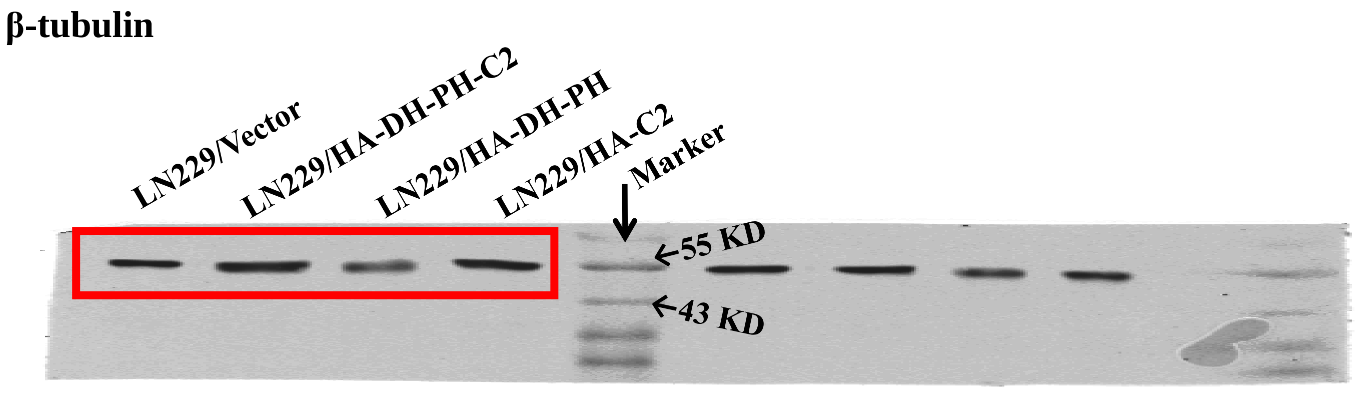

Full unedited gel for Figure 7k

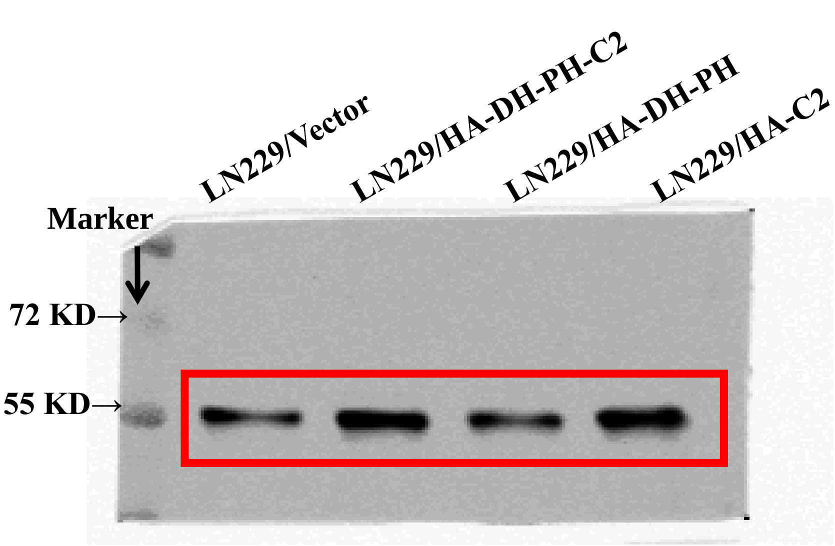

Full unedited gel for Figure 7k

TUBB4

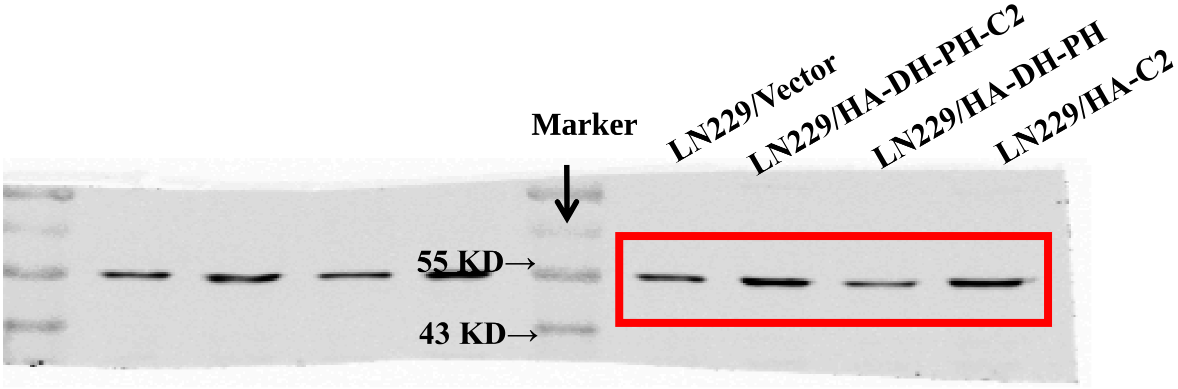

Full unedited gel for Figure 7k

β-actin

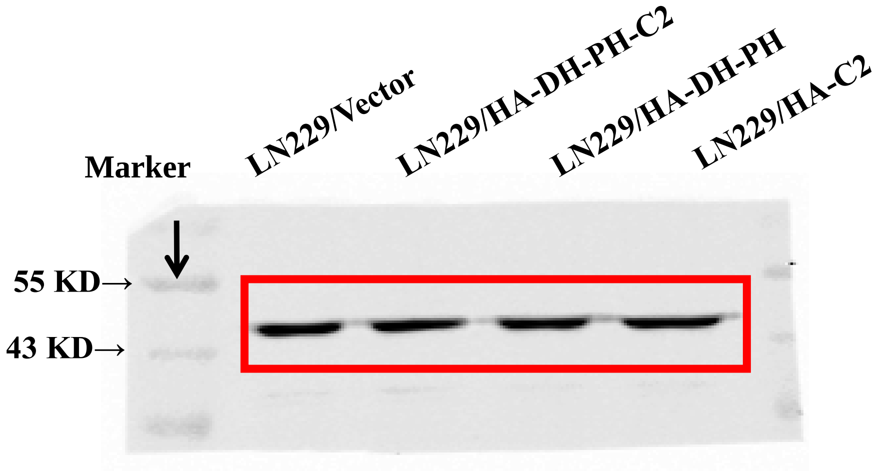

Full unedited gel for Figure 7l

N-cadherin

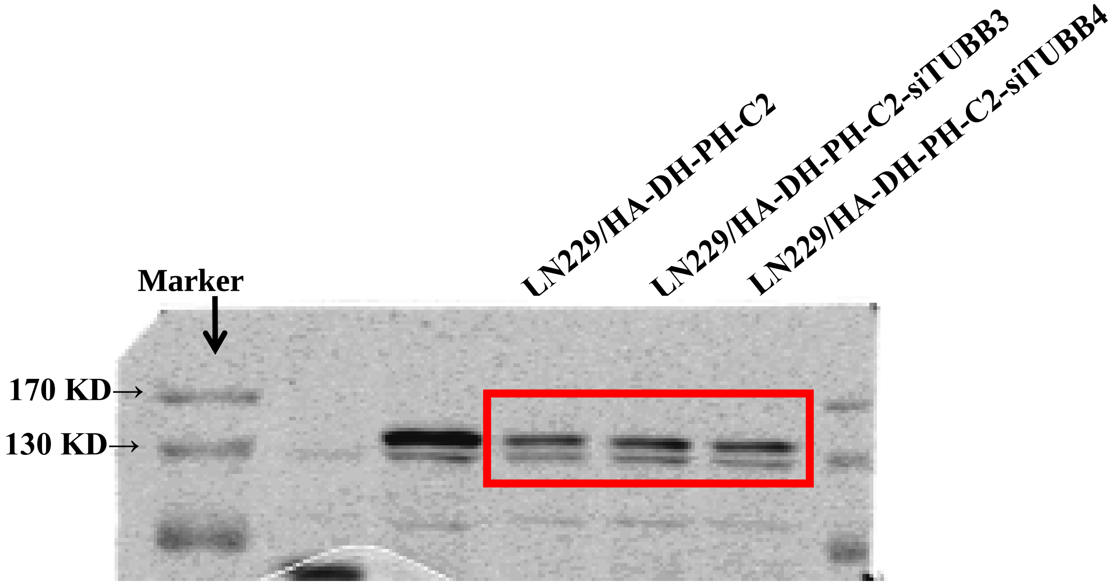

Full unedited gel for Figure 7l

TUBB3

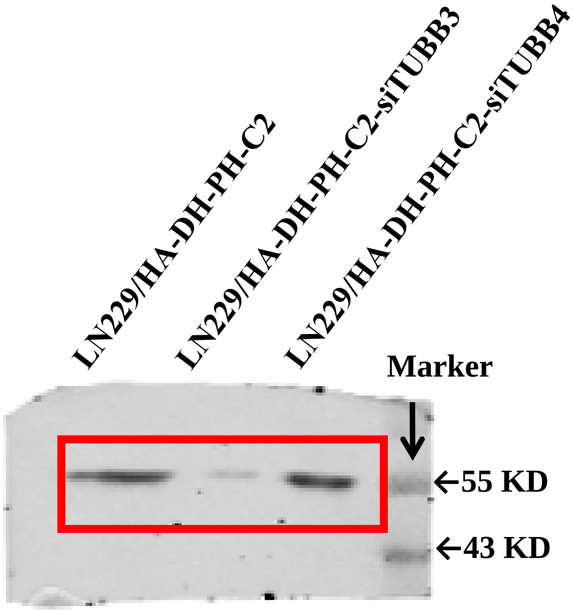

Full unedited gel for Figure 7l

TUBB4

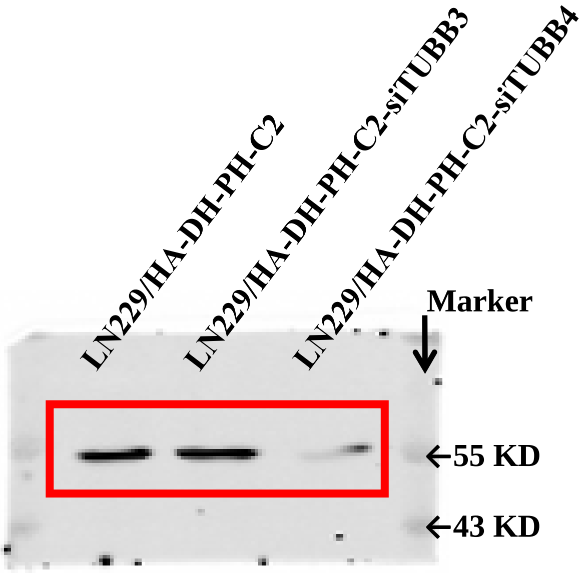

Full unedited gel for Figure 7l

β-actin

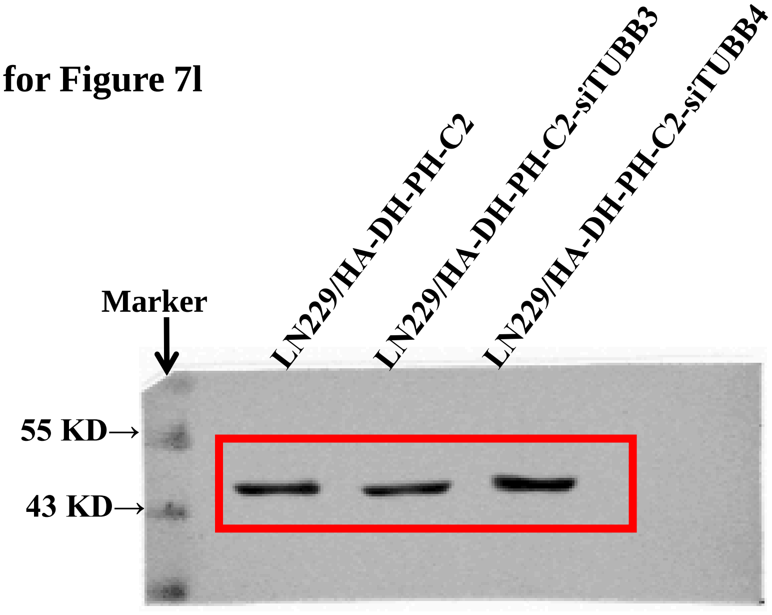

Full unedited gel for Supplementary Figure 1b

ITSN1-S

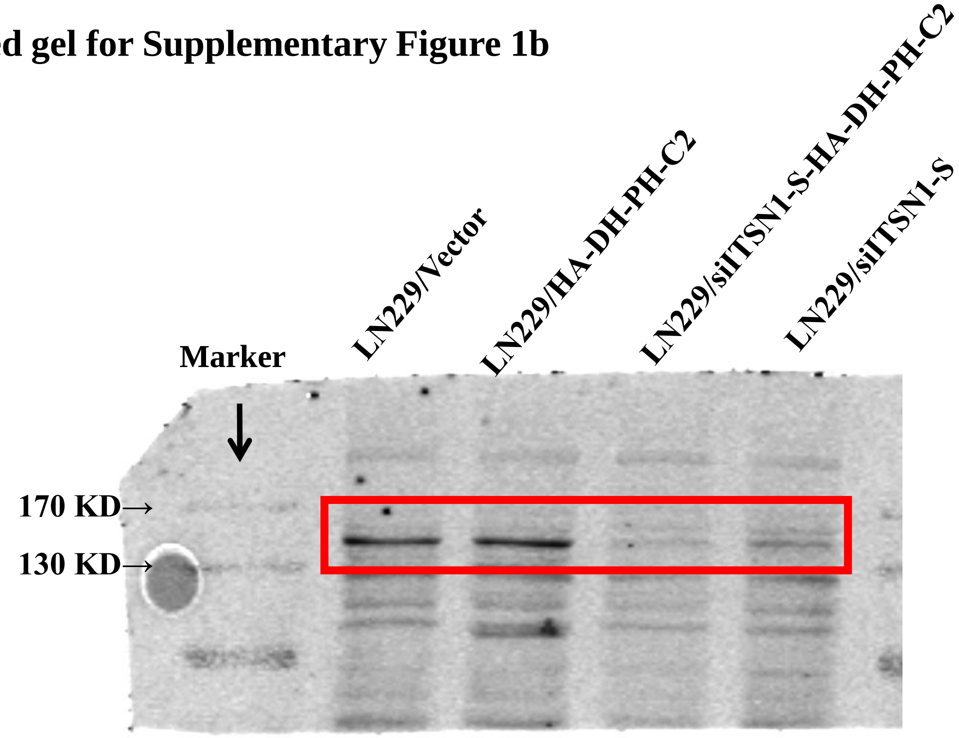

Full unedited gel for Supplementary Figure 1b

HA

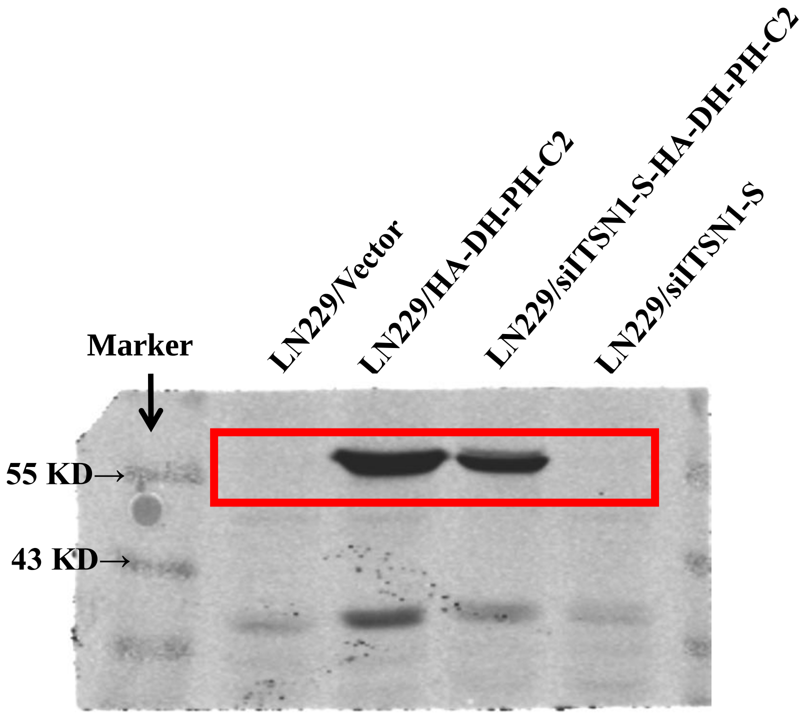

Full unedited gel for Supplementary Figure 1b

β-actin

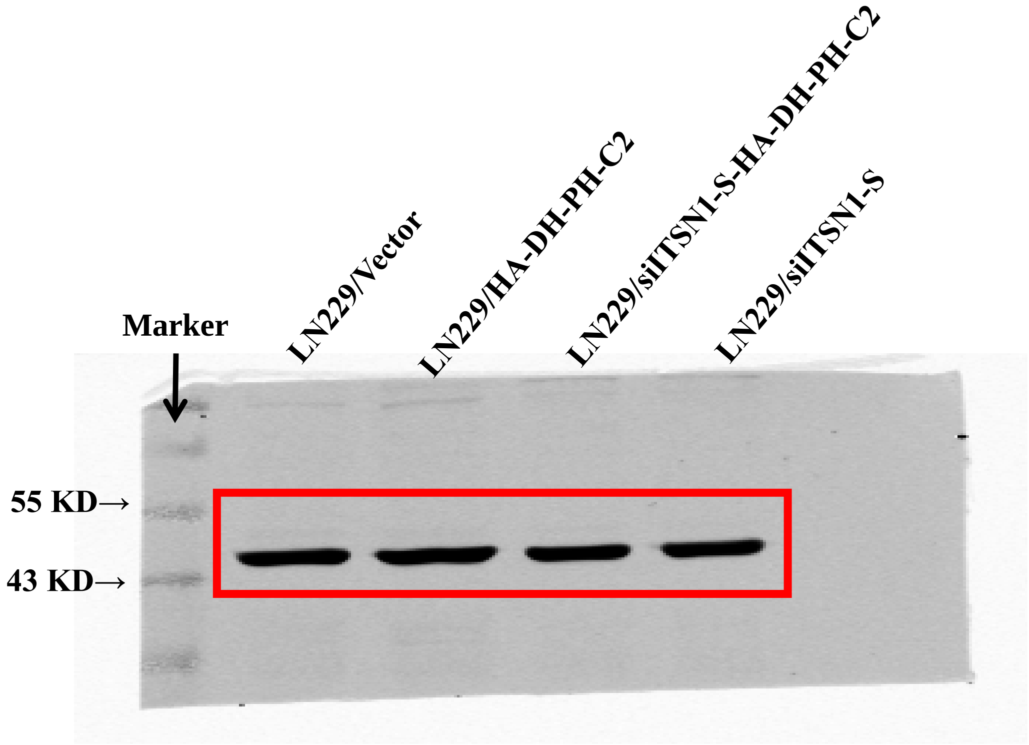

Full unedited gel for Supplementary Figure 2d

HA

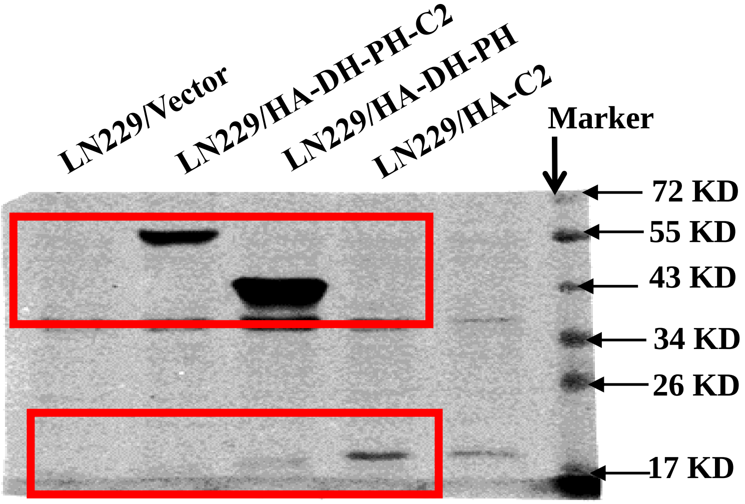

Full unedited gel for Supplementary Figure 2d

β-actin

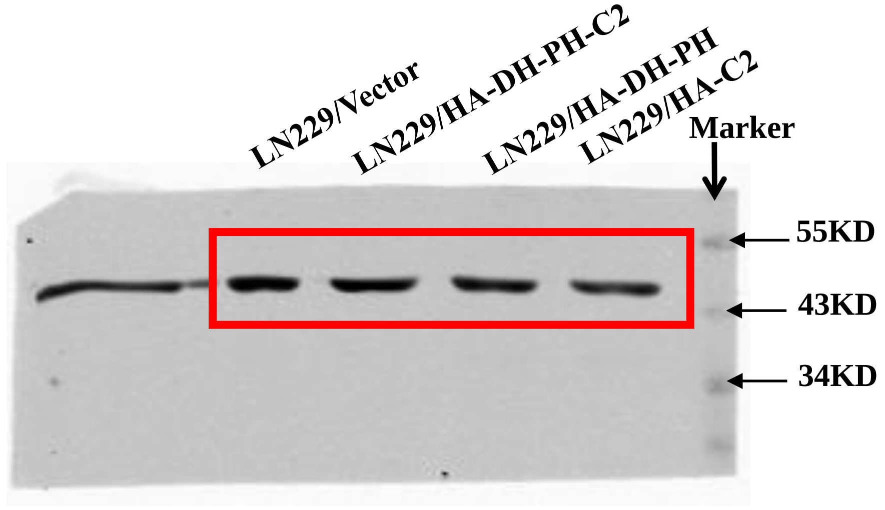

Full unedited gel for Supplementary Figure 3c

P-FAK

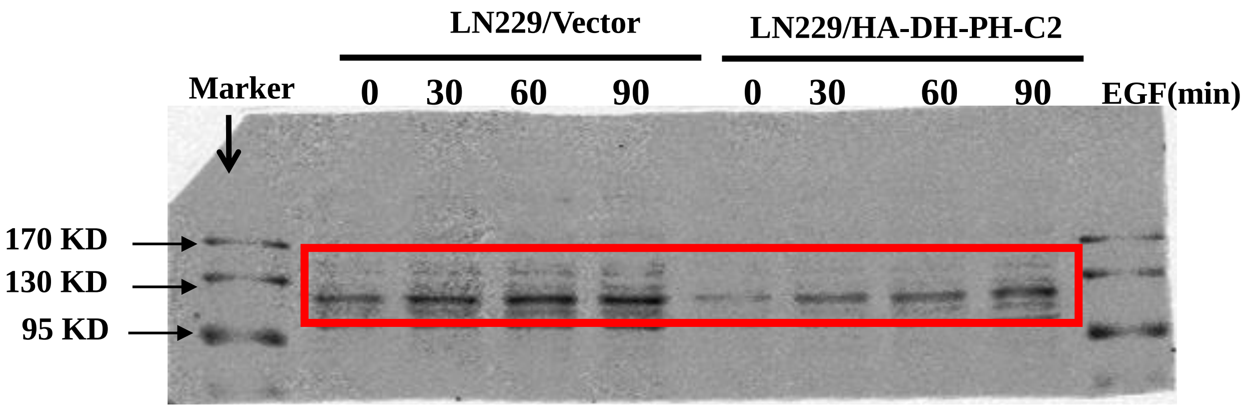

Full unedited gel for Supplementary Figure 3c

FAK

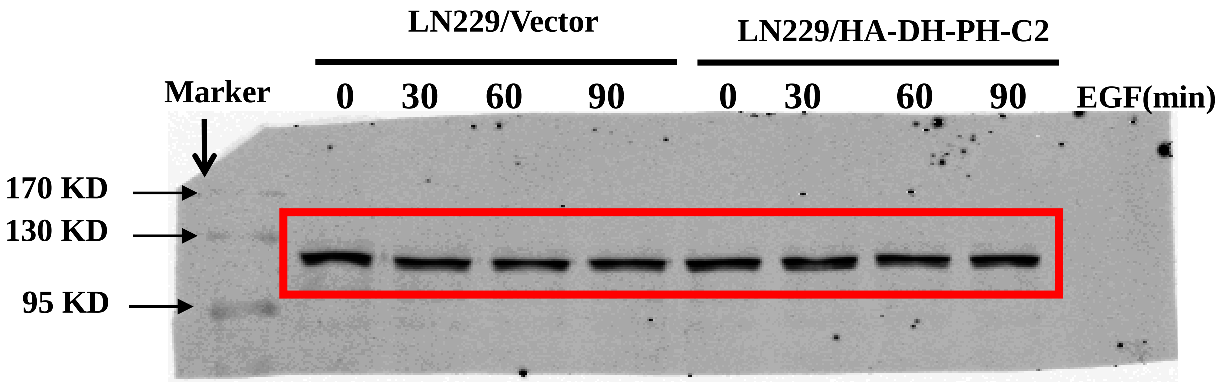

Full unedited gel for Supplementary Figure 3c

p-integrin β3

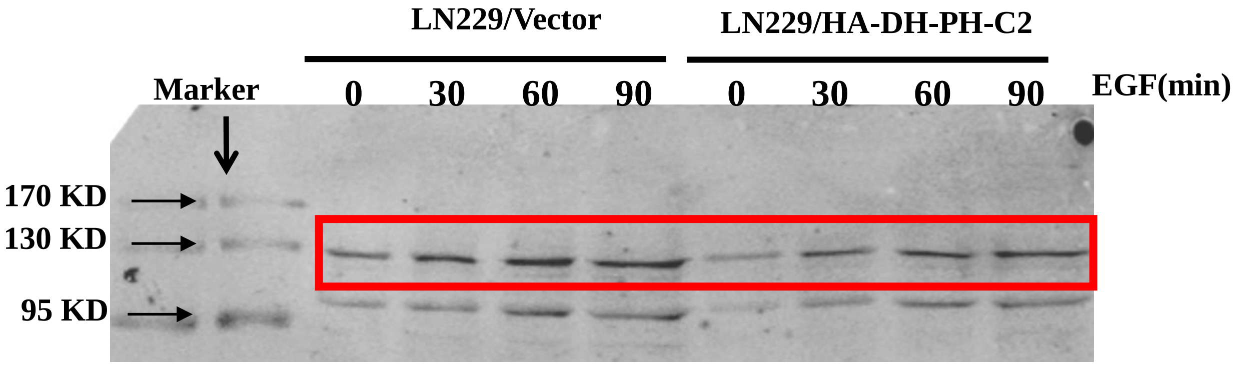

Full unedited gel for Supplementary Figure 3c

integrin β3

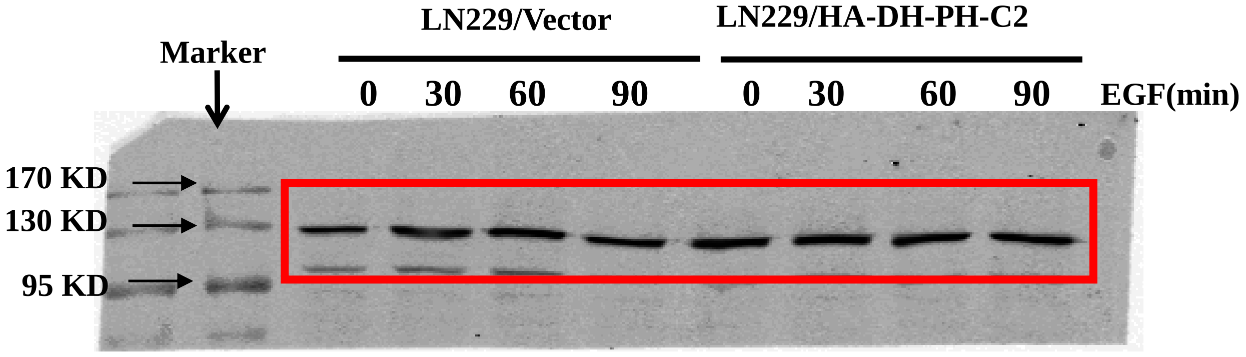

Full unedited gel for Supplementary Figure 3c

β-actin

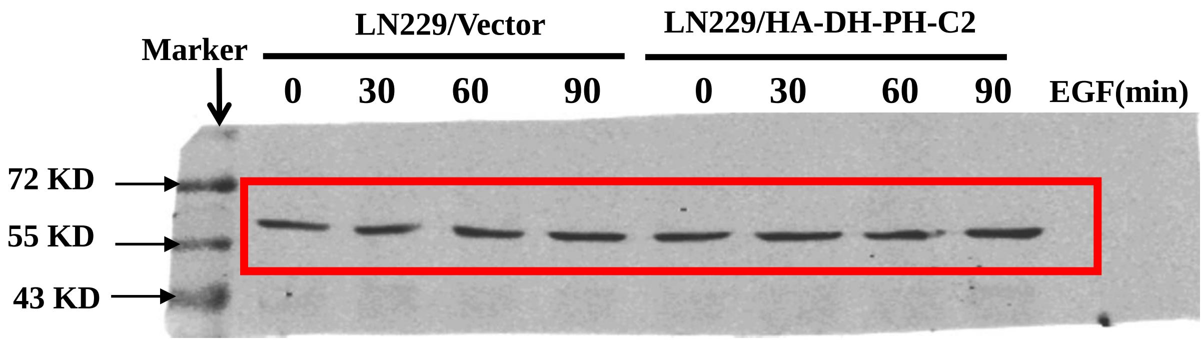

Full unedited gel for Supplementary Figure 3g

N-cadherin

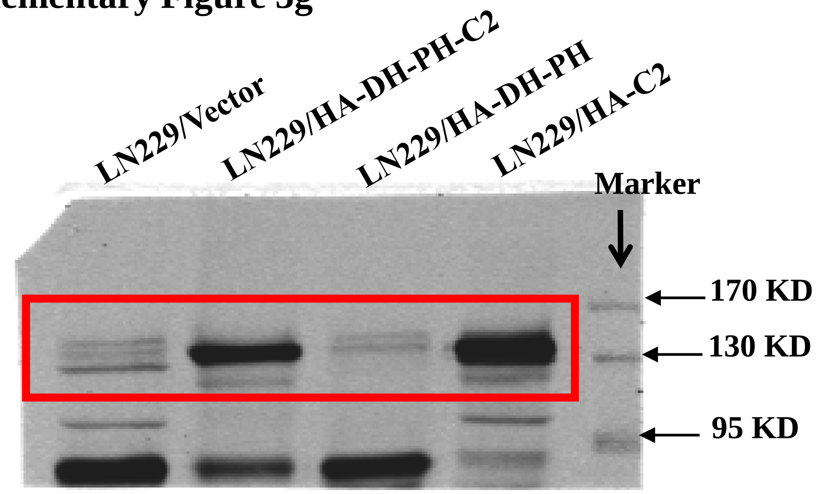

Full unedited gel for Supplementary Figure 3g

β-catenin

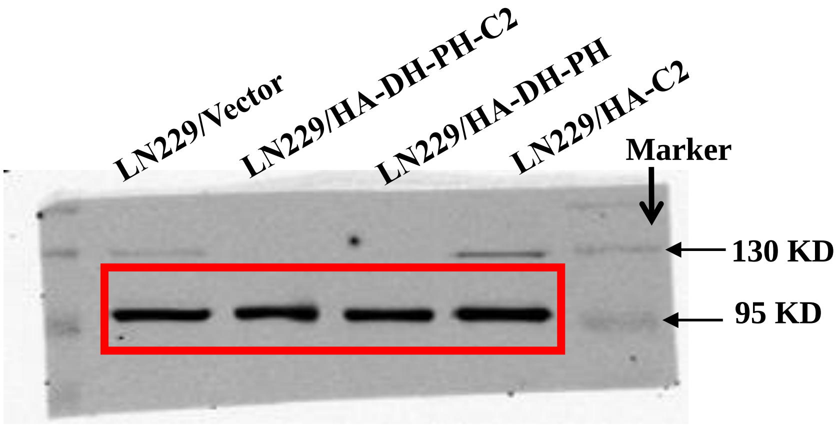

Full unedited gel for Supplementary Figure 3g

Snail

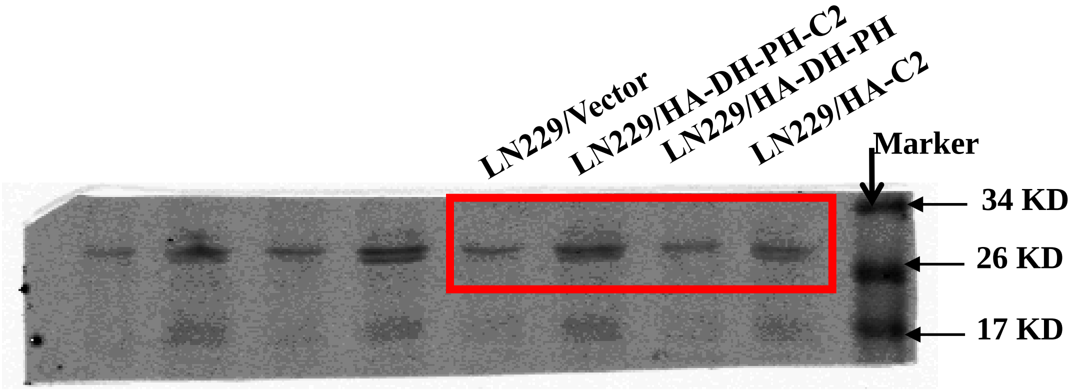

Full unedited gel for Supplementary Figure 3g

Slug

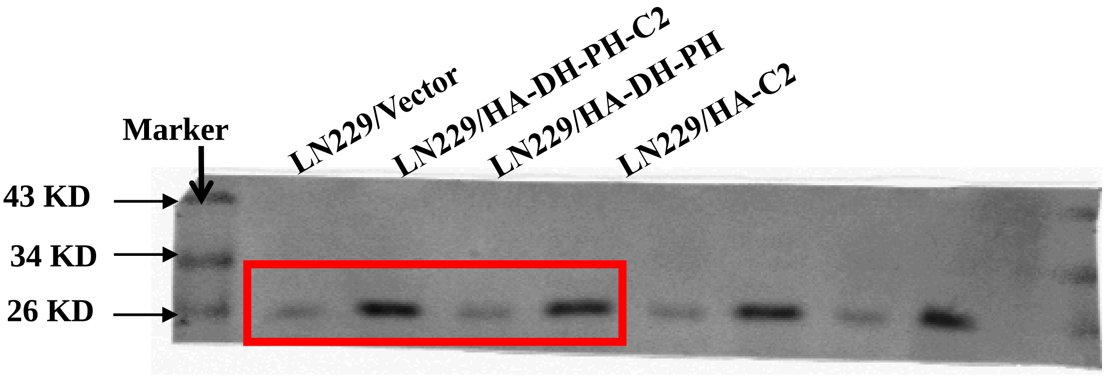

Full unedited gel for Supplementary Figure 3g

Twist

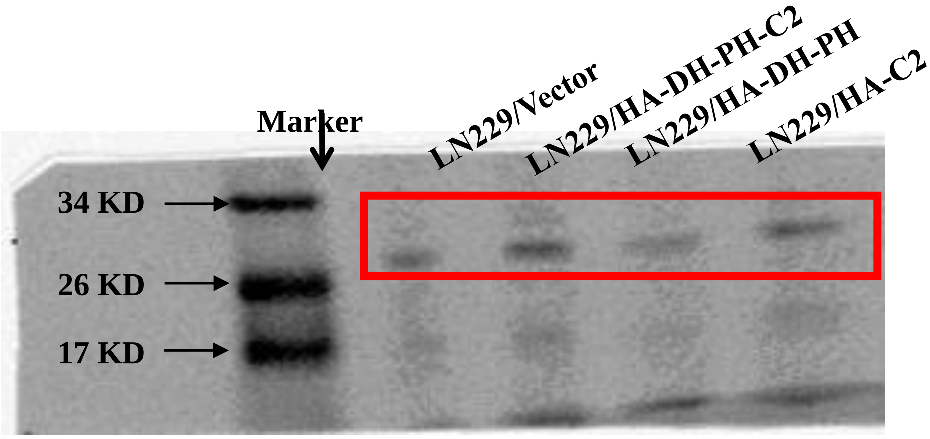

Full unedited gel for Supplementary Figure 3g

β-actin

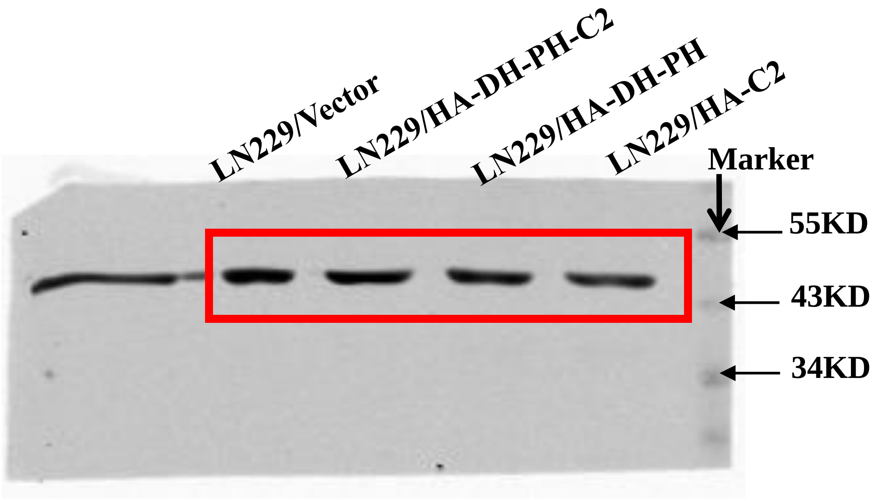

Supplement: Supplementary file 7 — Full gel images [file 41419_2019_1668_MOESM7_ESM.pdf]
